# Inhibition of Thiamine Diphosphate-Dependent Enzymes by Triazole-Based Thiamine Analogues

**DOI:** 10.1021/acsmedchemlett.3c00047

**Published:** 2023-04-11

**Authors:** Alex H. Y. Chan, Terence C. S. Ho, Imam Fathoni, Rebecca Pope, Kevin J. Saliba, Finian J. Leeper

**Affiliations:** † Yusuf Hamied Department of Chemistry, University of Cambridge, Lensfield Road, Cambridge CB2 1EW, U.K.; ‡ Research School of Biology, The Australian National University, Canberra, ACT 2601, Australia

**Keywords:** thiamine diphosphate, enzyme inhibition, metal-binding group, malaria

## Abstract

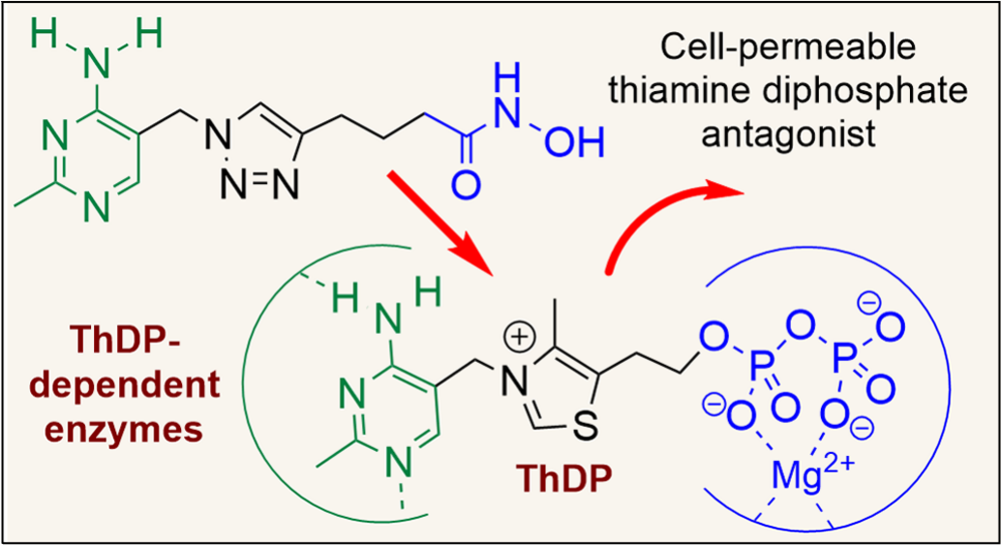

Thiamine is metabolized into the coenzyme thiamine diphosphate (ThDP). Interrupting thiamine utilization leads to disease states. Oxythiamine, a thiamine analogue, is metabolized into oxythiamine diphosphate (OxThDP), which inhibits ThDP-dependent enzymes. Oxythiamine has been used to validate thiamine utilization as an anti-malarial drug target. However, high oxythiamine doses are needed *in vivo* because of its rapid clearance, and its potency decreases dramatically with thiamine levels. We report herein cell-permeable thiamine analogues possessing a triazole ring and a hydroxamate tail replacing the thiazolium ring and diphosphate groups of ThDP. We characterize their broad-spectrum competitive inhibition of ThDP-dependent enzymes and of *Plasmodium falciparum* proliferation. We demonstrate how the cellular thiamine-utilization pathway can be probed by using our compounds and oxythiamine in parallel.

Thiamine **1** (vitamin B_1_) is essential for energy metabolism, and its deficiency leads to neurological disorders.^1−3^ Thiamine, being positively charged, requires transport into the cytoplasm, where it is converted into coenzyme thiamine diphosphate (ThDP) **2a** by thiamine pyrophosphokinase (TPK) (Figure 1A).^1−5^ ThDP-dependent enzymes include pyruvate dehydrogenase complex E1-subunit (PDHc E1), pyruvate decarboxylase (PDC), oxoglutarate dehydrogenase complex E1-subunit (OGDHc E1), pyruvate oxidase (PO), and transketolase (TK). Individual enzymes differ in substrate preferences and reactions catalyzed, but they all share similar ThDP binding sites. In mammalian tissues, ThDP is the major form of thiamine, but lesser amounts of free thiamine **1**, thiamine monophosphate, thiamine triphosphate **3** (ThTP), and adenosine thiamine triphosphate **4** (AThTP) are also found.^1−3^ These derivatives seem to have non-coenzyme roles:^1−3^ thiamine **1** is co-released with acetylcholine in acetylcholinergic neurons and seems to facilitate neurotransmission; ThTP **3** functions as a phosphate donor in a protein phosphorylation reaction; and AThTP **4** can inhibit poly-ADP-ribose polymerase 1, which is involved in DNA repair.^6^ Even ThDP may have non-coenzyme roles: elevated levels inhibit pyridoxal kinase and inhibit the binding of p53 to DNA. In bacteria, fungi, and plants ThDP also down-regulates its own biosynthesis by binding to riboswitches in the mRNA coding for biosynthetic enzymes.^1−3^

**Figure 1:**
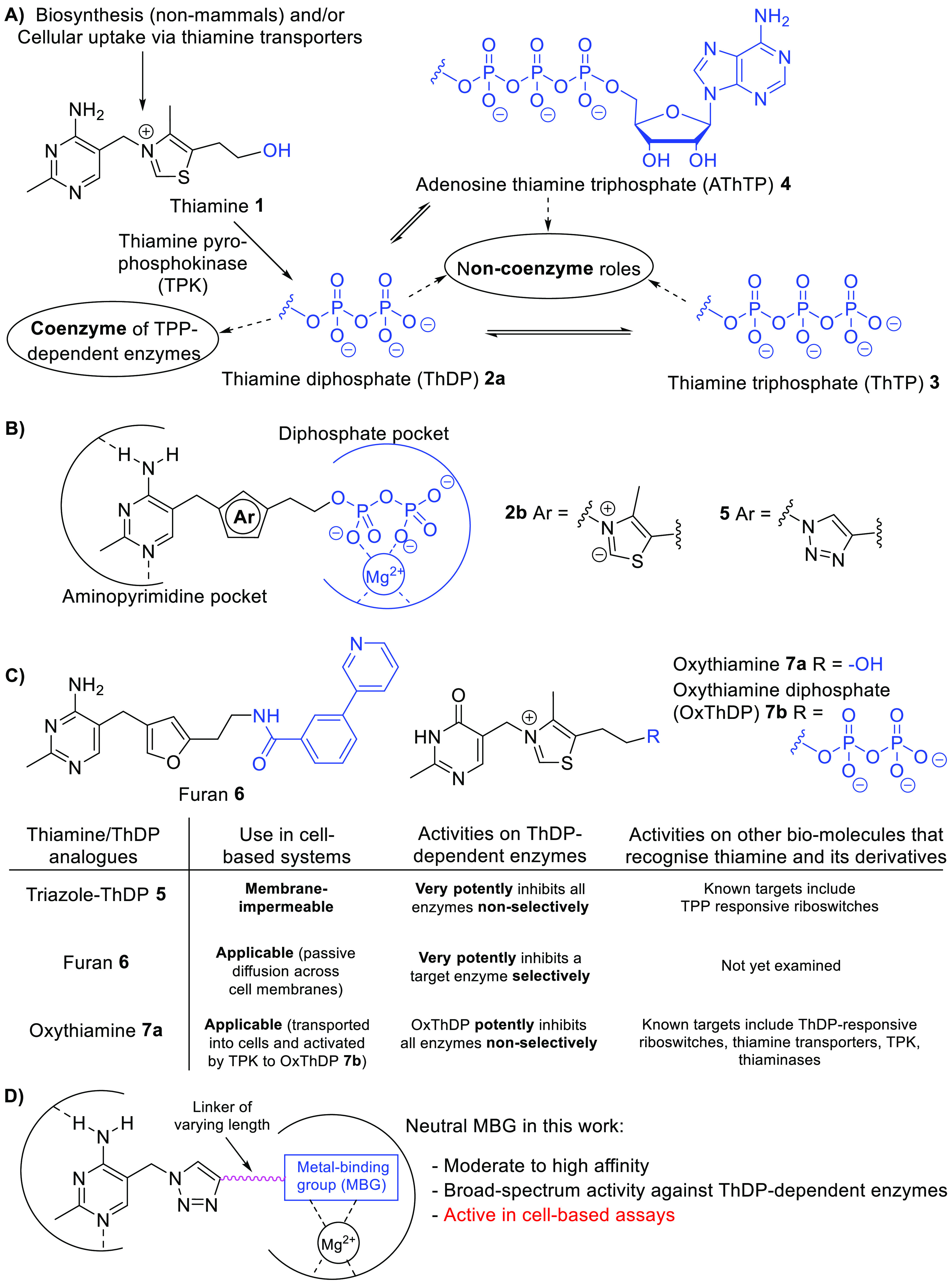
(**A**) Thiamine-utilization pathways. (**B**) Binding mode of **2b** and **5** in the ThDP pocket. (**C**) Comparison of the activities of thiamine/ThDP analogues. (**D**) Ligand design strategy.

A common method to study the coenzyme role of thiamine is to use thiamine/ThDP analogues (Figure 1B), both synthetic^3,7−19^ and naturally occurring,^20,21^ as ThDP-competitive inhibitors of ThDP-dependent enzymes. Figure 1C summarizes the three main types of thiamine/ThDP analogues that have been employed. The first type,^7,8^ represented by triazole-ThDP **5**,^8^ features a neutral central ring in place of the ThDP’s positive thiazolium ring **2a**, which abolishes the catalytic activity. These ThDP analogues are potent ThDP-dependent enzyme inhibitors as the neutral central ring captures the strong stabilizing interactions between the enzyme’s hydrophobic region and the catalytically active high-energy ThDP-ylide **2b** (Figure 1B). They inhibit ThDP-dependent enzymes non-selectively (binding in place of the cofactor) but are not cell-permeable due to the polyanionic diphosphate.^20^ The second type,^9−13^ represented by furan **6**,^13^ also features a neutral central ring but bears a neutral tail replacing the diphosphate. This neutral tail compensates for the loss of the ionic interaction between the diphosphate and the Mg^2+^ ion by forming interactions with surrounding residues in the diphosphate pocket of PDHc E1. Since these interactions are specific to PDHc E1, **6** is a potent and PDHc-selective inhibitor. It is membrane-permeable and can be used to study the cellular role of PDHc. The third type,^14−21^ represented by oxythiamine **7a**, is a thiamine analogue featuring a modified pyrimidine ring. **7a** is a prodrug; it enters cells, probably via thiamine transporters, and is activated by TPK to OxThDP **7b**, which inhibits ThDP-dependent enzymes.^1−3^ Triazole-ThDP **5** and OxThDP **7b** are known ligands for ThDP riboswitches which recognize both the aminopyrimidine and diphosphate moieties.^22^

Interruption of the thiamine-utilization pathway can result in diseases such as diabetes and neurodegeneration and is also found in many cancers.^1−4^ To understand these diseases, compounds causing thiamine deficiency within the cells can be used. Oxythiamine **7a** is the most widely applied tool for inducing thiamine deficiency in cells and *in vivo*.^1−3,14−18^ We reported previously that oxythiamine inhibits the *in vitro* proliferation of *Plasmodium falciparum*.^15^ However, oxythiamine **7a** has weaknesses as a probe: 1) its positively charged thiazolium ring can result in poor pharmacokinetic properties and degradation by thiaminases, so high oral doses are needed *in vivo*;^15−17^ 2) the diphosphate moiety of **7b** can be hydrolyzed by ThDP-phosphatases;^23^ 3) its similarity to ThDP could allow it to participate in non-coenzyme roles of ThDP **2** or (after appropriate modification of the diphosphate) of **3** and **4** (Figure 1A);^1,2^ and 4) the required intracellular activation of **7a** to **7b** makes its action highly sensitive to the levels of thiamine. As an example of this last weakness, oxythiamine inhibits *in vitro* proliferation of the 3D7 strain of *Plasmodium falciparum* with an IC_50_ value of 11 μM in the absence of extracellular thiamine, but the IC_50_ value increased 470-fold (to 5.2 mM) with added thiamine (2.97 μM).^15^ We attribute the significant reduction of the anti-plasmodial effect with increasing thiamine levels to the competition with thiamine/ThDP at all stages of thiamine utilization, cell entry, pyrophosphorylation, and enzyme binding.

In this paper we prepare a series of neutral triazole-based thiamine analogues (Figure 1D), resulting in the discovery of thiamine analogues with inhibitory activity against multiple ThDP-dependent enzymes. The anti-plasmodial activity of the analogues are assessed and compared to that of oxythiamine. Lacking the diphosphate, they are unlikely to be recognized by other ThDP-binding proteins or riboswitches. Also, they are not dependent on thiamine transporters or pyrophosphorylation by TPK, which reduces their sensitivity to the extracellular level of thiamine. We envision that these compounds will be useful tools to study the coenzyme roles of ThDP, complementing oxythiamine in inducing the effects of thiamine deficiency.

Triazole-ThDP **5** is one of the most potent inhibitors of ThDP-dependent enzymes, with *K*_I_ for PDC from *Zymomonas mobilis* of 30 pM.^8,24^ Based on this, our pharmacophore model of thiamine/ThDP analogues (Figure 1D) consists of the aminopyrimidine-CH_2_–triazole moiety joined to a neutral metal-binding group (MBG) by a linker of variable length for optimal positioning of the MBG. The use of a neutral MBG in place of the polyanionic diphosphate was for membrane-permeability.^25^ Seven different MBGs were tested in this study, adapted from other reported metalloenzyme inhibitors (Scheme 1).^26^ Most MBGs were attached by linkers of three different lengths. All ligands were efficiently synthesized in 2–5 steps from a common precursor azide **8**, in turn obtained in a single-step from inexpensive thiamine.^8^ OxThDP was synthesized as reported.^18^

**Scheme 1:**
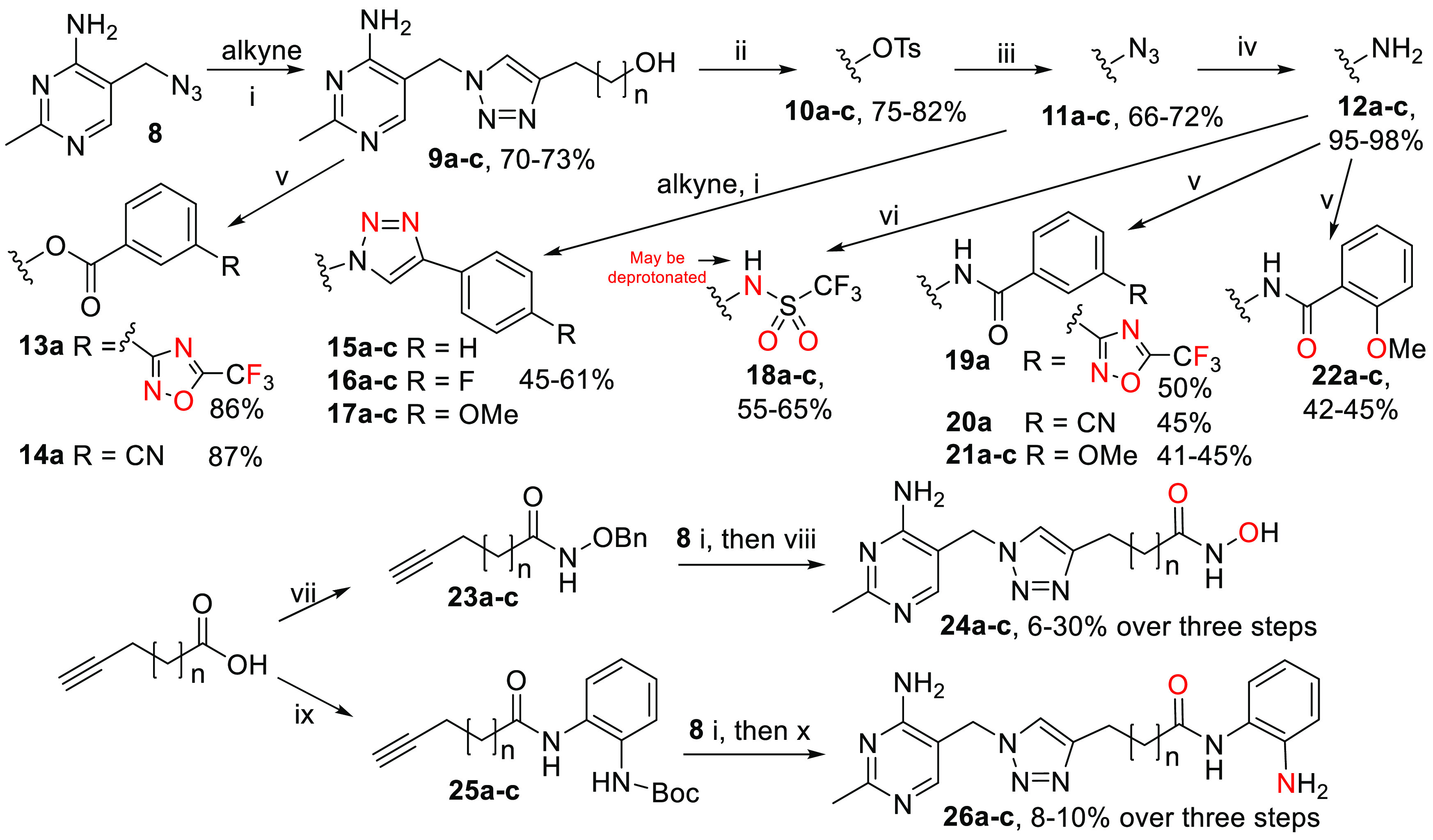
Synthesis of Triazole-Based Thiamine Analogues *Reagents and conditions*: (i) CuSO_4_·5H_2_O, sodium ascorbate, *t*-BuOH, H_2_O, RT; (ii) TsCl, pyridine, RT; (iii) NaN_3_, DMF, RT; (iv) H_2_(*g*), 10% Pd/C, MeOH, RT; (v) carboxylic acid, DCC, DMAP, DMF, RT; (vi) Tf_2_O, pyridine, 35 °C; (vii) NH_2_OBn.HCl, CDI, DMF, THF, RT; (viii) BCl_3_, DCM, RT; or H_2_(*g*), 10% Pd/C, MeOH, RT; (ix) *N*-Boc-1,2-phenylenediamine, DCC, DMAP, DMF, 40 °C; (x) TFA, DCM, RT. For all compounds: **a**, *n* = 1; **b**, *n* = 2; **c**, *n* = 3. Potential metal-binding atoms of each metal-binding group (MBG) are highlighted.

To demonstrate widespread inhibition of ThDP-dependent enzymes, we chose four enzymes across three kingdoms—porcine PDHc E1, *Saccharomyces cerevisiae* PDC, *E*. *coli* OGDHc E1, and *Aerococcus viridans* PO. The different reactions catalyzed, preferred substrates (Table 1), and primary sequences reflect the structurally and functionally diverse members of the family.

**Table 1:**
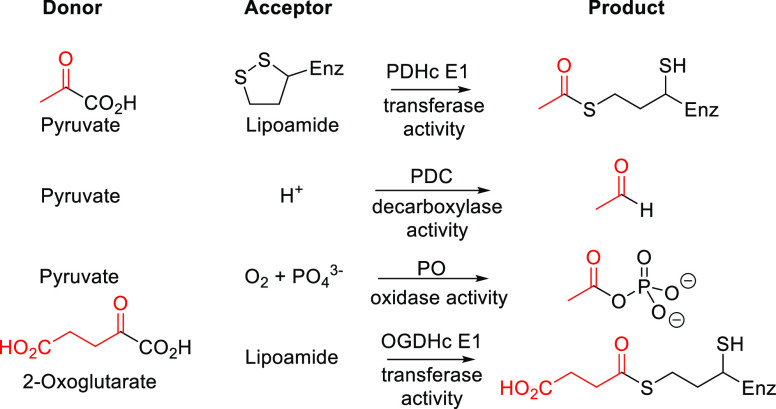

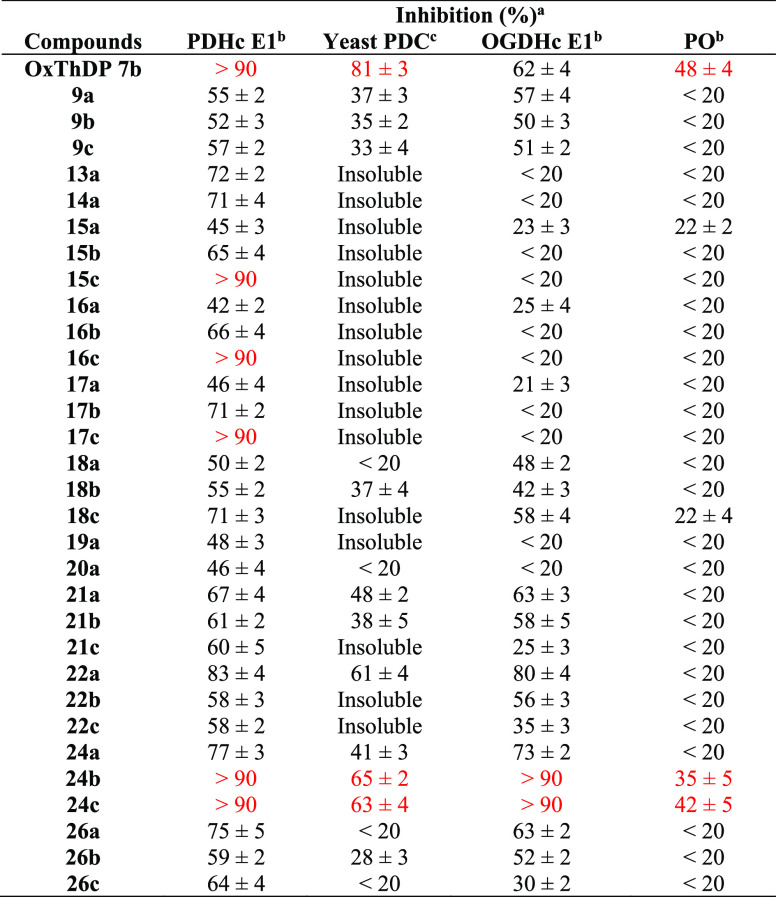
Comparison of Assay Enzymes and Preliminary Screening Data

a Data are the means of measurements in three technical replicates.

b [Compound] = 250 μM, [ThDP] = 50 μM.

c [Compound] = 1.5 mM, [ThDP] = 0.3 mM; compounds insufficiently soluble were excluded. (The *K*_M_ of yeast PDC for ThDP was found to be 11–23 μM, so a high [ThDP] was needed to fully saturate the enzyme.)

The 32 compounds shown in Table 1 were subjected to preliminary screening for inhibition of the four enzymes. Free alcohols **9a**–**c**, which lack the MBG but retain the aminopyridine-CH_2_–triazole moiety, were included as reference compounds. OxThDP **7b** was used as a positive control. The compounds showed a wide range of percentage inhibition on PDHc E1, PDC, and OGDHc E1; they were mostly more potent than the alcohols **9a**–**c**, indicating that the modified tail can contribute to binding. Surprisingly, most ligands were inactive on PO, with only **7b**, **15a**, **18c**, **24b**, and **24c** inhibiting weakly. Five inhibitors particularly attracted our attention: bis-triazoles **15**–**17c** and hydroxamates **24b**,**c**. The two hydroxamates showed inhibitory activities comparable to that of OxThDP **7b** and markedly greater than those of **9a**–**c** for all four enzymes; thus they are truly multi-targeting ligands, warranting further characterization. On the other hand, **15–17c** were very selective, potent inhibitors of PDHc E1.

The remaining compounds were not potent enough to warrant further biological testing. However, some docking studies were performed to explain the observed inhibitory effects (Figures S1–3). The ThDP-competitive relationship of the inhibitors was validated by repeating the assays at increased [ThDP]:[compound] ratio (Table 2). As expected, the percentage inhibition by **7b**, **15–17**, and **24b** and **c** all decreased, consistent with competitive inhibition. These assays showed that **17c** is the strongest binder among the bis-triazole series **15**–**17**, selective to PDHc.

**Table 2:** Inhibitory Data of Bis-triazoles **15–17** and Hydroxamates **24b,c** under Increased ThDP Levels

	**Inhibition (%)**^a^			
**Compound**	**PDHc E1**^b^	**Yeast PDC**^c^	**OGDHc E1**^b^	**PO**^b^
**OxThDP 7b**	75 ± 3	63 ± 3	41 ± 4	32 ± 5
**15a**	<20	Insoluble	<20	<20
**15b**	40 ± 4	Insoluble	<20	<20
**15c**	75 ± 4	Insoluble	<20	<20
**16a**	<20	Insoluble	<20	<20
**16b**	43 ± 2	Insoluble	<20	<20
**16c**	79 ± 2	Insoluble	<20	<20
**17a**	<20	Insoluble	<20	<20
**17b**	45 ± 3	Insoluble	<20	<20
**17c**	82 ± 4	Insoluble	<20	<20
**24b**	70 ± 3	42 ± 4	78 ± 3	27 ± 4
**24c**	73 ± 4	37 ± 4	82 ± 2	24 ± 4

a Data are the means of measurements in three technical replicates. Compared to Table 1, the assays here were conducted with an increase in [ThDP]:[inhibitor] from 1:5 to 1:2.

b [Compound] = 250 μM, [ThDP] = 125 μM.

c [Compound] = 1500 μM, [ThDP] = 750 μM; compounds insufficiently soluble were excluded.

*In silico* docking studies into the ThDP-binding pocket of human PDHc E1 (Figures 2 and S4–S7) suggested **7b** and **24b** and **c** overlay well with ThDP, with the same V-shaped conformation of the aminopyridine-CH_2_–thiazole moiety. Hydroxamates **24b** and **c** showed a non-ionic, bidentate metal-binding pose (Figure 2C). For bis-triazole **17c**, docking studies suggested that two of the triazole nitrogen atoms interact with the Mg^2+^ (Figure 2D). **17c**, with the *p*-OMe group, seemed to be a better inhibitor than **15c** or **16c** because the O of the OMe group hydrogen bonds to the side chain of a glutamine residue. The *in silico* studies also suggested that the selectivity of **15**–**17** for PDHc E1 is because the longer side-chains are too bulky to be accommodated in the smaller diphosphate pockets in OGDHc E1, PDC, and PO (Figure S7).

**Figure 2:**
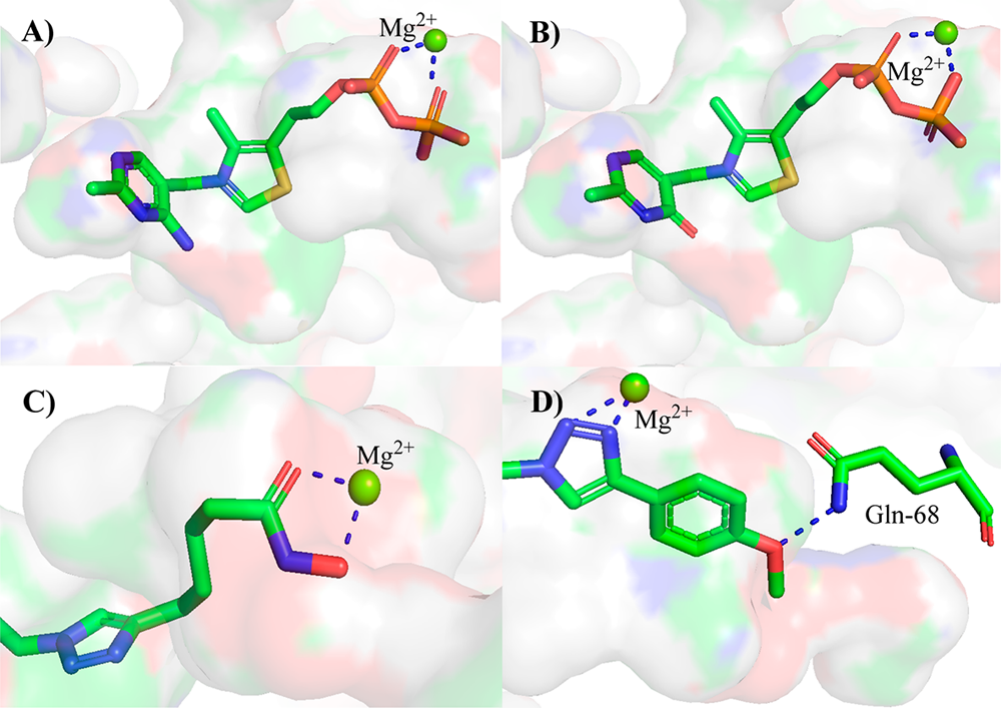
***In silico*****prediction of interactions with human PDHc E1**. (**A**) ThDP **2a**. (**B**) OxThDP **7b**. (**C**) Hydroxamate **24c**. (**D**) Bis-triazole **17c**.

Full IC_50_ determinations for bis-triazole **17c** and hydroxamates **24b**,**c** showed that they inhibited ThDP-dependent enzymes in a dose-dependent manner (Figure 3A–D). As ThDP-competitive ligands, their *K*_I_ value, affinity relative to ThDP, and ligand efficiency (L.E.) are shown in Figure 3E. L.E., measuring the binding energy of a ligand to its target (in kcal mol^–1^ derived from the *K*_I_ value) per heavy atom of the ligand, is a widely applied metric in medicinal chemistry;^27^ drug discovery efforts often aim to develop clinical candidates with L.E. > 0.3.^27,28^ Bis-triazole **17c** showed the best inhibition of PDHc E1, with a *K*_I_ of 30 nM; as it lacks activity on the other three enzymes, **17c** is an efficient, selective inhibitor of PDHc. Both hydroxamates, **24b** (L.E. = 0.50) and **24c** (L.E. = 0.48), are extremely efficient inhibitors of PDHc and very good inhibitors of the other three enzymes, too. The affinities of hydroxamates **24b** and **c** and OxThDP **7b** for PDHc E1 are all comparable to that of ThDP (Table 2 and Figure 3), which is in line with the findings of others on OxThDP.^2,3^ The molecular properties of hydroxamates **24b** and **c** (Table S1) are predicted to be in the range preferred for drugs,^27,28^ so they should be active on intact live cells.

**Figure 3:**
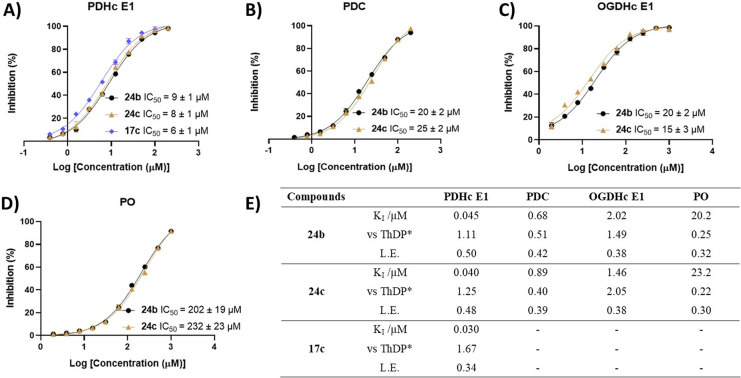
**Inhibition of ThDP-dependent enzymes by 17c and 24b,c.** IC_50_ values determined at [ThDP] (**A, B**) 10 μM, (**C**) 30 μM, and (**D**) 50 μM. Data are the means of measurements in three technical replicates. Where error bars are not visible they are smaller than the symbol used. The PDC used here was bacterial (from *Zymomonas mobilis*) due to the better assay conditions that avoid solubility issues. (**E**) *K*_I_ values and ligand efficiencies (L.E.). *K*_I_ values are determined according to the *K*_M_ for ThDP of the respective enzymes. *Affinity of the inhibitor versus that of ThDP (*i*.*e*. [ThDP]/IC_50_ or *K*_M(ThDP)_/*K*_I_).

Next the effects of the thiamine/ThDP analogues on *in vitro* proliferation of the 3D7 strain of *P*. *falciparum* were determined. Infected erythrocytes were treated with **16a**–**c**, **17a**–**c**, and **24b**,**c**. Parasite proliferation was measured by SYBR-Safe assay of parasite DNA.^11,15^ The IC_50_ (concentration at which the compound suppresses parasite proliferation by 50%) was determined at various levels of extracellular thiamine (Table 3) and is compared to our previous data on oxythiamine **7a**.^15^

**Table 3:** IC_50_ Values (μM) for the Anti-plasmodial Activity, and Cytotoxicity of Selected Compounds

	**IC**_**50**_**of Suppression of*****P. falciparum*****proliferation (μM)**				
**Compound**	**Thiamine-free**	**[Thiamine] = 2.97 μM**	**[Thiamine] = 297 μM**	**HFF Cytotoxicity (μM)**	**Selectivity Index**^a^
**7a**^b^	11 ± 4	5200 ± 300	5500 ± 500	ND	ND
**16a**	>200	>200	>200	ND	ND
**16b**	>200	>200	>200	>200	ND
**16c**	67 ± 5	70 ± 8	89 ± 19	48 ± 5	0.7
**17a**	129 ± 9	156 ± 13	169 ± 11	>200	ND
**17b**	51 ± 3	78 ± 8	82 ± 11	90 ± 5	1.8
**17c**	44 ± 2	62 ± 7	69 ± 9	46 ± 7	1.1
**24b**	0.9 ± 0.1	1.3 ± 0.2	1.4 ± 0.4	15 ± 3	16.7
**24c**	3.0 ± 1.2	3.8 ± 2	4.0 ± 1.8	44 ± 4	14.7

a Selectivity index is IC_50_ (thiamine-free) vs HFF cytotoxicity; ND, not determined. Refer to Figures S8 and S9 for details.

b Ref (17).

Most of the thiamine analogues tested inhibited proliferation of the parasite (Table 3) in a dose-dependent manner (Figures 4A and S8). In thiamine-free culture medium, the PDHc-selective **16** and **17** (IC_50_ ≥ 44 μM) were considerably weaker than the multi-targeting hydroxamates **24b**,**c** (IC_50_ = 0.9–3 μM) and oxythiamine **7a** (IC_50_ = 11 μM). The comparable enzyme inhibition by **24b**,**c** and **7b** (Table 2) translated into similar low-micromolar anti-plasmodial activities (Table 3), though **24b** is 3-fold more active than **24c** and 11-fold more active than **7a**. This suggests that the passive diffusion of **24b**,**c** results in a greater concentration in the parasites than the presumed active transport and pyrophosphorylation of **7a**.

**Figure 4:**
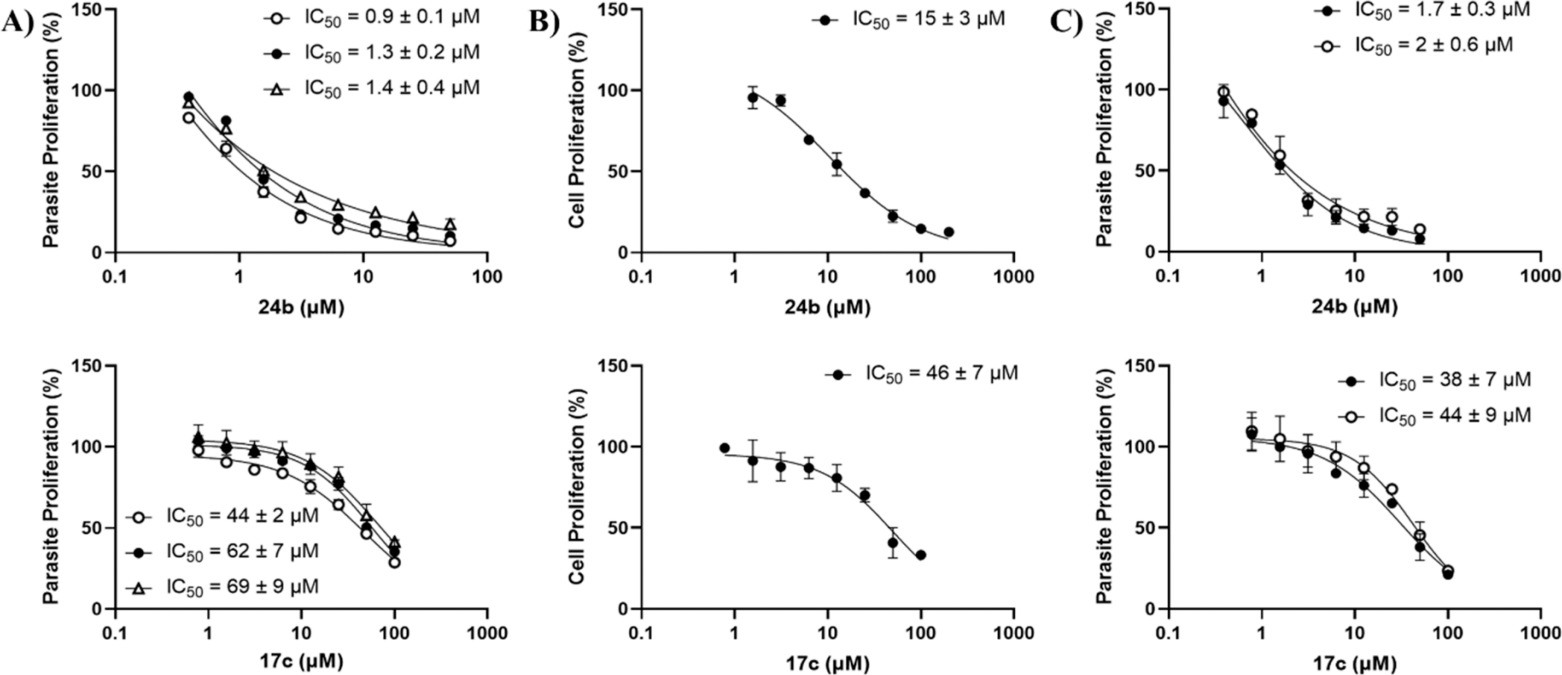
**Anti-plasmodial activities of 24b and 17c.** (**A**) Activities against *P*. *falciparum* 3D7 in medium containing thiamine at 0 μM (○), 2.97 (●), and 297 μM (△). (**B**) Cytotoxicities on HFF cells. (**C**) Activities against *P*. *falciparum* 3D7 transfected with an empty plasmid (●), and expressing *Pf*TPK-GFP (○) in thiamine-free medium. Data are the means of measurements from three independent experiments, each performed in three technical replicates.

With 2.97 μM thiamine in the culture medium (presumably leading to higher levels of ThDP within the parasites), the IC_50_ of the better performing hydroxamate **24b** increased from 0.9 ± 0.1 μM to 1.3 ± 0.2 μM (*n* = 3; *P* = 0.0188; *T*-test), and the IC_50_ of the best-performing bis-triazole **17c** increased from 44 ± 2 μM to 62 ± 7 μM (*n* = 3; *P* = 0.0136; *T*-test) (Figure 4A). This is consistent with their competitive relationship with thiamine/ThDP in cell-based assays. Interestingly, the drop in the inhibitory effect of **17c**, and **24b** was much less than that of oxythiamine **7a**.^15^ This suggests that the lowered inhibitory effect of oxythiamine is more due to competition with thiamine for transport into the cell and/or pyrophosphorylation by TPK (resulting in much less OxThDP being formed) than due to competition of OxThDP with ThDP for binding to the enzymes. Increasing the thiamine concentration in the culture medium 100-fold to 297 μM resulted in a statistically insignificant further change in the anti-plasmodial potency of all compounds (including **7a**) (Table 3). Probably one or more of the parasite’s thiamine-utilization steps become saturated even at 2.97 μM thiamine, so adding more extracellular thiamine no longer significantly increases the intracellular thiamine/ThDP levels.^17^

Some compounds were also tested on human foreskin fibroblast (HFF) cells. Most exhibited dose-dependent cytotoxicity (Table 3, and Figures 4B and S9), as expected (because thiamine is essential in all organisms). In the bis-triazole series, the longer derivatives (**16c** and **17c**) showed higher potencies than their shorter counterparts on both *P*. *falciparum* and human cells. Hydroxamate **24b** was the strongest inhibitor of proliferation of HFF cells, consistent with its potent inhibition of multiple ThDP-dependent enzymes. Although **7a** was not tested on HFF cells in this study, numerous *in vitro* and *in vivo* studies have confirmed that **7a** is toxic.^14−17^ Most of the compounds were non-selective, but **24b** and **24c** were 16.7 and 14.7 times more selective against the parasite than the HFF cells (Table 3).

Although **17c**, **24b** and **24c** are very similar in their inhibition of PDHc E1 (Figure 3A), **24b** is almost 50-fold more potent than **17c** at inhibiting proliferation of the parasites (Figure 4A). This suggests that some other ThDP-dependent enzyme(s) may be more critical than PDHc for proliferation. Possibilities include transketolase, which is required for synthesis of ribose 5-phosphate by the non-oxidative pentose phosphate pathway, and 1-deoxyxylulose 5-phosphate synthase, the first and rate-determining enzyme of terpene biosynthesis *via* the non-mevalonate pathway. Previous studies have shown that inhibition of the non-mevalonate pathway in *P*. *falciparum* does have an anti-proliferative effect.^29^ The greater activity on the parasites than on the HFF cells (Table 3) would be consistent with this, as human cells do not use the non-mevalonate pathway. The low anti-proliferative effect of **17c** might also be because PDHc and OGDHc enzymes can, to some extent, accept each other’s substrates, and so OGDHc could compensate for the lack of activity of the inhibited PDHc.^15^

We also tested **17c** and **24b** on a parasite line expressing extra copies of TPK (with a GFP-tag).^11^ Our previous studies showed that in the thiamine-free culture medium, the parasite over-expressing TPK was 70-fold more sensitive to oxythiamine **7a** than a parasite bearing an empty plasmid (the negative control).^15^ This is because the extra copies of TPK significantly improve the conversion of extracellular **7a** to intracellular **7b**.^11,15^ By contrast, in thiamine-free culture medium, the sensitivities of the two parasite lines to **17c** and to **24b** were almost identical (Figures 4C and S10, the small differences were statistically insignificant). This insensitivity toward TPK over-expression not only confirms that the analogues do not require the action of TPK for enzyme binding but also indicates that TPK is not their target.

These studies collectively suggest that hydroxamate **24b** is as competent as oxythiamine to cause thiamine deficiency in cells. However, with the triazole scaffold replacing the thiazolium ring, **24b** is uncharged under physiological conditions, so it is more drug-like^19,27,28^ and unlikely to be degraded by thiaminases.^16^ Moreover, without the diphosphate moiety, **24b** would likely retain activity on oxythiamine-resistant organisms that over-express the SLC19A1 transporter^14^ (which removes OxThDP from the cell) or ThDP hydrolases^23^ (which hydrolyze OxThDP to OxThMP). Also, the neutral triazole ring of **24b** is expected to improve the selectivity (relative to OxThDP) to the coenzyme role of ThDP over non-coenzyme roles. ThDP riboswitches are known to prefer a charged central ring,^22^ and the other (unknown) non-coenzyme targets probably do as well, as they are unlikely to be optimized for binding the neutral ThDP ylide **2b**, as the enzymes are. Furthermore, it most unlikely that **24b**, lacking the diphosphate, would be converted into derivatives equivalent to ThTP **3** or AThTP **4**, whereas OxThDP may well be.

Although the structural and mechanistic differences may make hydroxamate **24b** superior to oxythiamine **7a** in some ways, its utility in understanding the roles of the thiamine-utilization pathway in disease states is maximized when applied alongside **7a**, as in this study. We previously attributed the anti-plasmodial activity of oxythiamine to inhibition of multiple ThDP-dependent enzymes in the parasite,^15^ but it is possible that action on some non-coenzyme role of thiamine may have also contributed. In this study, however, **24b**, almost certainly lacking any activity on non-coenzyme roles, exhibited comparable activity to oxythiamine **7a** (in thiamine-free culture medium), which suggests that inhibition of ThDP-dependent enzymes is indeed the main target of oxythiamine. Thus, using **7a** and **24b** together helps assign the observed effects to the coenzyme or non-coenzyme role more confidently.

Bis-triazoles **17a**–**c** are potent and selective inhibitors of PDHc E1, which plays a vital role in bioenergetics, linking glycolysis with the Krebs cycle.^4^ The relative activities on cells (**17c** > **b** > **a**, Table 3) is likely to be a function of the PDHc E1 inhibition (**17c** > **b** > **a**, Table 2). It is, however, possible that the higher hydrophobicity of the longest derivative **17c** led to better cellular uptake. Further cell-based characterization of **17a**–**c** was not pursued due to their narrow-spectrum inhibition. However, recent studies have shown that selective PDHc E1 inhibitors have potential against certain cancers,^30,31^ so **17c** may be of interest in this field. The ligand efficiency of **17c** is good (0.34), so it could be a good starting point for the development of anti-cancer drugs.

In summary, this study has demonstrated hydroxamate **24b** is an antagonist of thiamine through inhibiting the coenzyme role of ThDP. Its utility is broad: active on both *P*. *falciparum* and human cells, and capable of inhibiting different ThDP-dependent enzymes from various species. We have discussed its use alongside oxythiamine **7a** in inducing thiamine deficiency in cell-based systems; this can help avoid misinterpretation of the role of these multi-targeting coenzyme antagonists and indicate whether coenzyme or non-coenzyme roles of thiamine are the main target. Based on its affinity and ligand efficiency on target enzymes as well as its potent and selective anti-plasmodial action, hydroxamate **24b** is a potential anti-malarial agent.

## Supporting Information Available

Supporting figures and tables, protocols for biological assays, computational studies, compound synthesis and characterization, and NMR spectra (PDF)

## Abbreviations

AThTP: adenosine thiamine triphosphate
HFF: human foreskin fibroblast
L.E.: ligand efficiency
MBG: metal-binding group
OGDHc E1: oxoglutarate dehydrogenase complex E1 subunit
OxThDP: oxythiamine diphosphate
PDC: pyruvate decarboxylase
PDHc E1: pyruvate dehydrogenase complex E1 subunit
PO: pyruvate oxidase
ThDP: thiamine diphosphate
ThTP: thiamine triphosphate
TK: transketolase
TPK: thiamine pyrophosphokinase
