# Supplementary material for: Inhibition of Thiamine Diphosphate-Dependent Enzymes by Triazole-Based Thiamine Analogues
Source: ACS Med Chem Lett. 2023 Apr 11;14(5):621–8. doi: 10.1021/acsmedchemlett.3c00047 (PMC10184313; doi:10.1021/acsmedchemlett.3c00047)
Supplement: Supplementary file 1 — ml3c00047_si_001.pdf [file ml3c00047_si_001.pdf]

Inhibition of Thiamine Diphosphate (ThDP)-dependent Enzymes by Triazole-based Thiamine Analogues

Alex H. Y. Chan,[a] Terence C. S. Ho,[a] Imam Fathoni,[b] Rebecca Pope,[a] Kevin J. Saliba,[b] and Finian J. Leeper[a,*]

[a] Yusuf Hamied Department of Chemistry, University of Cambridge, Lensfield Road, Cambridge CB2 1EW, UK

[b] Research School of Biology, The Australian National University, Canberra, ACT, 2601, Australia

[*]Corresponding author, e-mail address: fjl1@cam.ac.uk

Safety Statement

No unexpected or unusually high safety hazards were encountered in all described experiments.

Supplementary Information (SI)

Enzyme assays – Methods and Results **S1**

Ligand efficiencies – Calculations **S3**

Computational calculation of molecular properties – Methods and Results (Table S1) **S4**

Computational docking – Methods and Results (Figures S1-7) **S4**

Cell-based assays – Methods and Results (Figures S8-10) **S10**

Synthesis of compounds– General Methods and Experimental Procedures **S14**

NMR spectra **S27**

References **S70**

Enzyme assays (Methods and Results)

PDHc E1 inhibitory activity assay. Porcine PDHc E1 was purchased from Sigma. Its activity was determined by monitoring 2,6-dichlorophenolindophenol (DCPIP) reduction at 600 nm using a microplate reader (CLARIOstar) and conducted as described[1,2] with some modifications. The percentage inhibition of compounds against porcine PDHc E1 was assayed at a final concentration of 250 μM. The reaction buffer (50 mM KH_2PO_4 and 1 mM $MgCl_2$, pH 7) contained 50 μM ThDP (or 125 μM in the competition assay), 0.25 mM DCPIP, and 2 mg/ml porcine PDHc E1. The reaction mixture was preincubated at 37 °C for 30 min, then the reaction was initiated by adding pyruvate to a final concentration of 50 mM. To determine the half-maximal inhibitory concentration (IC_{50}), ThDP concentration was lowered to 10 μM, and inhibitor concentration was varied (0.4-200 μM). Specific activity was calculated using the molar extinction coefficient of DCPIP, 21 mM^{-1} cm^{-1}.[3] The enzyme IC_{50} values were calculated from non-linear regression curve fitting using GraphPad Prism. K_M(ThDP) was found to be 0.05 μM, consistent with the reported value.[4]

***S. cerevisiae* PDC inhibitory activity assay.** *S. cerevisiae* PDC was purchased from Sigma. Its activity was determined by monitoring DCPIP reduction at 600 nm using a microplate reader (CLARIOstar) and conducted as described[1,2] with some modifications. The percentage inhibition of compounds was assayed at a final concentration of 1500 μM. The reaction buffer (50 mM KH_2PO_4 and 1 mM $MgCl_2$, pH 7) contained 300 μM ThDP (or 750 μM in the competition assay), 0.27 mM DCPIP, and 0.15 mg/ml *S. cerevisiae* PDC. The reaction mixture was preincubated at 37 °C for 60 min, then reaction was initiated by adding pyruvate to a final concentration of 70 mM. Specific activity was calculated using the molar extinction coefficient of DCPIP, 21 mM^{-1} cm^{-1}.[3]

***E. coli* OGDHc E1 inhibitory activity assay.** *E. coli* OGDHc E1 was from our previous work[5] and had been donated by R. Frank. Its activity was determined by monitoring DCPIP reduction at 600 nm using a microplate reader (CLARIOstar) and conducted as described[1,2] with some modifications. The percentage inhibition of compounds against *E. coli* OGDHc E1 was assayed at a final concentration of 250 μM. The reaction buffer (50 mM KH_2PO_4 and 2 mM $MgCl_2$, pH 7) contained 50 μM ThDP (or 125 μM in the competition assay), 0.5 mM DCPIP, and 6.7 mg/ml *E. coli* OGDHc E1. The reaction mixture was preincubated at 37 °C for 60 min, then reaction was initiated by adding α-ketoglutarate to a final concentration of 10 mM. To determine the IC_{50}, ThDP concentration was lowered to 30 μM, and inhibitor concentration was varied (2-1000 μM). Specific activity was calculated using the molar extinction coefficient of DCPIP, 21 mM^{-1} cm^{-1}.[3] The enzyme IC_{50} values were calculated as described for the PDHc E1 assay. K_M(ThDP) was found to be 3 μM, consistent with the reported value.[5]

***A. viridans* PO inhibitory activity assay.** *A. viridans* PO and horseradish peroxidase were purchased from Sigma. *A. viridans* PO activity was determined by monitoring appearance of quinoneimine dye at 550 nm using a microplate reader (CLARIOstar) and conducted as described[1] with some modifications. The percentage inhibition of compounds against *A. viridans* PO was assayed at a final concentration of 250 μM. The reaction buffer (50 mM KH_2PO_4 and 10 mM $MgCl_2$, pH 5.9) contained 50 μM ThDP (or 125 μM in the competition assay), 10 μM flavin adenine dinucleotide (FAD), 0.15% 4-Aminoantipyrine, 0.3% N-Ethyl-N-(2-hydroxy-3-sulfopropyl)-m-toluidine (EHSPT), 50 μg/mL horseradish peroxidase and 0.35 U/mL *A. viridans* PO. The reaction mixture was preincubated at 37 °C for 30 min, then reaction was initiated by adding pyruvate to a final concentration of 50 mM. To determine the IC_{50}, inhibitor concentration was varied (2-1000 μM) with ThDP concentration at 50 μM. 1 unit of PO activity is defined as 1 μmol of hydrogen peroxide produced per minute. The enzyme IC_{50} values were calculated as described for the PDHc E1 assay. K_M(ThDP) was found to be 5 μM.

***Z. mobilis* PDC inhibitory activity assay.** *Z. mobilis* PDC was expressed and purified following a reported method.[6] *Z. mobilis* PDC activity was determined by monitoring reduced nicotinamide adenine dinucleotide (NADH) consumption at 340 nm using a microplate reader (CLARIOstar) and conducted as described[6] with some modifications. The reaction buffer (50 mM MES-KOH and 5 mM $MgCl_2$, pH 6.5) contained 10 μM ThDP, 150 μM NADH, 10 U/ml alcohol dehydrogenase (ADH) and 0.5 μM of active sites of *Z. mobilis* PDC. To determine the IC_{50}, inhibitor concentration was varied (0.4-200 μM). The reaction mixture was preincubated at 37 °C for 60 min, then reaction was initiated by adding pyruvate to a final concentration of 10 mM. The enzyme IC_{50} values were calculated as described for the PDHc E1 assay. K_M(ThDP) was found to be 0.35 μM, consistent with the reported values.[5,6]

Ligand efficiencies (Calculations)

L.E. measures the binding energy of a ligand to its target (in kcal mol^{-1}) per heavy atom of the ligand.

Compound **17c**: ($C_{21}H_{25}N_9O$; Heavy atoms: 31)

PDHc E1: $\Delta G = -RT \times \ln(K_I)$, thus $\Delta G = -(8.314)(310) \times \ln(0.03\ \mu M)$;

$\Delta G = 44645$ J mol^{-1} or 10.67 kcal mol^{-1}

L.E. = 10.67 / 31 = 0.344

Compound **24b**: ($C_{12}H_{17}N_7O_2$; Heavy atoms: 21)

PDHc E1: $\Delta G = -RT \times \ln(K_I)$, thus $\Delta G = -(8.314)(310) \times \ln(0.045\ \mu M)$;

$\Delta G = 43600$ J mol^{-1} or 10.42 kcal mol^{-1}

L.E. = 10.42 / 21 = 0.496

PDC: $\Delta G = -RT \times \ln(K_I)$, thus $\Delta G = -(8.314)(310) \times \ln(0.68\ \mu M)$;

$\Delta G = 36601$ J mol^{-1} or 8.75 kcal mol^{-1}

L.E. = 8.75 / 21 = 0.417

OGDHc E1: $\Delta G = -RT \times \ln(K_I)$, thus $\Delta G = -(8.314)(310) \times \ln(2.02\ \mu M)$;

$\Delta G = 33795$ J mol^{-1} or 8.08 kcal mol^{-1}

L.E. = 8.08 / 21 = 0.384

PO: $\Delta G = -RT \times \ln(K_I)$, thus $\Delta G = -(8.314)(310) \times \ln(20.2\ \mu M)$;

$\Delta G = 27861$ J mol^{-1} or 6.66 kcal mol^{-1}

L.E. = 6.66 / 21 = 0.317

Compound **24c**: ($C_{13}H_{19}N_7O_2$; Heavy atoms: 22)

PDHc E1: $\Delta G = -RT \times \ln(K_I)$, thus $\Delta G = -(8.314)(310) \times \ln(0.040\ \mu M)$;

$\Delta G = 43903$ J mol^{-1} or 10.49 kcal mol^{-1}

L.E. = 10.49 / 22 = 0.477

PDC: $\Delta G = -RT \times \ln(K_I)$, thus $\Delta G = -(8.314)(310) \times \ln(0.89\ \mu M)$;

$\Delta G = 35908$ J mol^{-1} or 8.58 kcal mol^{-1}

L.E. = 8.58 / 22 = 0.390

OGDHc E1: $\Delta G = -RT \times \ln(K_I)$, thus $\Delta G = -(8.314)(310) \times \ln(1.46\ \mu M)$;

$\Delta G = 34632$ J mol^{-1} or 8.28 kcal mol^{-1}

L.E. = 8.28 / 22 = 0.376

PO: $\Delta G = -RT \times \ln(K_I)$, thus $\Delta G = -(8.314)(310) \times \ln(23.2\ \mu M)$;

$\Delta G = 27504$ J mol^{-1} or 6.57 kcal mol^{-1}

L.E. = 6.66 / 22 = 0.299

Computational calculation of molecular properties (Methods and Results)

Many studies have provided guidelines[7] for drug design to predict the oral bioavailability of compounds:

- Molecular weight (MW): ≤ 400
- Log P: ≤ 4
- HB donors (HBDs, *i.e.* no. of N-H and O-H bonds): ≤ 5
- HB acceptors (HBAs, *i.e.* no. of N and O atoms): ≤ 10
- Total polar surface area (TPSA) at pH = 7.4: ≤ 140 $Å^2$
- Rotatable bonds (RBs): ≤ 10-12

Table S1. Physicochemical properties of key compounds bis-triazole 17c and hydroxamates 24b and c (calculated using MarvinSketch 21.2).

Physicochemical properties	**Bis-triazole 17c**	**Hydroxamate 24b**	**Hydroxamate 24c**
MW	419	291	305
cLogP	2.5	-0.5	-0.1
HBDs	2	4	4
HBAs	10	9	9
TPSA ($Å^2$)	123	132	132
RBs	9	6	7

Computational docking (Methods and Results)

Docking of ThDP and compounds were executed using CCDC GOLD docking program with PDB: 6CFO, 1PVD, 1V5F and 6U3J for human PDHc E1, *S. cerevisiae* PDC, *A. viridans* PO and human OGDHc E1, respectively. The binding site of ThDP was selected as the docking site. Our molecules were generated using Mercury. GA runs were set at 20 and was user-defined with population size of 200 and 200000 number of operations. No early termination was permitted. Similarity and scaffold constraint to the original ligand were implemented on our compounds to mimic their binding positions. CHEMPLP and GoldScore were the docking scoring and rescoring respectively.[8] Interactions between docked compounds and protein models are shown using CCDC GOLD.

*Compound series **13a,14a,19a,20a**:*

No molecular docking was performed. The non-metal binding cyano-derivatives (**14a** and **20a**) were included as the negative controls for the corresponding trifluoromethyl-1,2,4-oxadiazole derivatives (**13a** and **19a**, respectively), but the lack of significant differences in inhibition (%) with both the **13a-14a** and **19a-20a** pairs implies that the trifluoromethyl-1,2,4-oxadiazole motif was not binding to Mg^{2+}.

*Compound series **21a-c** and **22a-c**:*

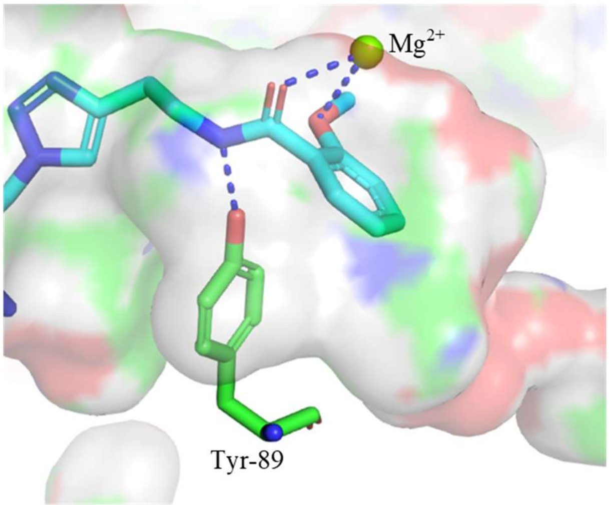

Figure S1. Docking of **22a** into the ThDP pocket of human PDHc E1 with interactions shown; Mg^{2+} shown as yellow-green sphere. Both oxygen atoms were predicted to interact with the Mg^{2+} in a six-membered cyclic form. Only one oxygen atom was predicted to interact with the Mg^{2+} with **21a-c** and **22b-c** (not shown); this may explain their weaker binding. The interactions of the pyrimidine ring are not shown as they are conserved.

Similar binding modes are also present in the docking models of **22a** in PDC, OGDHc E1 and PO, thus potentially explaining its inhibitory activities on PDC and OGDHc E1. Interestingly, **22a** was found to be inactive on PO. As most of our triazole thiamine derivatives were either inactive or weakly active on PO regardless of the MBG identity, we suggest that the intrinsic affinity of the aminopyrimidine-CH_2-triazole motif is low (relative to other three enzymes) and the affinity of the diphosphate is relatively more important. Therefore, the above binding mode is presumably present in the **22a**-PO complex, but, at the concentration tested ([inhibitor]:[ThDP] = 5:1), **22a** was not potent enough to out-compete ThDP.

*Compound series **18a-c**:*

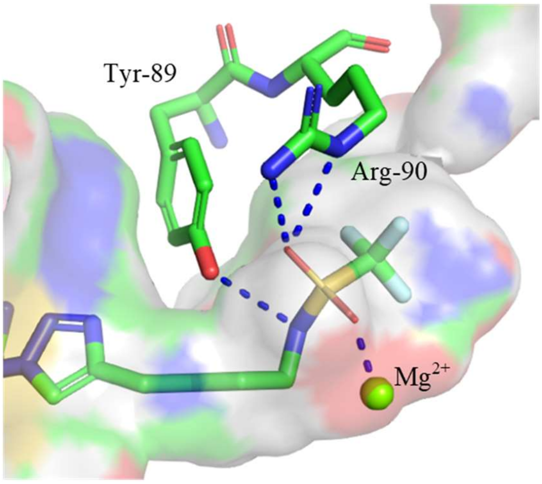

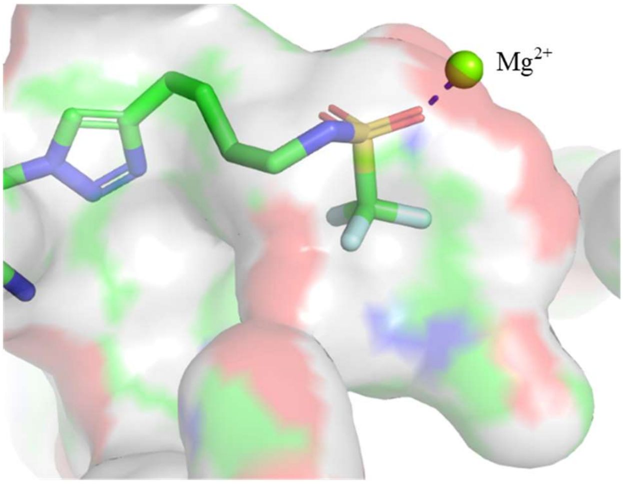

Figure S2. Docking of **18c** into the ThDP pockets as surface of human PDHc E1 (left) and human OGDHc E1 (right) with interactions shown; Mg^{2+} shown as yellow-green sphere. Instead of the (possibly deprotonated) nitrogen atom, an oxygen of a S=O bond was predicted to interact with the Mg^{2+}; the sulphonamide moiety may pick up some random interactions in the diphosphate pocket. The linkers of **18b** and **18c** appeared not to be optimal to position a O atom for interaction with the Mg^{2+} (not shown), and this may explain their lower affinities. The interactions of the aminopyrimidine are not shown as they are conserved.

*Compound series **26a-c**:*

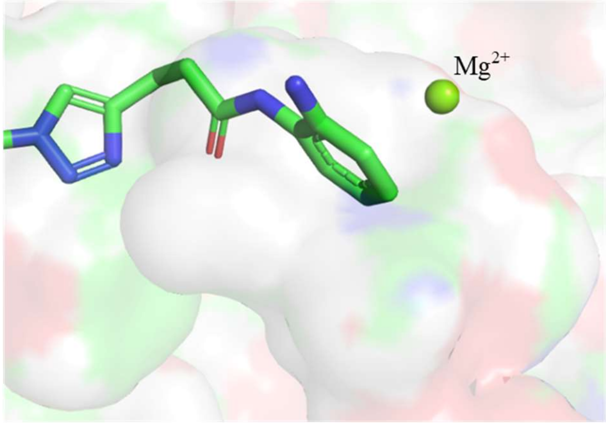

Figure S3. Docking of **26a** into the ThDP-pocket of human PDHc E1, shown as surface; Mg^{2+} shown as yellow-green sphere. The aromatic ring was predicted to form a cation-π interaction with Mg^{2+} while the carbonyl oxygen and the benzamide amino group engage in polar interactions with the surrounding residues. These geometry sensitive interactions were distorted (not shown) with the longer **26b** and **26c**, and this may explain their lower affinities. The interactions of the aminopyrimidine are not shown as they are conserved.

*Oxythiamine diphosphate (OxThDP) 7**b**:*

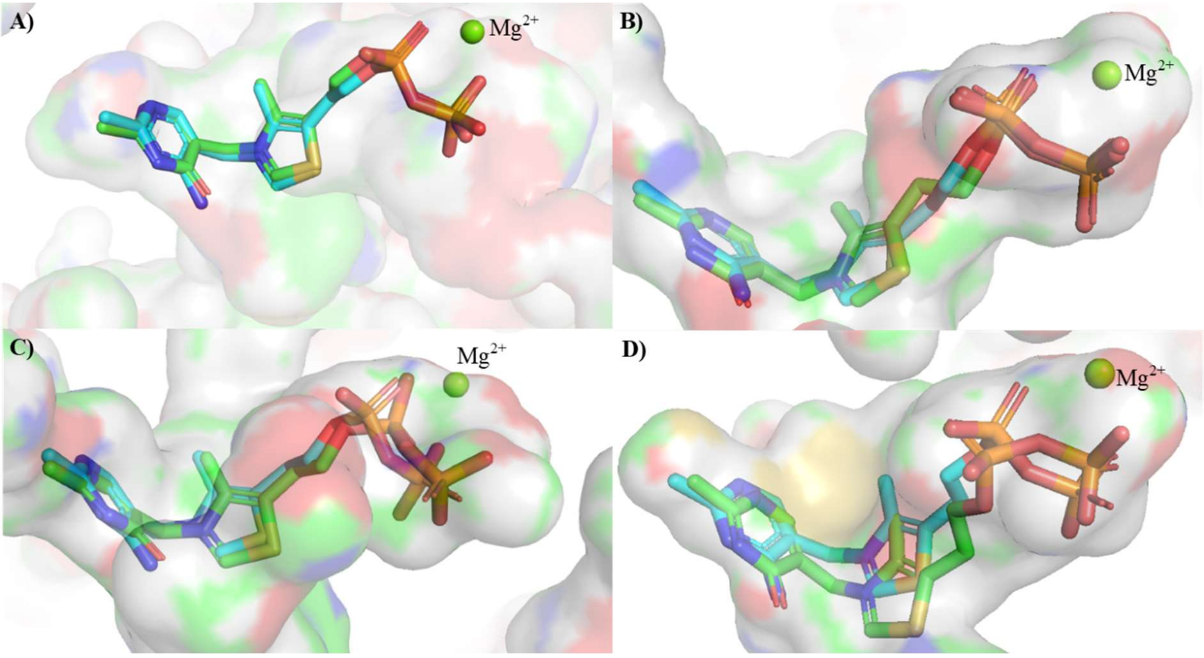

Figure S4. Docking of OxThDP **7b** (yellow carbons) into the ThDP pocket of human PDHc E1 (**A**), *S. cerevisiae* PDC (**B**), human OGDHc E1 (**C**) and *A. viridans* PO (**D**), overlayed with ThDP (cyan carbons); ThDP pocket and Mg^{2+} are shown as surface and yellow-green sphere.

*Compound series **24a-c**:*

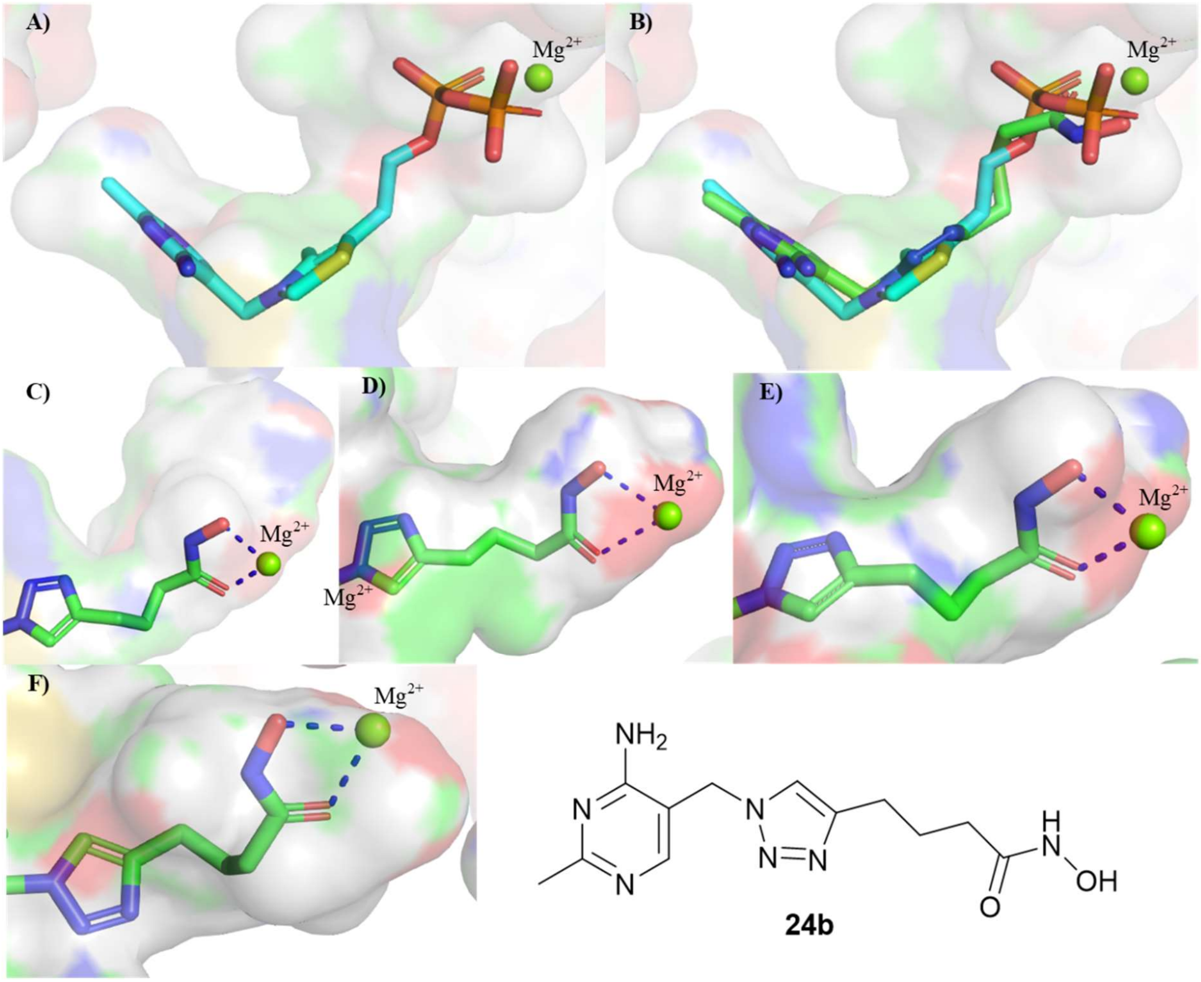

Figure S5. **Docking of 24b to the ThDP-binding site of four enzymes**. (**A**) Binding mode of ThDP (cyan carbons) in human PDHc E1 showing the V-shaped conformation between the aminopyrimidine and the thiazolium ring; the ThDP pocket is shown as surface; (**B**) Binding mode of **24b** (yellow carbons) in human PDHc E1 from molecular docking, overlayed with ThDP (cyan carbons) as in (**A**). (**C**)-(**F**) Molecular docking predicted the expected bidentate binding mode of **24b** to human PDHc E1 (**C**), *S. cerevisiae* PDC (**D**), human OGDHc E1 (**E**) and *A. viridans* PO (**F**); in each case the ThDP-pocket is shown as surface and Mg^{2+} is shown as a yellow-green sphere. Collectively, these docking models support the ThDP-competitive nature of **24b** which occupied the ThDP pockets with bidentate binding to the Mg^{2+} (as summarised in Figure 2). In **C**, **D**, **E** and **F**, the interactions of the aminopyrimidine are not shown as they are conserved.

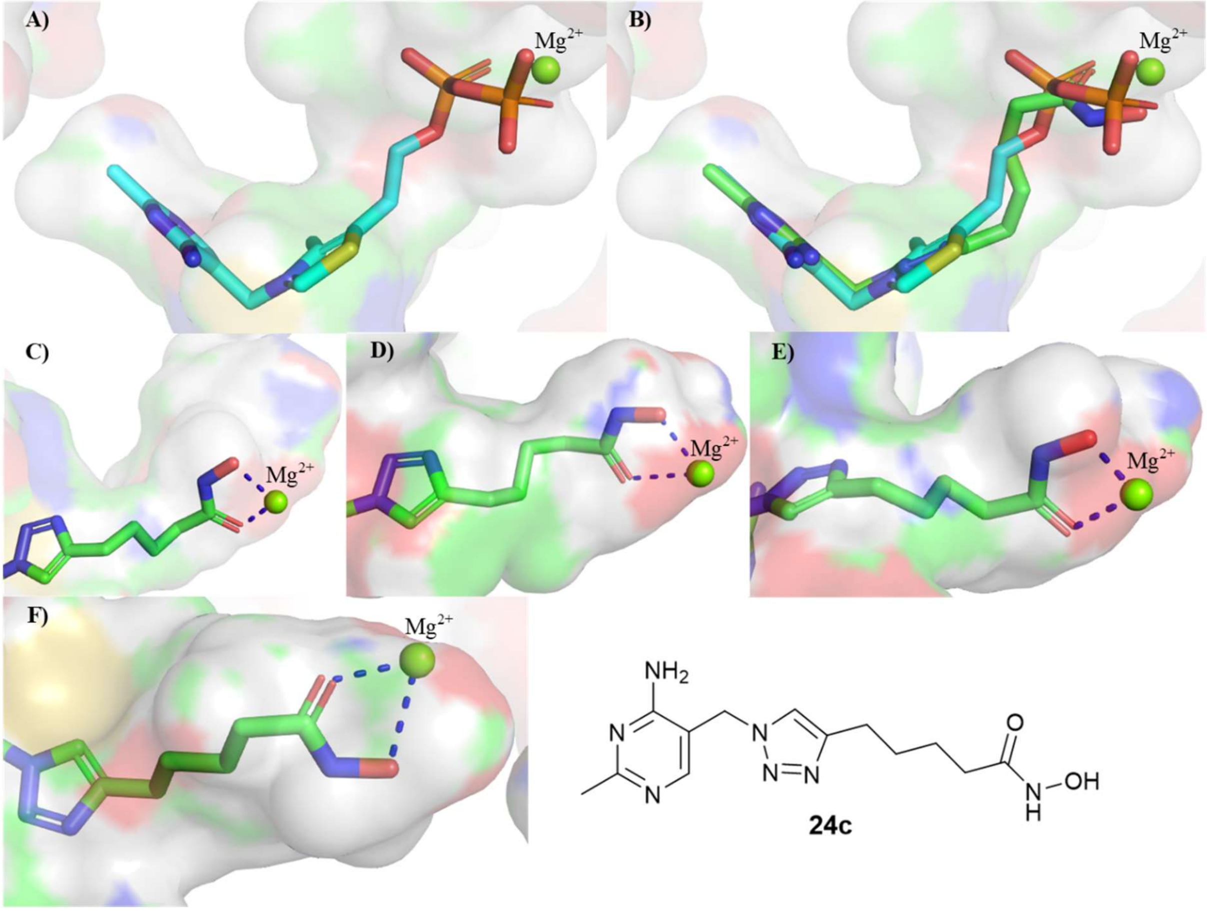

Figure S6. **Binding of 24c to four enzymes**. (**A**) Binding mode of ThDP (cyan carbons) in human PDHc E1 showing the V-shaped conformation between the aminopyrimidine and the thiazolium ring; the ThDP pocket is shown as surface; (**B**) Binding of **24c** (grenn carbons) to human PDHc E1 from molecular docking, overlayed with ThDP (cyan carbons) as in (**A**). (**C**)-(**F**) Molecular docking predicted the expected bidentate binding mode of **24c** to the Mg^{2+} in human PDHc E1 (**C**), *S. cerevisiae* PDC (**D**), human OGDHc E1 (**E**) and *A. viridans* PO (**F**); in each case the ThDP-pocket is shown as surface and Mg^{2+} is shown as a yellow-green sphere. Collectively, these docking models support the ThDP-competitive nature of **24b** which occupied the ThDP pockets with bidentate binding to the Mg^{2+} (as summarised in Figure 2). In **C**, **D**, **E** and **F**, the interactions of the aminopyrimidine are not shown as they are conserved.

Compound series ***15a-c, 16a-c*** *and* ***17a-c****:*

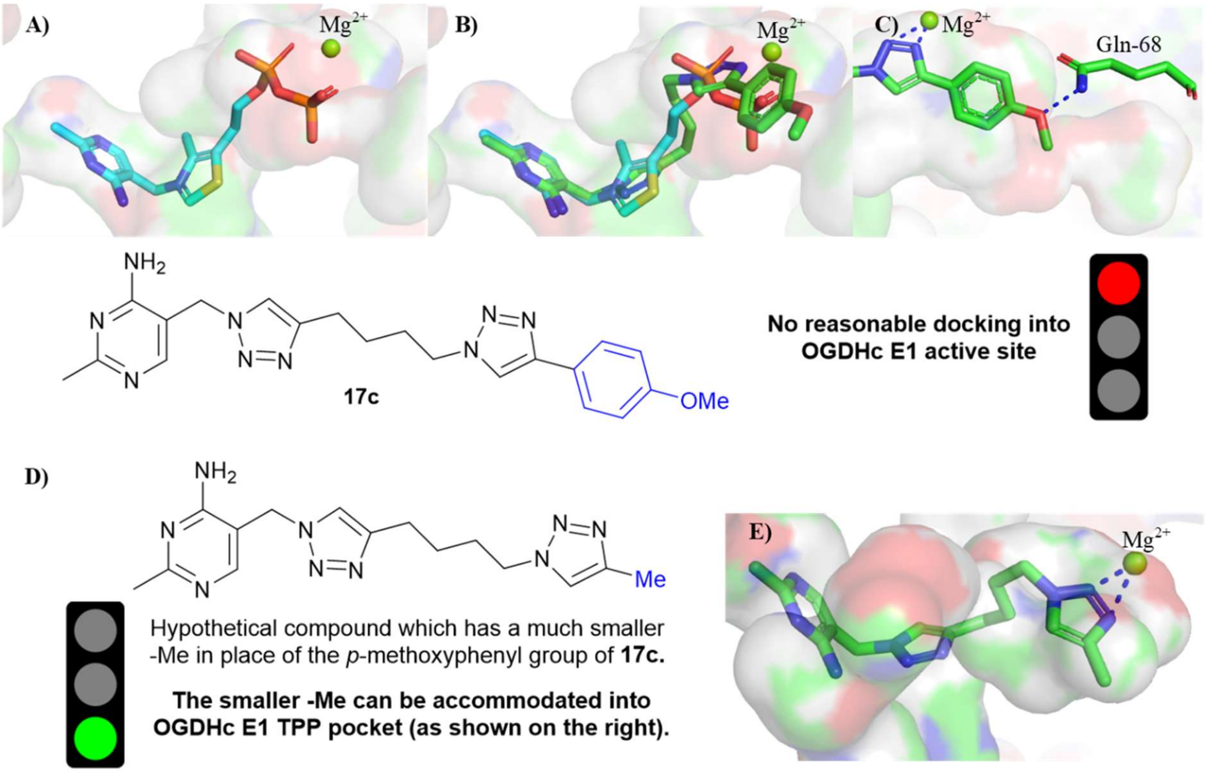

Figure S7. **Binding of 17c to human PDHc E1.** (A) Binding mode of ThDP (cyan carbons) in human PDHc E1 ThDP-pocket (shown as surface) showing the V-shaped conformation between the aminopyrimidine and the thiazolium ring; (**B**) Predicted binding mode of **17c** (yellow carbons) in human PDHc E1 from molecular docking overlayed with ThDP (cyan carbons) as in (**A**); (**C**) The predicted binding mode of **17c** in the human PDHc E1 ThDP-pocket (shown as surface); the triazole nitrogen atoms interact with Mg^{2+} as expected; (**D**) Structure of a hypothetical compound in which the much bigger *p*-methoxyphenyl substituent on the terminal triazole ring of **17c** is replaced by a smaller methyl group; (**E**) Predicted binding mode of the hypothetical compound in human OGDHc E1 ThDP-pocket.

Docking studies of **17c** with the PDC and PO enzymes (not shown) gave similar outcomes to the above with human OGDHc E1, suggesting that the lack of activity of **17c** on all three enzymes is due to a steric clash between the diphosphate-binding pocket and the bulky *p*-methoxyphenyl terminus of **17c**.

Cell-based assays (Results)

Anti-plasmodial activity assay. This study used human malaria parasite *P. falciparum* strain 3D7 (chloroquine-sensitive) and the same strain expressing an extra copy of TPK with a GFP-tag (*Pf*TPK-GFP) generated as previously described.[9] The intraerythrocytic stage of the parasites were maintained essentially as previously described.[10] Compounds were tested at concentrations up to a highest final concentration depending on their solubility (between 25 µM and 200 µM). Compound stock solutions were prepared in dimethyl sulfoxide (DMSO) followed by dilution in RPMI 1640 medium in the absence of thiamine or in the presence of 2.97 µM (or 297 µM) thiamine. The final concentration of DMSO that the parasites were exposed to never exceeded 0.05%. Two-fold serial dilutions were then performed, with each concentration tested in triplicate. The assay was performed as described[11] with some modifications. Experiments were initiated with parasites in the ring-stage, a parasitemia level of 0.5% and a haematocrit of 2%. Chloroquine (0.5 µM) was used as the positive control (*i.e.* complete inhibition of parasite proliferation), and parasites maintained in the absence of any inhibitor represented 100% parasite proliferation. The final volume in each well was 200 µL. Plates were incubated at 37 °C, under an atmosphere of 96% nitrogen, 3% carbon dioxide and 1% oxygen. Parasite proliferation was measured by performing SYBR-Safe assay.[12] The concentration at which the compound suppresses parasite proliferation by 50% (*i.e.* parasite IC_{50}) was determined from data calculated using Microsoft Excel and analysed by non-linear regression plot using GraphPad Prism. The data were averaged from three independent experiments.

Cytotoxicity assay. This study used HFF cells (human foreskin fibroblasts) as described[13] with some modifications. The HFF cells were seeded in 96-well plates at a density of about 13 x 10^4 cells/mL. Cycloheximide (10 µM) was used as a control to indicate complete inhibition of HFF cell proliferation. Plates were incubated at 37 °C in a humidified 5% carbon dioxide incubator for 96 h. A sample of the supernatant (150 µL) was then carefully aspirated from each well and discarded. The plates were then stored at -80 °C. SYBR- Safe assay was used. The plates were thawed, SYBR-Safe lysis solution (150 µL) was added to each well and mixed via pipetting to ensure the HFF cells were detached from the plate and lysed. The plates were then processed as described for the anti-plasmodial assay.

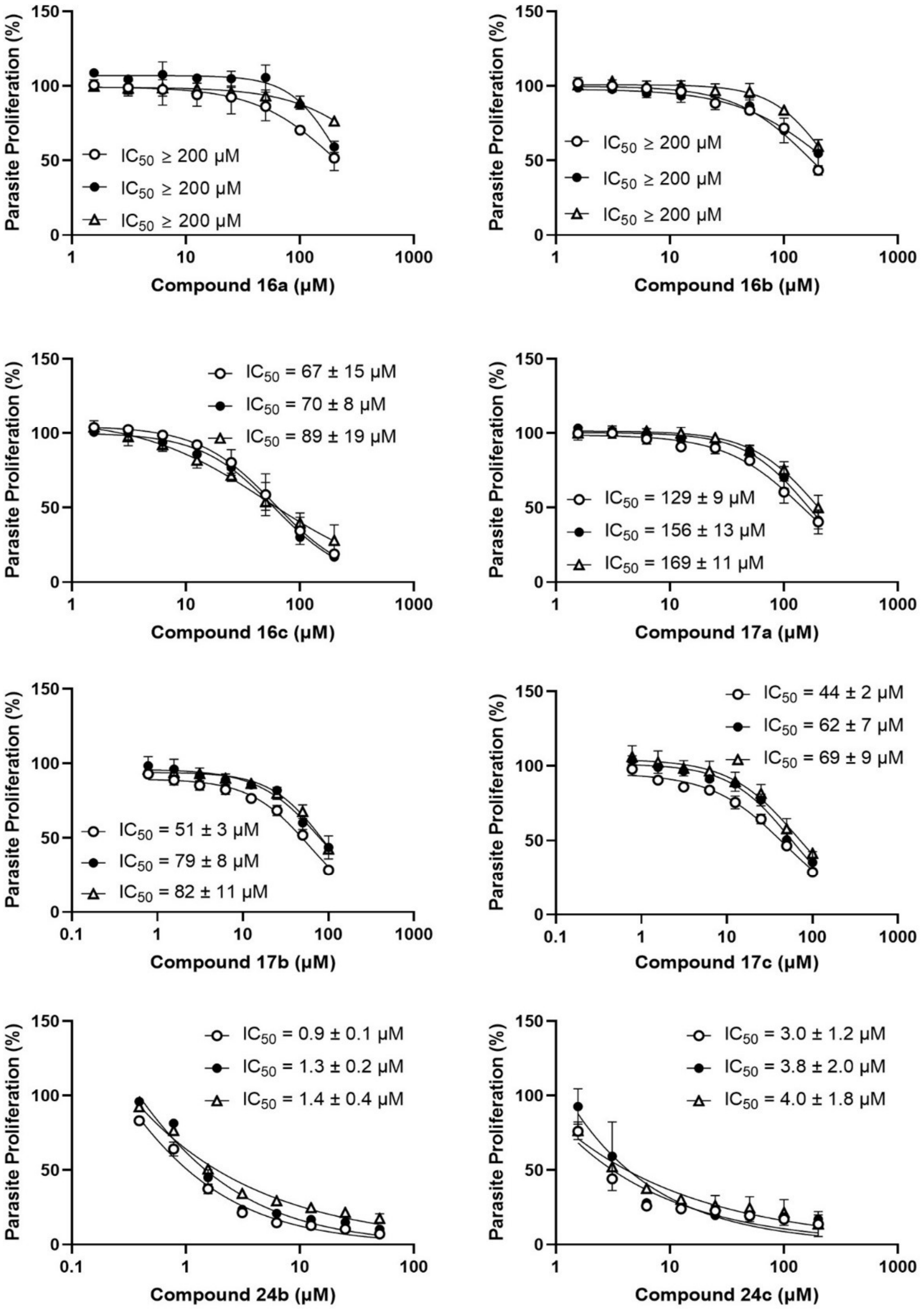

Figure S8. *In vitro* anti-plasmodial activity of the compounds against 3D7 parasites in thiamine-free medium (white circles), medium with 2.97 μM thiamine (black circles), and 297 μM thiamine (white triangles) under assay conditions as described above. Data are averaged from three independent experiments, each carried out in triplicate. Error bars represent SEM and, where not visible, are smaller than the symbols. Data presented for compounds **17c** and **24b** are shown in Figure 4 in the main text but are included here for completeness.

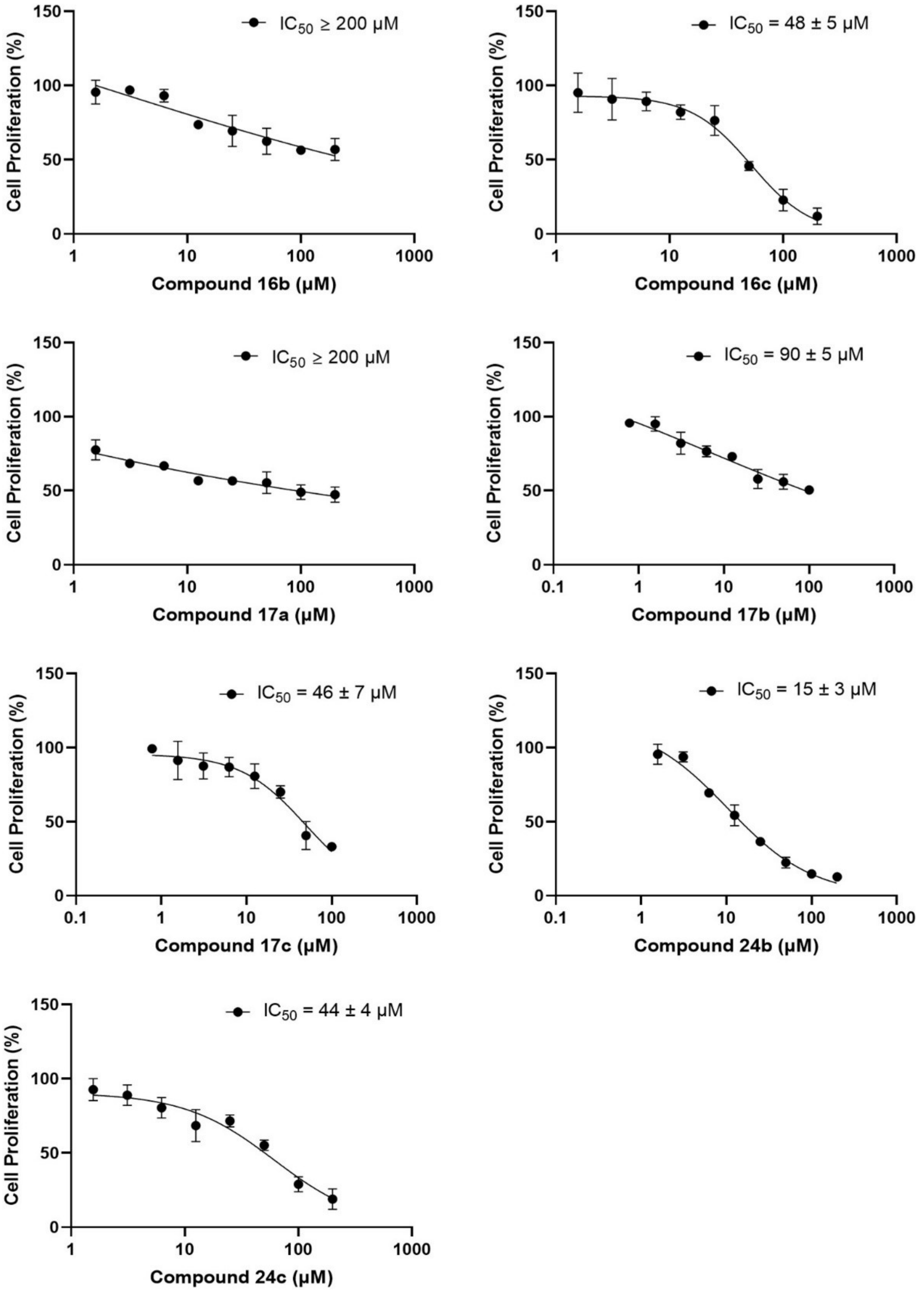

Figure S9. *In vitro* cytotoxicity result of the compounds against HFF cells. Data are averaged from three independent experiments, each carried out in triplicate. Error bars represent SEM and, where not visible, are smaller than the symbols. Data presented for compounds **17c** and **24b** are shown in Figure 4 in the main text but are included here for completeness.

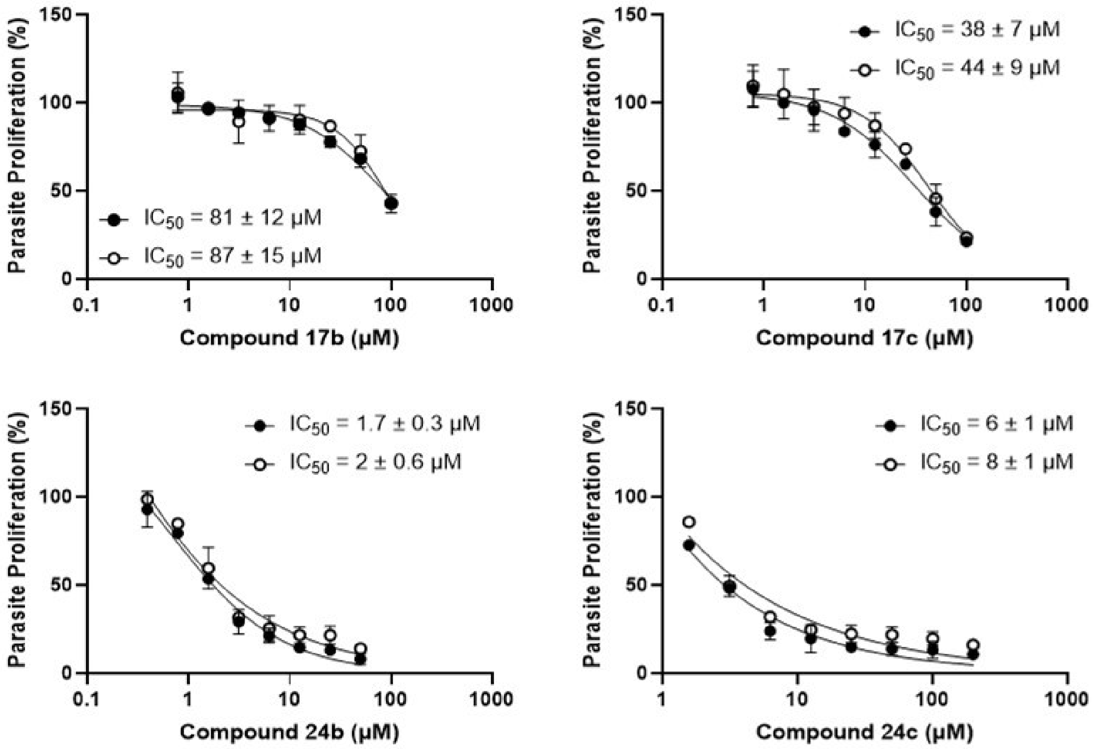

Figure S10. *In vitro* anti-plasmodial activity of compounds **17b**,**c** and **24b**,**c** against P. *falciparum* 3D7 parasites transfected with an empty plasmid (black circles), and 3D7 parasites expressing *Pf*TPK-GFP (white circles) in the thiamine-free medium. Data are averaged from three independent experiments, each carried out in triplicate. Error bars represent SEM and, where not visible, are smaller than the symbols. Data presented for compounds **17c** and **24b** are shown in Figure 4 in the main text but are included here for completeness.

General synthesis (Methods)

Oxygen- and moisture-sensitive reactions were carried out in flame-dried glassware under a nitrogen atmosphere. Unless otherwise stated, all chemicals and reagents were purchased from commercial suppliers and used without further purification. Reaction progress was monitored by analytical thin-layer chromatography (TLC). TLC was conducted using Merck glass plates with silica Kieselgel 60 F254 of thickness 0.25 mm and visualised under 254 nm UV lamp or potassium permanganate staining solution (with light heating). Flash column chromatography was carried out in the indicated solvent system using prepacked silica gel cartridges for use on the Biotage Purification System. All solvents were removed under reduced pressure using a Büchi rotary evaporator with dry ice traps.

All yields refer to chromatographically and spectroscopically pure compounds unless otherwise stated. Known compounds were characterised by, at minimum, ^{1}H NMR spectroscopy. New synthetic intermediates were characterised by, at minimum, ^{1}H NMR spectroscopy, ^{13}C NMR spectroscopy (except **10a-c**) and ESI-MS unless otherwise stated. Compounds subjected to biological assays were characterised by, at minimum, ^{1}H NMR spectroscopy, ^{13}C NMR spectroscopy and HRMS.

^{1}H NMR spectra were recorded at 400 MHz or 700 MHz in $CDCl_3$, CD_3OD or CD_3SOCD_3 solution on a Bruker 400 MHz or 700MHz spectrometer and chemical shifts were recorded in parts per million (ppm). ^{13}C NMR spectra were recorded on either a Bruker 400 MHz or 700 MHz spectrometer. ^{19}F NMR spectra were recorded on either a Bruker 400 MHz or 700 MHz spectrometer. Resonances are described using the following abbreviations: s (singlet), d (doublet), t (triplet), q (quartet), quin. (quintet), m (multiplet), br (broad), dd (doublet of doublets), etc. Coupling constants (*J*) are given in Hz and are rounded to the nearest 0.1 Hz. All NMR data were collected at 25 °C. Mass spectra used electrospray ionisation (ESI). Melting points of compounds were measured using a Reichert apparatus and are uncorrected.

Experimental procedures (Synthesis)

Preparation of the common precursor – 5-(azidomethyl)-2-methylpyrimidin-4-amine 8

To a stirred solution of thiamine hydrochloride (10 g, 30 mmol) and NaN_3 (5 g, 77 mmol) in water (100 mL, 0.3 M) was added Na_2SO_3 (0.4 g, 3.2 mmol). The resultant mixture was stirred at 70 °C overnight, then acidified with citric acid to pH 4-5, washed with DCM (200 mL), and basified with potassium carbonate to pH 8-10. Upon product precipitation, the suspension was cooled in an ice bath and filtered under reduced pressure. The residue was rinsed with cold water and dried under reduced pressure to yield azide **8** as a white solid. Typical yields: 1.5-2.5 g (31-51%). ^{1}H NMR (400 MHz, CD_3OD) δ 8.02 (s, 1H), 4.32 (s, 2H), 2.43 (s, 3H). ^{13}C NMR (100 MHz, CD_3OD) δ 167.2, 162.3, 154.2, 108.7, 47.8, 23.6. Analytical data are consistent with those previously reported.[14]

To increase the yield, the filtrate was extracted with EtOAc (100 mL x 2). The combined organic phases were dried over $MgSO_4$, filtered, and evaporated under reduced pressure. The solid residues were pooled with the precipitate (collected upon filtration) and recrystallised from EtOAc-hexane and dried under reduced pressure to yield the product as a white solid (3.6 g, 75%).

General procedure for preparation of 9a-c:

To a stirred solution of corresponding alkynol (10 mmol, 1 equiv.) and azide **8** (1.2 equiv.) in *t*-BuOH and water (2:1, 0.2 M) was added $CuSO_4.5H_2O$ (0.03 equiv.) and sodium ascorbate (0.3 equiv.). The resultant mixture was stirred at r.t. for 2 days, then concentrated under reduced pressure, diluted with *n*-BuOH, washed with 0.1 M K_2CO_3, dried over $MgSO_4$, filtered, and evaporated under reduced pressure. The residue was purified by silica flash chromatography (10% MeOH in DCM) to yield triazole **9a-c** as a solid.

2-{1-[(4-Amino-2-methylpyrimidin-5-yl)methyl]-1H-1,2,3-triazol-4-yl}ethan-1-ol **9a**

Prepared from 3-butyn-1-ol. White solid (1.64 g, 70%). ^{1}H NMR (400 MHz, CD_3OD) δ 8.06 (s, 1H), 7.83 (s, 1H), 5.47 (s, 2H), 3.80 (t, 2H, J = 6.5 Hz), 2.90 (t, 2H, J = 6.5 Hz), 2.42 (s, 3H). Analytical data are consistent with those previously reported.[14]

3-{1-[(4-Amino-2-methylpyrimidin-5-yl)methyl]-1H-1,2,3-triazol-4-yl}propan-1-ol **9b**

Prepared from 4-pentyn-1-ol. White solid (1.81 g, 73%). m.p. 168-169 °C. ^{1}H NMR (400 MHz, CD_3OD) δ 8.05 (s, 1H), 7.79 (s, 1H), 5.48 (s, 2H), 3.59 (t, 2H, J = 6.4 Hz), 2.78 (t, 2H, J = 7.6 Hz), 2.43 (s, 3H), 1.88 (m, 2H). ^{13}C NMR (100 MHz, CD_3OD) δ 167.5, 162.1, 155.0, 147.9, 121.9, 108.6, 60.6, 47.2, 31.8, 23.7, 21.3. HRMS (ESI) m/z: [M+H$^+$] calculated for $C_{11}H_{16}N_6O$: 249.1463; found: 249.1458.

4-{1-[(4-Amino-2-methylpyrimidin-5-yl)methyl]-1H-1,2,3-triazol-4-yl}butan-1-ol **9c**

Prepared from 5-hexyn-1-ol. White solid (1.90 g, 72%). m.p. 171-172 °C. ^{1}H NMR (400 MHz, CD_3OD) δ 8.04 (s, 1H), 7.79 (s, 1H), 5.47 (s, 2H), 3.57 (t, 2H, J = 6.4 Hz), 2.72 (t, 2H, J = 7.7 Hz), 2.43 (s, 3H), 1.73 (m, 2H), 1.57 (m, 2H). ^{13}C NMR (100 MHz, CD_3OD) δ 167.5, 162.2, 155.0, 148.3, 121.9, 108.5, 61.2, 47.2, 31.6, 25.4, 24.6, 23.6. HRMS (ESI) m/z: [M+H$^+$] calculated for $C_{12}H_{18}N_6O$: 263.1620; found: 263.1622.

General procedure for preparation of 10a-c:

To a stirred suspension of alcohol **9a-c** (5 mmol, 1 equiv.) in dry pyridine (0.2 M) under nitrogen at 0 °C was added *p*-TsCl (5 equiv.) in three portions. The resultant mixture was stirred at r.t. for 4 h, then quenched with cold 1 M HCl, diluted with water, neutralised with sodium bicarbonate to pH 7, and extracted with DCM. The organic phase was washed with sat. aq. Cu_2SO_4, dried over $MgSO_4$, filtered, and evaporated under reduced pressure. The residue was purified by silica flash chromatography (10% MeOH in DCM) to yield tosylate **10a-c** as a solid.

2-{1-[(4-Amino-2-methylpyrimidin-5-yl)methyl]-1H-1,2,3-triazol-4-yl}ethyl 4-methylbenzene-1-sulfonate **10a**

White solid (1.6 g, 82%). ^{1}H NMR (400 MHz, CD_3OD) δ 8.07 (s, 1H), 7.79 (s, 1H), 7.67 (d, 2H, J = 8.1 Hz), 7.36 (d, 2H, J = 8.1 Hz), 5.45 (s, 2H), 4.25 (t, 2H, J = 6.3 Hz), 3.03 (t, 2H, J = 6.3 Hz), 2.42 (s, 3H), 2.41 (s, 3H). Analytical data are consistent with those previously reported.[14]

3-{1-[(4-Amino-2-methylpyrimidin-5-yl)methyl]-1H-1,2,3-triazol-4-yl}propyl 4-methylbenzene-1-sulfonate **10b**

White solid (1.57 g, 78%). ^{1}H NMR (400 MHz, CD_3OD) δ 8.04 (s, 1H), 7.76 (d, 2H, J = 8.2 Hz), 7.74 (s, 1H), 7.42 (d, 2H, J = 8.2 Hz), 5.45 (s, 2H), 4.06 (t, 2H, J = 6.1 Hz), 2.72 (t, 2H, J = 7.6 Hz), 2.45 (s, 3H), 2.42 (s, 3H), 1.99 (m, 2H). ESI-MS m/z: [M+H$^+$] calculated for $C_{18}H_{22}N_6O_3S$: 403.155; found: 403.16.

4-{1-[(4-Amino-2-methylpyrimidin-5-yl)methyl]-1H-1,2,3-triazol-4-yl}butyl 4-methylbenzene-1-sulfonate **10c**

White solid (1.56 g, 75%). ^{1}H NMR (400 MHz, CD_3OD) δ 8.05 (s, 1H), 7.77 (d, 2H, J = 7.9 Hz), 7.75 (s, 1H), 7.43 (d, 2H, J = 7.9 Hz), 5.46 (s, 2H), 4.04 (t, 2H, J = 5.5 Hz), 2.64 (t, 2H, J = 6.6 Hz), 2.44 (s, 3H), 2.42 (s, 3H), 1.66 (m, 4H). ESI-MS m/z: [M+H$^+$] calculated for $C_{19}H_{24}N_6O_3S$: 417.171; found: 417.17.

General procedure for preparation of 11a-c:

To a stirred suspension of tosylate **10a-c** (3 mmol, 1 equiv.) in dry DMF (1 M) under nitrogen at 0 °C was added NaN_3 (2 equiv.). The resultant mixture was stirred at r.t. for 2 days. The reaction mixture was quenched with aqueous phosphate buffer (pH 7) and extracted with *n*-BuOH. The organic phase was dried over $MgSO_4$, filtered, and evaporated under reduced pressure. The residue was purified by silica flash chromatography (10% MeOH in DCM) to yield azide **11a-c** as a solid.

5-{[4-(2-Azidoethyl)-1H-1,2,3-triazol-1-yl]methyl}-2-methylpyrimidin-4-amine **11a**

White solid (513 mg, 66%). ^{1}H NMR (400 MHz, CD_3OD) δ 8.05 (s, 1H), 7.89 (s, 1H), 5.49 (s, 2H), 3.59 (t, 2H, J = 6.7 Hz), 2.97 (t, 2H, J = 6.7 Hz), 2.42 (s, 3H). ^{13}C NMR (100 MHz, CD_3OD) δ 167.6, 162.2, 155.0, 145.0, 122.8, 108.5, 50.3, 47.3, 24.9, 23.7. ESI-MS m/z: [M+H$^+$] calculated for $C_{10}H_{13}N_9$: 260.137; found: 260.14.

5-{[4-(3-Azidopropyl)-1H-1,2,3-triazol-1-yl]methyl}-2-methylpyrimidin-4-amine **11b**

White solid (565 mg, 69%). ^{1}H NMR (400 MHz, CD_3OD) δ 8.06 (s, 1H), 7.82 (s, 1H), 5.48 (s, 2H), 3.35 (t, 2H, J = 6.8 Hz), 2.79 (t, 2H, J = 7.6 Hz), 2.43 (s, 3H), 1.94 (m, 2H). ^{13}C NMR (100 MHz, CD_3OD) δ 167.6, 162.0, 155.0, 147.2, 122.2, 108.5, 50.3, 47.2, 28.2, 23.7, 22.0. ESI-MS m/z: [M+H$^+$] calculated for $C_{11}H_{15}N_9$: 274.153; found: 274.15.

5-{[4-(4-Azidobutyl)-1H-1,2,3-triazol-1-yl]methyl}-2-methylpyrimidin-4-amine **11c**

White solid (620 mg, 72%). ^{1}H NMR (400 MHz, CD_3OD) δ 8.05 (s, 1H), 7.80 (s, 1H), 5.47 (s, 2H), 3.31 (t, 2H, J = 6.7 Hz), 2.73 (t, 2H, J = 7.6 Hz), 2.42 (s, 3H), 1.74 (m, 2H), 1.61 (m, 2H). ^{13}C NMR (100 MHz, CD_3OD) δ 167.6, 162.1, 155.0, 147.9, 121.9, 108.7, 50.7, 47.2, 27.9, 26.2, 24.4, 23.7. ESI-MS m/z: [M+H$^+$] calculated for $C_{12}H_{17}N_9$: 288.168; found: 288.16.

General procedure for preparation of 12a-c:

A stirred solution of azide **11a-c** (1 mmol, 1 equiv.) in MeOH (0.1 M) at r.t. was treated with 10% Pd/C (25 mg) under nitrogen. The flask was evacuated and flushed with hydrogen gas (three times). The resultant mixture was stirred vigorously at r.t. under an atmosphere of hydrogen (1 atm, H_2 balloon) for 4 h. The reaction mixture was filtered through Celite and concentrated under reduced pressure to yield amine **12a-c** as a solid, which was used in the next step without further purification.

5-{[4-(2-Aminoethyl)-1H-1,2,3-triazol-1-yl]methyl}-2-methylpyrimidin-4-amine **12a**

White solid (224 mg, 96%). ^{1}H NMR (400 MHz, CD_3OD) δ 8.06 (s, 1H), 7.83 (s, 1H), 5.48 (s, 2H), 2.95 (t, 2H, J = 7.0 Hz), 2.86 (t, 2H, J = 7.0 Hz), 2.42 (s, 3H). ^{13}C NMR (100 MHz, CD_3OD) δ 167.5, 162.1, 155.1, 145.6, 122.5, 108.6, 47.2, 40.6, 27.8, 23.6. ESI-MS m/z: [M+H$^+$] calculated for $C_{10}H_{15}N_7$: 234.147; found: 234.14.

5-{[4-(3-Aminopropyl)-1H-1,2,3-triazol-1-yl]methyl}-2-methylpyrimidin-4-amine **12b**

White solid (242 mg, 98%). ^{1}H NMR (400 MHz, CD_3OD) δ 8.05 (s, 1H), 7.80 (s, 1H), 5.47 (s, 2H), 2.76 (t, 2H, J = 7.6 Hz), 2.70 (t, 2H, J = 6.9 Hz), 2.43 (s, 3H) 1.83 (m, 2H). ^{13}C NMR (100 MHz, CD_3OD) δ 167.6, 162.1, 155.0, 147.8, 121.7, 108.6, 47.2, 40.2, 31.5, 23.5, 22.3. ESI-MS m/z: [M+H$^+$] calculated for $C_{11}H_{17}N_7$: 248.162; found: 248.16.

5-{[4-(4-Aminobutyl)-1H-1,2,3-triazol-1-yl]methyl}-2-methylpyrimidin-4-amine **12c**

White solid (249 mg, 95%). ^{1}H NMR (400 MHz, CD_3OD) δ 8.05 (s, 1H), 7.80 (s, 1H), 5.47 (s, 2H), 2.72 (t, 2H, J = 7.6 Hz), 2.67 (t, 2H, J = 7.2 Hz), 2.42 (s, 3H) 1.70 (m, 2H), 1.52 (m, 2H). ^{13}C NMR (100 MHz, CD_3OD) δ 167.6, 162.2, 155.0, 148.3, 121.9, 108.7, 47.2, 40.7, 31.5, 26.3, 24.7, 23.7. ESI-MS m/z: [M+H$^+$] calculated for $C_{12}H_{19}N_7$: 262.178; found: 262.18.

General procedure for preparation of 13a-14a:

To a stirred solution of the corresponding carboxylic acid (0.39 mmol, 1.3 equiv.) and DCC (3 equiv.) in dry DMF (0.2 M) under nitrogen at 0 °C was added DMAP (1.3 equiv.) and alcohol **9a** (0.3 mmol, 1 equiv.). The resultant mixture was stirred at r.t. for 2 days, then diluted with DCM, filtered through cotton wool (to remove DCC/DCU), washed with aqueous phosphate buffer (pH 7), and extracted with DCM. The organic phase was dried over $MgSO_4$, filtered, and evaporated under reduced pressure. The residue was purified by silica flash chromatography (10% MeOH in DCM) to yield ester **13a-14a** as a solid.

2-{1-[(4-Amino-2-methylpyrimidin-5-yl)methyl]-1H-1,2,3-triazol-4-yl}ethyl 3-[5-(trifluoromethyl)-1,2,4-oxadiazol-3-yl]benzoate **13a**

Prepared from 3-(5-(trifluoromethyl)-1,2,4-oxadiazol-3-yl)benzoic acid (obtained through a two-step synthesis from 3-cyanobenzoic acid, see below)[15]. White solid (122 mg, 86%). m.p. 200-201 ºC. ^{1}H NMR (400 MHz, CD_3OD) δ 8.67 (s, 1H), 8.35 (d, 1H, J = 7.7 Hz), 8.17 (d, 1H, J = 7.7 Hz), 8.02 (s, 1H), 7.91 (s, 1H), 7.70 (t, 1H, J = 7.7 Hz), 5.48 (s, 2H), 4.64 (t, 2H, J = 6.4 Hz), 3.23 (t, 2H, J = 6.4 Hz), 2.38 (s, 3H). ^{13}C NMR (100 MHz, CD_3OD) δ 168.4, 167.6, 166.3 (q, J = 45 Hz), 165.1, 162.0, 155.1, 144.4, 132.6, 131.5, 131.1, 129.5, 128.0, 125.6, 122.7, 116.0 (q, J = 272 Hz), 108.4, 63.8, 47.1, 24.8, 23.6. ^{19}F NMR (376 MHz, CD_3OD) δ -68.2. HRMS (ESI) m/z: [M+H$^+$] calculated for $C_{20}H_{17}F_3N_8O_3$: 475.1454; found: 475.1444.

The previous work[15] had reported the ^{1}H NMR data for the following two compounds; thus, additional ^{13}C and ^{19}F NMR data are provided here. The work-up procedure has been slightly modified.

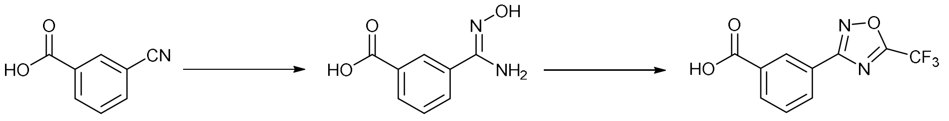

3-cyanobenzoic acid

3-(N'-Hydroxycarbamimidoyl) benzoic acid,
Previously reported yield: 82%
Yield obtained in this work: 87%

3-(5-(Trifluoromethyl)-1,2,4-oxadiazol-3-yl) benzoic acid,
Previously reported yield: 28%
Yield obtained in this work: 28%

3-(N'-Hydroxycarbamimidoyl)benzoic acid

To a stirred solution of 3-cyanobenzoic acid (500 mg, 3.4 mmol) in EtOH (25 mL) was added 8-hydroxyquinoline (2.5 mg, 0.017 mmol), hydroxylamine hydrochloride (473 mg, 6.8 mmol) in water (4 mL), and sodium carbonate (580 mg, 5.5 mmol) in water (6 mL). The resultant mixture was heated under reflux for 4 hours and then concentrated under reduced pressure. The residue was purified by silica flash chromatography (15% MeOH in DCM) to yield 3-(N'-Hydroxycarbamimidoyl)benzoic acid as a yellow solid (535 mg, 87%). ^{1}H NMR (400 MHz, CD_3OD) δ 8.23 (s, 1H), 8.06 (d, 1H, J = 6.9 Hz), 7.77 (d, 1H, J = 6.9 Hz), 7.46 (t, 1H, J = 6.9 Hz). ^{1}H NMR data consistent with those reported.[15] ^{13}C NMR (100 MHz, CD_3OD) δ 171.2, 154.1, 135.3, 132.6, 130.3, 128.7, 127.8, 127.0.

3-(5-(Trifluoromethyl)-1,2,4-oxadiazol-3-yl)benzoic acid

To a stirred solution of 3-(N'-hydroxycarbamimidoyl)benzoic acid (535 mg, 3 mmol) in dry pyridine (9 mL, 0.33 M) under nitrogen at 0 °C was added trifluoroacetic anhydride (1.25 mL, 9 mmol) dropwise. The resultant mixture was stirred at r.t. for 15 min and then at 50 °C for 3 h. The reaction mixture was diluted with cold water (35 mL), acidified with 1 M HCl to pH = 4, and extracted with EtOAc (2 x 100 mL). The combined organic phases were dried over $MgSO_4$, filtered, and evaporated under reduced pressure. The residue was purified by silica flash chromatography (20% EtOAc in hexane) to yield 3-(5-(trifluoromethyl)-1,2,4-oxadiazol-3-yl)benzoic acid as a white solid (210 mg, 28%). ^{1}H NMR (400 MHz, $CDCl_3$) δ 8.90 (s, 1H), 8.40 (d, 1H, J = 7.8 Hz), 8.35 (d, 1H, J = 7.8 Hz), 7.70 (t, 1H, J = 7.8 Hz). ^{1}H NMR data consistent with those reported.[15] ^{13}C NMR (100 MHz, $CDCl_3$) δ 170.7, 168.5, 166.2 (q, J = 44.7 Hz), 133.7, 132.6, 130.5, 129.5, 129.1, 125.8, 115.9 (q, J = 273 Hz). ^{19}F NMR (376 MHz, CD_3OD) δ -66.3.

2-{1-[(4-Amino-2-methylpyrimidin-5-yl)methyl]-1H-1,2,3-triazol-4-yl}ethyl 3-cyanobenzoate **14a**

Prepared from 3-cyanobenzoic acid. White solid (65 mg, 60%). m.p. 165-166 °C. ^{1}H NMR (400 MHz, CD_3OD) δ 8.28 (s, 1H), 8.21 (d, 1H, J = 7.9 Hz), 8.03 (s, 1H), 7.98 (d, 1H, J = 7.9 Hz), 7.91 (s, 1H), 7.67 (t, 1H, J = 7.9 Hz), 5.48 (s, 2H), 4.61 (t, 2H, J = 6.4 Hz), 3.21 (t, 2H, J = 6.4 Hz), 2.41 (s, 3H). ^{13}C NMR (100 MHz, CD_3OD) δ 167.6, 164.4, 162.1, 154.9, 144.4, 136.0, 133.3, 132.7, 131.2, 129.6, 122.7, 117.4, 112.6, 108.5, 64.0, 47.1, 24.8, 23.6. HRMS (ESI) m/z: [M+H$^+$] calculated for $C_{18}H_{17}N_7O_2$: 364.1521; found: 364.1528.

General procedure for preparation of 15a-c:

To a stirred solution of corresponding azide **11a-c** (0.2 mmol, 1 equiv.) and phenylacetylene (1 equiv.) in *t*-BuOH and water (2:1, 0.2 M) was added $CuSO_4.5H_2O$ (0.03 equiv.) and sodium ascorbate (0.3 equiv.). The resultant mixture was stirred at r.t. for 2-4 days. The reaction mixture was concentrated under reduced pressure, diluted in EtOAc, washed with 1 M K_2CO_3, dried over $MgSO_4$, filtered, and evaporated under reduced pressure. The residue was purified by silica flash chromatography (10% MeOH in DCM) to yield bis-triazole **15a-c** as a solid.

2-Methyl-5-({4-[2-(4-phenyl-1H-1,2,3-triazol-1-yl)ethyl]-1H-1,2,3-triazol-1-yl}methyl)pyrimidin-4-amine **15a**

Prepared from **11a**. White solid (38 mg, 53%). m.p. 244-245 °C. ^{1}H NMR (400 MHz, CD_3SOCD_3) δ 8.53 (s, 1H), 7.93 (s, 1H), 7.89 (s, 1H), 7.81 (d, 2H, J = 7.7 Hz), 7.45 (m, 2H), 7.33 (t, 1H, J = 7.4 Hz), 6.88 (br, 2H, NH_2), 5.40 (s, 2H), 4.70 (t, 2H, J = 7.4 Hz), 3.28 (t, 2H, J = 7.4 Hz), 2.31 (s, 3H). ^{13}C NMR (100 MHz, CD_3SOCD_3) δ 167.3, 161.8, 156.5, 146.7, 143.4, 131.3, 129.4, 128.3, 125.7, 123.3, 121.8, 108.8, 49.4, 47.1, 26.6, 25.8. HRMS (ESI) m/z: [M+H$^+$] calculated for $C_{18}H_{19}N_9$: 362.1841; found: 362.1849.

2-Methyl-5-({4-[3-(4-phenyl-1H-1,2,3-triazol-1-yl)propyl]-1H-1,2,3-triazol-1-yl}methyl)pyrimidin-4-amine **15b**

Prepared from **11b**. White solid (44 mg, 58%). m.p. 235-236 °C. ^{1}H NMR (400 MHz, CD_3OD) δ 8.32 (s, 1H), 8.04 (s, 1H), 7.81 (m, 2H), 7.80 (s, 1H), 7.44 (m, 2H), 7.35 (m, 1H), 5.45 (s, 2H), 4.50 (t, 2H, J = 7.0 Hz), 2.76 (t, 2H, J = 7.5 Hz), 2.42 (s, 3H), 2.33 (m, 2H). ^{13}C NMR (100 MHz, CD_3OD) δ 167.7, 162.1, 155.1, 147.5, 146.8, 130.3, 128.6, 127.9, 125.3, 122.3, 120.8, 108.5, 49.3, 47.2, 29.4, 23.6, 21.8. HRMS (ESI) m/z: [M+H$^+$] calculated for $C_{19}H_{21}N_9$: 376.1998; found: 376.1992.

2-Methyl-5-({4-[4-(4-phenyl-1H-1,2,3-triazol-1-yl)butyl]-1H-1,2,3-triazol-1-yl}methyl)pyrimidin-4-amine **15c**

Prepared from **11c**. White solid (39 mg, 51%). m.p. 245-246 °C. ^{1}H NMR (400 MHz, CD_3SOCD_3) δ 8.56 (s, 1H), 7.93 (s, 1H), 7.84 (s, 1H), 7.83 (d, 2H, J = 8.2 Hz), 7.45 (m, 2H), 7.33 (t, 1H, J = 7.4 Hz), 6.85 (br, 2H, NH_2), 5.36 (s, 2H), 4.42 (t, 2H, J = 7.0 Hz), 2.65 (t, 2H, J = 7.7 Hz), 2.30 (s, 3H), 1.91 (m, 2H), 1.58 (m, 2H). ^{13}C NMR (100 MHz, CD_3SOCD_3) δ 167.3, 161.9, 156.5, 147.0, 146.8, 131.3, 129.4, 128.3, 125.6, 122.5, 121.8, 108.9, 49.7, 47.0, 29.7, 26.3, 25.6, 24.8. HRMS (ESI) m/z: [M+H$^+$] calculated for $C_{20}H_{23}N_9$: 390.2154; found: 390.2164.

General procedure for preparation of 16a-c:

To a stirred solution of corresponding azide **11a-c** (0.2 mmol, 1 equiv.) and 1-ethynyl-4-fluorobenzene (1 equiv.) in *t*-BuOH and water (2:1, 0.2 M) was added $CuSO_4.5H_2O$ (0.03 equiv.) and sodium ascorbate (0.3 equiv.). The resultant mixture was stirred at r.t. for 2-4 days, then concentrated under reduced pressure, diluted with EtOAc, washed with 1 M K_2CO_3, dried over $MgSO_4$, filtered, and evaporated under reduced pressure. The residue was purified by silica flash chromatography (10% MeOH in DCM) to yield bis-triazole **16a-c** as a solid.

5-[(4-{2-[4-(4-Fluorophenyl)-1H-1,2,3-triazol-1-yl]ethyl}-1H-1,2,3-triazol-1-yl)methyl]-2-methylpyrimidin-4-amine **16a**

Prepared from **11a**. White solid (46 mg, 61%). m.p. 258-259 °C. ^{1}H NMR (400 MHz, CD_3SOCD_3) δ 8.52 (s, 1H), 7.93 (s, 1H), 7.89 (s, 1H), 7.85 (m, 2H), 7.29 (m, 2H), 6.89 (br, 2H, NH_2), 5.39 (s, 2H), 4.69 (t, 2H, J = 7.0 Hz), 3.28 (t, 2H, J = 7.0 Hz), 2.31 (s, 3H). ^{13}C NMR (100 MHz, CD_3SOCD_3) δ 167.5, 162.2 (d, J = 245 Hz), 162.0, 156.5, 145.7, 143.4, 127.8 (d, J = 3 Hz), 127.6 (d, J = 8 Hz), 123.4, 121.8, 116.3 (d, J = 22 Hz), 108.8, 49.5, 47.1, 26.6, 25.7. ^{19}F NMR (376 MHz, CD_3SOCD_3) δ -114.2. HRMS (ESI) m/z: [M+H$^+$] calculated for $C_{18}H_{18}FN_9$: 380.1747; found: 380.1740.

5-[(4-{3-[4-(4-Fluorophenyl)-1H-1,2,3-triazol-1-yl]propyl}-1H-1,2,3-triazol-1-yl)methyl]-2-methylpyrimidin-4-amine **16b**

Prepared from **11b**. White solid (45 mg, 57%). m.p. 245-246 °C. ^{1}H NMR (400 MHz, CD_3OD) δ 8.31 (s, 1H), 8.04 (s, 1H), 7.83 (m, 2H), 7.81 (s, 1H), 7.18 (m, 2H), 5.45 (s, 2H), 4.50 (t, 2H, J = 6.9 Hz), 2.76 (t, 2H, J = 7.5 Hz), 2.42 (s, 3H), 2.33 (m, 2H). ^{13}C NMR (100 MHz, CD_3OD) δ 167.6, 162.7 (d, J = 246 Hz), 162.1, 155.2, 146.7, 146.6, 127.3 (d, J = 8 Hz), 126.6 (d, J = 3 Hz), 122.4, 120.8, 115.3 (d, J = 22 Hz), 108.5, 49.3, 47.3, 29.6, 23.7, 21.8. ^{19}F NMR (376 MHz, CD_3OD) δ -116.6. HRMS (ESI) m/z: [M+H$^+$] calculated for $C_{19}H_{20}FN_9$: 394.1903; found: 394.1918.

5-[(4-{4-[4-(4-Fluorophenyl)-1H-1,2,3-triazol-1-yl]butyl}-1H-1,2,3-triazol-1-yl)methyl]-2-methylpyrimidin-4-amine **16c**

Prepared from **11c**. White solid (42 mg, 52%). m.p. 262-263 °C. ^{1}H NMR (400 MHz, CD_3OD) δ 8.29 (s, 1H), 8.03 (s, 1H), 7.83 (m, 2H), 7.78 (s, 1H), 7.19 (m, 2H), 5.45 (s, 2H), 4.47 (t, 2H, J = 7.1 Hz), 2.76 (t, 2H, J = 7.6 Hz), 2.42 (s, 3H), 2.00 (m, 2H), 1.71 (m, 2H). ^{13}C NMR (100 MHz, CD_3OD) δ 167.6, 162.7 (d, J = 245 Hz), 162.1, 154.9, 147.7, 146.6, 127.2 (d, J = 8 Hz), 126.8 (d, J = 3 Hz), 122.0, 120.7, 115.4 (d, J = 22 Hz), 108.7, 49.8, 47.1, 29.2,25.9, 24.1, 23.6. ^{19}F NMR (376 MHz, CD_3OD) δ -116.7. HRMS (ESI) m/z: [M+H$^+$] calculated for $C_{20}H_{22}FN_9$: 408.2060; found: 408.2051.

General procedure for preparation of compound 17a-c:

To a stirred solution of corresponding azide **11a-c** (0.2 mmol, 1 equiv.) and 4-ethynylanisole (1 equiv.) in *t*-BuOH and water (2:1, 0.2 M) was added $CuSO_4.5H_2O$ (0.03 equiv.) and sodium ascorbate (0.3 equiv.). The resultant mixture was stirred at r.t. for 2-4 days. The reaction mixture was concentrated under reduced pressure, diluted in EtOAc, washed with 1 M K_2CO_3, dried over $MgSO_4$, filtered, and evaporated under reduced pressure. The residue was purified by silica flash chromatography (10% MeOH in DCM) to yield bis-triazole **17a-c** as a solid.

5-[(4-{2-[4-(4-Methoxyphenyl)-1H-1,2,3-triazol-1-yl]ethyl}-1H-1,2,3-triazol-1-yl)methyl]-2-methylpyrimidin-4-amine **17a**

Prepared from **11a**. White solid (39 mg, 50%). m.p. 238-239 °C. ^{1}H NMR (400 MHz, CD_3SOCD_3) δ 8.42 (s, 1H), 7.93 (s, 1H), 7.89 (s, 1H), 7.74 (d, 2H, J = 8.8 Hz), 7.02 (d, 2H, J = 8.8 Hz), 6.89 (br, 2H, NH_2), 5.40 (s, 2H), 4.67 (t, 2H, J = 7.2 Hz), 3.80 (s, 3H), 3.27 (t, 2H, J = 7.2 Hz), 2.31 (s, 3H). ^{13}C NMR (100 MHz, CD_3SOCD_3) δ 167.3, 161.9, 159.5, 156.5, 146.6, 143.5, 127.0, 123.9, 123.3, 120.9, 114.8, 108.9, 55.6, 49.3, 47.1, 26.8, 25.8. HRMS (ESI) m/z: [M+H$^+$] calculated for $C_{19}H_{21}N_9O$: 392.1947; found: 392.1957.

5-[(4-{3-[4-(4-Methoxyphenyl)-1H-1,2,3-triazol-1-yl]propyl}-1H-1,2,3-triazol-1-yl)methyl]-2-methylpyrimidin-4-amine **17b**

Prepared from **11b**. White solid (37 mg, 45%). m.p. 241-242 °C. ^{1}H NMR (400 MHz, CD_3SOCD_3) δ 8.48 (s, 1H), 7.97 (s, 1H), 7.92 (s, 1H), 7.77 (d, 2H, J = 8.5 Hz), 7.01 (d, 2H, J = 8.5 Hz), 6.96 (br, 2H, NH_2), 5.40 (s, 2H), 4.43 (t, 2H, J = 6.8 Hz), 3.79 (s, 3H), 2.65 (t, 2H, J = 7.6 Hz), 2.32 (s, 3H), 2.20 (m, 2H). ^{13}C NMR (100 MHz, CD_3SOCD_3) δ 167.2, 162.0, 159.4, 156.2, 146.7, 146.2, 127.0, 123.9, 122.7,

120.9, 114.7, 108.9, 55.7, 49.5, 47.0, 29.9, 25.5, 22.5. HRMS (ESI) m/z: [M+H^+] calculated for $C_{20}H_{23}N_9O$: 406.2104; found: 406.2119.

5-[(4-{4-[4-(4-Methoxyphenyl)-1H-1,2,3-triazol-1-yl]butyl}-1H-1,2,3-triazol-1-yl)methyl]-2-methylpyrimidin-4-amine **17c**

Prepared from **11c**. White solid (50 mg, 59%). m.p. 259-260 °C. ^{1}H NMR (400 MHz, CD_3SOCD_3) δ 8.45 (s, 1H), 7.94 (s, 1H), 7.85 (s, 1H), 7.76 (d, 2H, J = 8.8 Hz), 7.01 (d, 2H, J = 8.8 Hz), 6.82 (br, 2H, NH_2), 5.37 (s, 2H), 4.40 (t, 2H, J = 7.0 Hz), 3.79 (s, 3H), 2.65 (t, 2H, J = 7.6 Hz), 2.30 (s, 3H), 1.90 (m, 2H), 1.58 (m, 2H). ^{13}C NMR (100 MHz, CD_3SOCD_3) δ 167.4, 161.9, 159.4, 156.4, 147.0, 146.7, 127.1, 123.9, 122.6, 120.9, 114.8, 108.9, 55.5, 49.7, 47.0, 29.7, 26.4, 25.7, 24.8. HRMS (ESI) m/z: [M+H^+] calculated for $C_{21}H_{25}N_9O$: 420.2262; found: 420.2278.

General procedure for preparation of 18a-c:

To a stirred solution of corresponding amine **12a-c** (0.2 mmol, 1 equiv.) in dry pyridine (0.2 M) under nitrogen at 0 °C was added trifluoromethanesulfonic anhydride (1.05 equiv.) dropwise. The resultant mixture was stirred at 35 °C for 3 h and then concentrated under reduced pressure. The residue was purified by silica flash chromatography (12% MeOH in DCM) to yield sulfonamide **18a-c** as a solid.

N-(2-{1-[(4-Amino-2-methylpyrimidin-5-yl)methyl]-1H-1,2,3-triazol-4-yl}ethyl)-1,1,1-trifluoromethanesulfonamide **18a**

Prepared from **12a**. White solid (40 mg, 55%). m.p. 158-159 °C. ^{1}H NMR (400 MHz, CD_3OD) δ 8.05 (s, 1H), 7.95 (s, 1H), 5.58 (s, 2H), 3.52 (t, 2H, J = 6.8 Hz), 2.99 (t, 2H, J = 6.8 Hz), 2.57 (s, 3H). ^{13}C NMR (100 MHz, CD_3OD) δ 163.7, 161.9, 144.4, 142.9, 123.4, 120.3 (q, J = 320 Hz), 109.9, 45.9, 43.0, 26.3, 20.1. ^{19}F NMR (376 MHz, CD_3OD) δ -81.0. HRMS (ESI) m/z: [M+H^+] calculated for $C_{11}H_{14}F_3N_7O_2S$: 366.0960; found: 366.0965.

N-(3-{1-[(4-Amino-2-methylpyrimidin-5-yl)methyl]-1H-1,2,3-triazol-4-yl}propyl)-1,1,1-trifluoromethanesulfonamide **18b**

Prepared from **12b**. White solid (49 mg, 65%). m.p. 164-165 °C. ^{1}H NMR (400 MHz, CD_3OD) δ 8.13 (s, 1H), 7.93 (s, 1H), 5.56 (s, 2H), 3.02 (t, 2H, J = 7.6 Hz), 2.84 (t, 2H, J = 7.6 Hz), 2.56 (s, 3H), 2.04 (m, 2H). ^{13}C NMR (100 MHz, CD_3OD) δ 163.7, 162.7, 146.5, 145.0, 122.8, 120.3 (q, J = 315 Hz), 109.4, 46.3, 38.8, 26.8, 21.8, 20.7. ^{19}F NMR (376 MHz, CD_3OD) δ -80.1. HRMS (ESI) m/z: [M+H^+] calculated for $C_{12}H_{16}F_3N_7O_2S$: 380.1116; found: 380.1106.

N-(4-{1-[(4-Amino-2-methylpyrimidin-5-yl)methyl]-1H-1,2,3-triazol-4-yl}butyl)-1,1,1-trifluoromethanesulfonamide **18c**

Prepared from **12c**. White solid (49 mg, 62%). m.p. 168-169 °C. ^{1}H NMR (400 MHz, CD_3OD) δ 8.09 (s, 1H), 7.89 (s, 1H), 5.53 (s, 2H), 2.97 (t, 2H, J = 7.0 Hz), 2.78 (t, 2H, J = 7.4 Hz), 2.51 (s, 3H), 1.75 (m, 4H). ^{13}C NMR (100 MHz, CD_3OD) δ 164.7, 163.0, 148.7, 147.5, 122.5, 120.4 (q, J = 319 Hz), 109.1, 46.6, 38.8, 26.5, 25.7, 24.2, 21.8. ^{19}F NMR (376 MHz, CD_3OD) δ -80.0. HRMS (ESI) m/z: [M+H^+] calculated for $C_{13}H_{18}F_3N_7O_2S$: 394.1273; found: 394.1276.

General procedure for preparation of 19a-20a:

To a stirred solution of the corresponding carboxylic acid (0.39 mmol, 1.3 equiv.) and DCC (3 equiv.) in dry DMF (0.2 M) under nitrogen at 0 °C was added DMAP (1.3 equiv.) and amine **12a** (0.3 mmol, 1 equiv.). The resultant mixture was stirred at r.t. for 2 days. The reaction mixture was diluted with DCM, filtered through cotton wool (to remove DCC/DCU), washed with aqueous phosphate buffer (pH 7), and extracted with DCM. The organic phase was dried over $MgSO_4$, filtered, and evaporated under reduced pressure. The residue was purified by silica flash chromatography (12% MeOH in DCM) to yield amide **19a-20a** as a solid.

N-(2-{1-[(4-Amino-2-methylpyrimidin-5-yl)methyl]-1H-1,2,3-triazol-4-yl}ethyl)-3-[5-(trifluoromethyl)-1,2,4-oxadiazol-3-yl]benzamide **19a**

Prepared from 3-(5-(trifluoromethyl)-1,2,4-oxadiazol-3-yl)benzoic acid. White solid (71 mg, 50%). m.p. 211-212 ºC. ^{1}H NMR (400 MHz, CD_3OD) δ 8.53 (s, 1H), 8.27 (d, 1H, *J* = 7.7 Hz), 8.05 (s, 1H), 7.99 (d, 1H, *J* = 7.7 Hz), 7.87 (s, 1H), 7.67 (t, 1H, *J* = 7.7 Hz), 5.48 (s, 2H), 3.70 (t, 2H, *J* = 7.0 Hz), 3.05 (t, 2H, *J* = 7.0 Hz), 2.38 (s, 3H). ^{13}C NMR (100 MHz, CD_3OD) δ 168.7, 167.6, 167.5, 166.2 (q, *J* = 48 Hz), 162.2, 155.1, 145.4, 135.5, 130.5, 130.0, 129.3, 126.1, 125.4, 122.4, 116.0 (q, *J* = 275 Hz), 108.6, 47.1, 39.3, 25.0, 23.6. ^{19}F NMR (376 MHz, CD_3OD) δ -68.2. HRMS (ESI) m/z: [M+H$^+$] calculated for $C_{20}H_{18}F_3N_9O_2$: 474.1613; found: 474.1616.

N-(2-{1-[(4-Amino-2-methylpyrimidin-5-yl)methyl]-1H-1,2,3-triazol-4-yl}ethyl)-3-cyanobenzamide **20a**

Prepared from 3-cyanobenzoic acid. White solid (48 mg, 45%). m.p. 172-173 ºC. ^{1}H NMR (400 MHz, CD_3SOCD_3) δ 8.79 (br, NH), 8.23 (s, 1H), 8.11 (d, 1H, *J* = 7.7 Hz), 8.00 (d, 1H, *J* = 7.7 Hz), 7.93 (s, 1H), 7.92 (s, 1H), 7.69 (t, 1H, *J* = 7.7 Hz), 6.87 (br, NH_2), 5.39 (s, 2H), 3.53 (m, 2H), 2.90 (t, 2H, *J* = 7.1 Hz), 2.30 (s, 3H). ^{13}C NMR (100 MHz, CD_3SOCD_3) δ 167.4, 164.8, 161.8, 156.3, 144.8, 136.0, 135.1, 132.4, 131.2, 130.2, 123.0, 118.8, 111.9, 108.9, 47.0, 39.7, 25.7, 25.6. HRMS (ESI) m/z: [M+H$^+$] calculated for $C_{18}H_{18}N_8O$: 363.1682; found: 363.1678.

General procedure for preparation of 21a-c:

To a stirred solution of 3-methoxybenzoic acid (0.65 mmol, 1.3 equiv.) and DCC (3 equiv.) in dry DMF (0.2 M) under nitrogen at 0 °C was added DMAP (1.3 equiv.) and corresponding amine **12a-c** (0.5 mmol, 1 equiv.). The resultant mixture was stirred at r.t. for 2 days, then diluted with DCM, filtered through cotton wool (to remove DCC/DCU), washed with aqueous phosphate buffer (pH 7), and the washings extracted with DCM. The organic phases were dried over $MgSO_4$, filtered, and evaporated under reduced pressure. The residue was purified by silica flash chromatography (12% MeOH in DCM) to yield amide **21a-c** as a solid.

N-(2-{1-[(4-Amino-2-methylpyrimidin-5-yl)methyl]-1H-1,2,3-triazol-4-yl}ethyl)-3-methoxybenzamide **21a**

Prepared from **12a**. White solid (75 mg, 41%). m.p. 172-173 ºC. ^{1}H NMR (400 MHz, CD_3SOCD_3) δ 8.53 (br, 1H, NH), 7.94 (s, 1H), 7.92 (s, 1H), 7.40 (m, 3H), 7.12 (m, 1H), 6.88 (br, 2H, NH_2), 5.39 (s, 2H), 3.80 (s, 3H), 3.50 (m, 2H), 2.88 (t, 2H, *J* = 7.3 Hz), 2.31 (s, 3H). ^{13}C NMR (100 MHz, CD_3SOCD_3) δ 167.3, 166.5, 161.9, 159.6, 156.7, 145.0, 136.5, 129.9, 122.9, 119.8, 117.4, 112.8, 108.9, 55.8, 47.1, 39.7, 25.8, 25.6. HRMS (ESI) m/z: [M+H$^+$] calculated for $C_{18}H_{21}N_7O_2$: 368.1835; found: 368.1841.

N-(3-{1-[(4-Amino-2-methylpyrimidin-5-yl)methyl]-1H-1,2,3-triazol-4-yl}propyl)-3-methoxybenzamide **21b**

Prepared from **12b**. White solid (82 mg, 43%). m.p. 183-184 ºC. ^{1}H NMR (400 MHz, CD_3OD) δ 8.04 (s, 1H), 7.83 (s, 1H), 7.36 (m, 3H), 7.09 (m, 1H), 5.46 (s, 2H), 3.85 (s, 3H), 3.42 (t, 2H, *J* = 6.9 Hz), 2.79 (t, 2H, *J* = 7.5 Hz), 2.42 (s, 3H), 1.97 (m, 2H). ^{13}C NMR (100 MHz, CD_3OD) δ 168.7, 167.6, 162.2, 159.9, 155.1, 147.6, 135.7, 129.3, 122.1, 118.9, 117.0, 112.2, 108.6, 54.5, 47.2, 38.9, 28.8, 23.7, 22.5. HRMS (ESI) m/z: [M+H$^+$] calculated for $C_{19}H_{23}N_7O_2$: 382.1991; found: 382.1997.

N-(4-{1-[(4-Amino-2-methylpyrimidin-5-yl)methyl]-1H-1,2,3-triazol-4-yl}butyl)-3-methoxybenzamide **21c**

Prepared from **12c**. White solid (89 mg, 45%). m.p. 188-189 ºC. ^{1}H NMR (400 MHz, CD_3OD) δ 8.03 (s, 1H), 7.79 (s, 1H), 7.36 (m, 3H), 7.09 (m, 1H), 5.46 (s, 2H), 3.85 (s, 3H), 3.40 (t, 2H, *J* = 6.6 Hz), 2.76 (t, 2H, *J* = 7.6 Hz), 2.42 (s, 3H), 1.60-1.80 (m, 4H). ^{13}C NMR (100 MHz, CD_3OD) δ 168.6, 167.6, 162.2, 159.9, 155.0, 148.1, 135.8, 129.2, 122.0, 119.0, 117.0, 112.2, 108.6, 54.5, 47.2, 39.2, 28.5, 26.4, 24.5, 23.6. HRMS (ESI) m/z: [M+H$^+$] calculated for $C_{20}H_{25}N_7O_2$: 396.2148; found: 396.2152.

General procedure for preparation of 22a-c:

To a stirred solution of 2-methoxybenzoic acid (0.65 mmol, 1.3 equiv.) and DCC (3 equiv.) in dry DMF (0.2 M) under nitrogen at 0 °C was added DMAP (1.3 equiv.) and corresponding amine **12a-c** (0.5 mmol, 1 equiv.). The resultant mixture was stirred at r.t. for 2 days, then diluted with DCM, filtered through cotton wool (to remove DCC/DCU), washed with aqueous phosphate buffer (pH 7), and extracted with DCM. The organic phase was dried over $MgSO_4$, filtered, and evaporated under reduced pressure. The residue was purified by silica flash chromatography (12% MeOH in DCM) to yield amide **22a-c** as a solid.

N-(2-{1-[(4-Amino-2-methylpyrimidin-5-yl)methyl]-1H-1,2,3-triazol-4-yl}ethyl)-2-methoxybenzamide **22a**

Prepared from **12a**. White solid (83 mg, 45%). m.p. 161-162 ºC. ^{1}H NMR (400 MHz, CD_3SOCD_3) δ 8.37 (br, 1H, NH), 7.97 (s, 1H), 7.93 (s, 1H), 7.77 (d, 1H, J = 7.5 Hz), 7.46 (m, 1H), 7.11 (d, 1H, J = 8.3 Hz), 7.02 (m, 1H), 5.41 (s, 2H), 4.04 (br, 2H, NH_2), 3.82 (s, 3H), 3.54 (m, 2H), 2.88 (t, 2H, J = 7.0 Hz), 2.31 (s, 3H). ^{13}C NMR (100 MHz, CD_3SOCD_3) δ 167.4, 165.2, 161.8, 157.5, 156.5, 145.2, 132.7, 131.0, 123.1, 122.9, 120.9, 112.4, 108.8, 56.1, 47.1, 39.3, 25.7, 25.7. HRMS (ESI) m/z: [M+H$^+$] calculated for $C_{18}H_{21}N_7O_2$: 368.1835; found: 368.1831.

N-(3-{1-[(4-Amino-2-methylpyrimidin-5-yl)methyl]-1H-1,2,3-triazol-4-yl}propyl)-2-methoxybenzamide **22b**

Prepared from **12b**. White solid (80 mg, 42%). m.p. 165-166 ºC. ^{1}H NMR (400 MHz, CD_3OD) δ 8.05 (s, 1H), 7.86 (d, 1H, J = 7.7 Hz), 7.83 (s, 1H), 7.50 (m, 1H), 7.13 (d, 1H, J = 8.4 Hz), 7.05 (m, 1H), 5.42 (s, 2H), 3.96 (s, 3H), 3.45 (t, 2H, J = 6.8 Hz), 2.80 (t, 2H, J = 7.5 Hz), 2.42 (s, 3H), 1.98 (m, 2H). ^{13}C NMR (100 MHz, CD_3OD) δ 167.5, 167.0, 162.2, 157.6, 155.2, 147.6, 132.5, 130.5, 122.1, 122.0, 120.5, 111.5, 108.5, 55.1, 47.2, 38.7, 28.7, 23.7, 22.3. HRMS (ESI) m/z: [M+H$^+$] calculated for $C_{19}H_{23}N_7O_2$: 382.1991; found: 382.1988.

N-(4-{1-[(4-Amino-2-methylpyrimidin-5-yl)methyl]-1H-1,2,3-triazol-4-yl}butyl)-2-methoxybenzamide **22c**

Prepared from **12c**. White solid (83 mg, 42%). m.p. 171-172 ºC. ^{1}H NMR (400 MHz, CD_3OD) δ 8.04 (s, 1H), 7.99 (s, 1H), 7.85 (d, 1H, J = 7.8 Hz), 7.48 (m, 1H), 7.12 (d, 1H, J = 8.3 Hz), 7.05 (m, 1H), 5.46 (s, 2H), 3.93 (s, 3H), 3.43 (t, 2H, J = 6.4 Hz), 2.76 (t, 2H, J = 7.5 Hz), 2.41 (s, 3H), 1.60-1.80 (m, 4H). ^{13}C NMR (100 MHz, CD_3OD) δ 167.6, 167.0, 162.1, 157.5, 155.0, 148.0, 132.7, 130.4, 122.1, 122.0, 120.5, 111.4, 108.6, 55.1, 47.2, 39.0, 28.5, 26.4, 24.5, 23.7. HRMS (ESI) m/z: [M+H$^+$] calculated for $C_{20}H_{25}N_7O_2$: 396.2148; found: 396.2155.

General procedure for preparation of 23a-c and *O*-Bn-24a-c:

To a stirred solution of corresponding alkynoic acid (2 mmol, 1 equiv.) in dry DMF and dry THF (1:4, 0.1 M) under nitrogen at 0 °C was added CDI (1.5 equiv.) slowly. The resultant mixture was stirred at r.t. for 1 h, treated with $NH_2OBn.HCl$ (2 equiv.), and stirred at r.t. overnight, then concentrated under reduced pressure, diluted with EtOAc (100 mL), washed with aqueous phosphate buffer (pH 7) (3 x 50 mL), dried over $MgSO_4$, filtered, and evaporated under reduced pressure. The residue was purified by silica flash chromatography (20% EtOAc in hexane). O-benzyl hydroxamate **23a-c** was co-eluted with the unreacted NH_2OBn and could be detected by ^{1}H NMR and LCMS in the mixture, which was subjected to the next step without further purification. To a stirred solution of the resultant mixture in *t*-BuOH and water (2:1, 0.33 M) was added $CuSO_4.5H_2O$ (0.03 equiv.), sodium ascorbate (0.3 equiv.), and azide **8** (1.2 equiv.). The resultant mixture was stirred at 40 °C for 2 days, then concentrated under reduced pressure, diluted with *n*-BuOH, washed with 0.1 M K_2CO_3, dried over $MgSO_4$, filtered, and evaporated under reduced pressure. The residue was purified by silica flash chromatography (10% MeOH in DCM) to yield the triazole (*O*-Bn-**24a-c**) as a solid.

N-(Benzyloxy)pent-4-ynamide **23a**

Prepared from 4-pentynoic acid. ^{1}H NMR (400 MHz, $CDCl_3$) δ 7.40-7.50 (m, 5H), 4.94 (s, 2H), 2.53 (m, 2H), 2.30 (m, 2H), 1.98 (t, 1H, J = 2.2 Hz).

3-{1-[(4-Amino-2-methylpyrimidin-5-yl)methyl]-1H-1,2,3-triazol-4-yl}-N-(benzyloxy)propanamide (*O*-Bn-**24a**)

Prepared from **23a**. White solid (183 mg, 25% yield over two steps). m.p. 177-178 ºC. ^{1}H NMR (400 MHz, CD_3OD) δ 8.05 (s, 1H), 7.77 (s, 1H), 7.30-7.38 (m, 5H), 5.46 (s, 2H), 4.71 (s, 2H), 3.00 (t, 2H, J = 6.6 Hz), 2.41 (t, 2H, J = 6.6 Hz), 2.34 (s, 3H). ^{13}C NMR (100 MHz, CD_3OD) δ 169.2, 167.3, 162.2, 155.5, 146.3, 135.4, 128.9, 128.5, 128.2, 122.3, 108.4, 77.6, 47.1, 31.7, 23.6, 20.6. ESI-MS m/z: [M+H$^+$] calculated for $C_{18}H_{21}N_7O_2$: 368.183; found: 368.18.

N-(Benzyloxy)hex-5-ynamide **23b**

Prepared from 5-hexynoic acid. ^{1}H NMR (400 MHz, $CDCl_3$) δ 7.40-7.48 (m, 5H), 4.92 (s, 2H), 2.26 (m, 2H), 2.21 (m, 2H), 1.97 (t, 1H, J = 2.3 Hz), 1.86 (m, 2H).

4-{1-[(4-Amino-2-methylpyrimidin-5-yl)methyl]-1H-1,2,3-triazol-4-yl}-N-(benzyloxy)butanamide (*O*-Bn-**24b**)

Prepared from **23b**. White solid (236 mg, 31% yield over two steps). m.p. 185-186 ºC. ^{1}H NMR (400 MHz, CD_3OD) δ 8.04 (s, 1H), 7.75 (s, 1H), 7.33-7.42 (m, 5H), 5.45 (s, 2H), 4.84 (s, 2H), 2.66 (t, 2H, J = 7.2 Hz), 2.42 (s, 3H), 2.10 (t, 2H, J = 7.5 Hz), 1.92 (m, 2H). ^{13}C NMR (100 MHz, CD_3OD) δ 170.1, 167.1, 162.2, 155.7, 147.3, 135.1, 128.9, 128.6, 128.3, 122.3, 108.1, 77.2, 47.1, 31.6, 24.8, 24.1, 23.6. ESI-MS m/z: [M+H$^+$] calculated for $C_{19}H_{23}N_7O_2$: 382.199; found: 382.20.

N-(Benzyloxy)hept-6-ynamide **23c**

Prepared from 6-heptynoic acid. ^{1}H NMR (400 MHz, $CDCl_3$) δ 7.41-7.49 (m, 5H), 4.95 (s, 2H), 2.24 (m, 2H), 2.10 (m, 2H), 1.95 (t, 1H, J = 2.2 Hz), 1.76 (m, 2H), 1.56 (m, 2H).

5-{1-[(4-Amino-2-methylpyrimidin-5-yl)methyl]-1H-1,2,3-triazol-4-yl}-N-(benzyloxy)pentanamide (*O*-Bn-**24c**)

Prepared from **23c**. White solid (237 mg, 30% yield over two steps). m.p. 190-191 ºC. ^{1}H NMR (400 MHz, CD_3OD) δ 8.05 (s, 1H), 7.76 (s, 1H), 7.30-7.35 (m, 5H), 5.46 (s, 2H), 4.83 (s, 2H), 2.68 (t, 2H, J = 6.6 Hz), 2.41 (s, 3H), 2.07 (t, 2H, J = 6.1 Hz), 1.62 (m, 4H). ^{13}C NMR (100 MHz, CD_3OD) δ 171.1, 167.5, 162.2, 155.0, 147.9, 135.6, 128.9, 128.2, 128.0, 121.9, 108.6, 77.5, 47.2, 31.9, 28.3, 24.5, 24.4, 23.6. ESI-MS m/z: [M+H$^+$] calculated for $C_{20}H_{25}N_7O_2$: 396.215; found: 396.22.

General procedures for preparation of 24a-c:

Method A: Debenzylation with BCl_3.

To a stirred solution of corresponding *O*-Bn-**24a-c** (0.2 mmol, 1 equiv.) in dry DCM (0.1 M) under nitrogen at -78 °C was added BCl_3 (1 M in DCM) (5 equiv.) dropwise. The resultant mixture was stirred at r.t. overnight. The reaction mixture was quenched with 1 M K_2CO_3 and extracted with *n*-BuOH. The organic phase was dried over $MgSO_4$, filtered, and evaporated under reduced pressure. The residue was purified by silica flash chromatography (15% MeOH in DCM) to yield hydroxamate **24a-c** as a solid.

Method B: Hydrogenation.

A stirred solution of corresponding *O*-Bn-**24b-c** (0.2 mmol, 1 equiv.) in MeOH (0.1 M) at r.t. was treated with 10% Pd/C (30 mg) under nitrogen. The flask was evacuated and flushed with hydrogen gas (three times). The resultant mixture was stirred vigorously at r.t. under an atmosphere of hydrogen (1 atm, H_2 balloon) for 4 h. The reaction mixture was filtered through Celite and concentrated under reduced pressure to yield hydroxamate **24b-c** as a solid.

3-{1-[(4-Amino-2-methylpyrimidin-5-yl)methyl]-1H-1,2,3-triazol-4-yl}-N-hydroxypropanamide **24a**

Prepared from *O*-Bn-**24a**. White solid (Method A: 14 mg, 25%). m.p. 222-223 °C. ^{1}H NMR (400 MHz, CD_3OD) δ 8.04 (s, 1H), 7.79 (s, 1H), 5.47 (s, 2H), 3.01 (t, 2H, *J* = 7.5 Hz), 2.46 (t, 2H, *J* = 7.5 Hz), 2.43 (s, 3H). ^{13}C NMR (100 MHz, CD_3OD) δ 170.2, 167.5, 162.2, 155.0, 146.7, 122.2, 108.5, 47.7, 31.8, 23.7, 20.9. HRMS (ESI) m/z: [M+H^+] calculated for $C_{11}H_{15}N_7O_2$: 278.1365; found: 278.1368.

4-{1-[(4-Amino-2-methylpyrimidin-5-yl)methyl]-1H-1,2,3-triazol-4-yl}-N-hydroxybutanamide **24b**

Prepared from *O*-Bn-**24b**. White solid (Method A: 11 mg, 19%; Method B: 55 mg, 95%). m.p. 232-233 °C. ^{1}H NMR (400 MHz, CD_3OD) δ 8.12 (s, 1H), 7.91 (s, 1H), 5.54 (s, 2H), 2.74 (t, 2H, *J* = 7.3 Hz), 2.48 (s, 3H), 2.18 (t, 2H, *J* = 7.0 Hz), 1.98 (m, 2H). ^{13}C NMR (100 MHz, CD_3OD) δ 170.1, 167.6, 162.2, 155.0, 147.5, 122.2, 108.5, 47.7, 31.6, 25.0, 24.2, 23.6. HRMS (ESI) m/z: [M+H^+] calculated for $C_{12}H_{17}N_7O_2$: 292.1522; found: 292.1531.

5-{1-[(4-Amino-2-methylpyrimidin-5-yl)methyl]-1H-1,2,3-triazol-4-yl}-N-hydroxypentanamide **24c**

Prepared from *O*-Bn-**19c**. White solid (Method A: 8 mg, 13%; Method B: 59 mg, 97%). m.p. 238-239 °C. ^{1}H NMR (400 MHz, CD_3OD) δ 8.06 (s, 1H), 7.85 (s, 1H), 5.52 (s, 2H), 2.72 (t, 2H, *J* = 7.5 Hz), 2.46 (s, 3H), 2.15 (t, 2H, *J* = 6.6 Hz), 1.67 (m, 4H). ^{13}C NMR (100 MHz, CD_3OD) δ 171.2, 166.6, 162.6, 152.7, 147.8, 122.2, 109.0, 46.9, 31.9, 28.3, 24.6, 24.4, 22.9. HRMS (ESI) m/z: [M+H^+] calculated for $C_{13}H_{19}N_7O_2$: 306.1678; found: 306.1688.

General procedure for preparation of 25a-c:

To a stirred solution of corresponding alkynoic acid (3 mmol, 1 equiv.) and DCC (3 equiv.) in dry DMF (0.2 M) under nitrogen at 0 °C was added DMAP (1.3 equiv.) and *N*-Boc-1,2-phenylenediamine (1.3 equiv.). The resultant mixture was stirred at r.t. for 2 days, then diluted with DCM, filtered through cotton wool (to remove DCC/DCU), washed with aqueous phosphate buffer (pH 7), and extracted with DCM. The organic phase was dried over $MgSO_4$, filtered, and evaporated under reduced pressure. The residue was purified by silica flash chromatography (20% EtOAc in hexane) to yield carbamate **25a-c** as a solid.

tert-Butyl N-[2-(pent-4-ynamido)phenyl]carbamate **25a**

Prepared from 4-pentynoic acid. Colourless oil (390 mg, 45%). ^{1}H NMR (400 MHz, CD_3OD) δ 7.61 (m, 1H), 7.39 (m, 1H), 7.22 (m, 1H), 7.14 (m, 1H), 2.56-2.68 (m, 4H), 2.34 (t, 1H, *J* = 2.5 Hz), 1.53 (s, 9H). ^{13}C NMR (100 MHz, CD_3OD) δ 171.7, 154.2, 132.0, 129.0, 126.1, 125.4, 124.2, 123.6, 82.1, 80.1, 69.0, 35.0, 27.0, 14.3. ESI-MS m/z: [M+H^+] calculated for $C_{16}H_{20}N_2O_3$: 289.155; found: 289.16.

tert-Butyl N-[2-(hex-5-ynamido)phenyl]carbamate **25b**

Prepared from 5-hexynoic acid. Colourless oil (453 mg, 50%). ^{1}H NMR (400 MHz, $CDCl_3$) δ 7.48 (m, 1H), 7.41 (m, 1H), 7.18 (m, 1H), 7.16 (m, 1H), 2.53 (t, 2H, *J* = 7.3 Hz), 2.34 (m, 2H), 2.03 (t, 1H, *J* = 2.5 Hz), 1.97 (m, 2H), 1.54 (s, 9H). ^{13}C NMR (100 MHz, $CDCl_3$) δ 171.5, 154.3, 130.5, 129.8, 126.3, 125.4, 125.3, 124.6, 83.4, 80.9, 69.4, 35.7, 28.3, 24.1, 17.8. ESI-MS m/z: [M+H^+] calculated for $C_{17}H_{22}N_2O_3$: 303.171; found: 303.17.

tert-Butyl N-[2-(hept-6-ynamido)phenyl]carbamate **25c**

Prepared from 6-heptynoic acid. Colourless oil (730 mg, 77% with some impurities). ^{1}H NMR (400 MHz, $CDCl_3$) δ 7.45 (m, 1H), 7.39 (m, 1H), 7.16 (m, 2H), 2.40 (t, 2H, *J* = 7.4 Hz), 2.25 (m, 2H), 2.00 (t, 1H, *J* = 2.5 Hz), 1.86 (m, 2H), 1.54 (s, 9H), 1.63 (m, 2H). ESI-MS m/z: [M+H^+] calculated for $C_{18}H_{24}N_2O_3$: 317.186; found: 317.18. Not entirely pure; full purification and characterisation was conducted after the next step.

General procedure for preparation of Boc-26a-c:

To a stirred solution of corresponding alkyne **25a-c** (1 equiv.) and azide **8** (1.5 equiv.) in *t*-BuOH and water (2:1, 0.2 M) was added $CuSO_4.5H_2O$ (0.03 equiv.) and sodium ascorbate (0.3 equiv.). The resultant mixture was stirred at r.t. for 2 days, then concentrated under reduced pressure, diluted with EtOAc, washed with 1 M K_2CO_3, dried over $MgSO_4$, filtered, and evaporated under reduced pressure. The residue was purified by silica flash chromatography (10% MeOH in DCM) to yield triazole Boc-**26a-c** as a solid.

tert-Butyl N-[2-(3-{1-[(4-amino-2-methylpyrimidin-5-yl)methyl]-1H-1,2,3-triazol-4-yl}propanamido)phenyl]carbamate (Boc-**26a**)

Prepared from **25a** (390 mg, 1.35 mmol). White solid (213 mg, 35%). m.p. 180-181 ºC. ^{1}H NMR (400 MHz, CD_3OD) δ 8.05 (s, 1H), 7.80 (s, 1H), 7.55 (m, 1H), 7.32 (m, 1H), 7.20 (m, 1H), 7.11 (m, 1H), 5.47 (s, 2H), 3.10 (t, 2H, J = 7.2 Hz), 2.79 (t, 2H, J = 7.2 Hz), 2.42 (s, 3H), 1.50 (s, 9H). ^{13}C NMR (100 MHz, CD_3OD) δ 172.2, 167.5, 162.2, 154.5, 154.4, 147.1, 131.8, 129.4, 125.9, 125.2, 124.3, 123.9, 122.3, 108.6, 80.0, 47.1, 35.4, 27.1, 23.4, 21.1. ESI-MS m/z: [M+H$^+$] calculated for $C_{22}H_{28}N_8O_3$: 453.236; found: 453.23.

tert-Butyl N-[2-(4-{1-[(4-amino-2-methylpyrimidin-5-yl)methyl]-1H-1,2,3-triazol-4-yl}butanamido)phenyl]carbamate (Boc-**26b**)

Prepared from **25b** (453 mg, 1.5 mmol). Brown solid (280 mg, 40%). m.p. 191-192 ºC. ^{1}H NMR (400 MHz, CD_3OD) δ 8.05 (s, 1H), 7.82 (s, 1H), 7.53 (m, 1H), 7.37 (m, 1H), 7.20 (m, 1H), 7.15 (m, 1H), 5.46 (s, 2H), 2.81 (t, 2H, J = 7.6 Hz), 2.48 (t, 2H, J = 7.4 Hz), 2.42 (s, 3H), 2.06 (m, 2H), 1.46 (s, 9H). ^{13}C NMR (100 MHz, CD_3OD) δ 173.2, 167.6, 162.2, 155.1, 154.4, 147.5, 131.8, 129.7, 125.9, 125.2, 124.4, 124.1, 122.1, 108.6, 80.0, 47.2, 35.3, 27.3, 25.1, 24.3, 23.7. ESI-MS m/z: [M+H$^+$] calculated for $C_{23}H_{30}N_8O_3$: 467.152; found: 467.15.

tert-Butyl N-[2-(5-{1-[(4-amino-2-methylpyrimidin-5-yl)methyl]-1H-1,2,3-triazol-4-yl}pentanamido)phenyl]carbamate (Boc-**26c**)

Prepared from **25c** (730 mg, 2.31 mmol with impurities). White solid (310 mg, 28%). m.p. 199-200 ºC. ^{1}H NMR (400 MHz, CD_3OD) δ 8.05 (s, 1H), 7.79 (s, 1H), 7.53 (m, 1H), 7.37 (m, 1H), 7.21 (m, 1H), 7.14 (m, 1H), 5.47 (s, 2H), 2.77 (t, 2H, J = 7.0 Hz), 2.47 (t, 2H, J = 6.3 Hz), 2.41 (s, 3H), 1.77 (m, 4H), 1.47 (s, 9H). ^{13}C NMR (100 MHz, CD_3OD) δ 173.5, 167.6, 162.1, 155.0, 154.4, 147.8, 131.8, 129.7, 125.9, 125.2, 124.5, 124.2, 121.9, 108.6, 80.0, 47.1, 35.7, 28.3, 27.2, 24.8, 24.6, 23.7. ESI-MS m/z: [M+H$^+$] calculated for $C_{24}H_{32}N_8O_3$: 481.268; found: 481.26.

General procedure for preparation of 26a-c:

To a stirred solution of corresponding Boc-protected aniline Boc-**26a-c** (0.2 mmol, 1 equiv.) in dry DCM (0.1 M) under nitrogen at 0 °C was added TFA (10 equiv.) dropwise. The resultant mixture was stirred at r.t. for 4 h and then treated with Amberlyst A-21 resin (1 g) and MeOH (3 mL). The reaction mixture was stirred at r.t. for 3 h, diluted with MeOH, filtered through cotton wool (to remove the resin), and concentrated under reduced pressure. The residue was purified by silica flash chromatography (15% MeOH in DCM) to yield aniline **26a-c** as a solid.

3-{1-[(4-Amino-2-methylpyrimidin-5-yl)methyl]-1H-1,2,3-triazol-4-yl}-N-(2-aminophenyl)propanamide **26a**

Prepared from Boc-**26a**. White solid (39 mg, 55%). m.p. 212-213 ºC. ^{1}H NMR (400 MHz, CD_3OD) δ 8.06 (s, 1H), 7.81 (s, 1H), 7.02 (m, 1H), 7.00 (m, 1H), 6.82 (m, 1H), 6.68 (m, 1H), 5.46 (s, 2H), 3.10 (t, 2H, J = 7.2 Hz), 2.78 (t, 2H, J = 7.2 Hz), 2.41 (s, 3H). ^{13}C NMR (100 MHz, CD_3OD) δ 172.2, 167.5, 162.2, 155.2, 147.1, 142.2, 127.0, 126.0, 123.5, 122.4, 118.0, 116.9, 108.4, 47.3, 34.9, 23.7, 21.0. HRMS (ESI) m/z: [M+H$^+$] calculated for $C_{17}H_{20}N_8O$: 353.1838; found: 353.1849.

4-{1-[(4-Amino-2-methylpyrimidin-5-yl)methyl]-1H-1,2,3-triazol-4-yl}-N-(2-aminophenyl)butanamide **26b**

Prepared from Boc-**26b**. White solid (27 mg, 36%). m.p. 222-223 °C. ^{1}H NMR (400 MHz, CD_3OD) δ 8.05 (s, 1H), 7.84 (s, 1H), 7.09 (m, 1H), 7.03 (m, 1H), 6.84 (m, 1H), 6.71 (m, 1H), 5.47 (s, 2H), 2.82 (t, 2H, J = 7.4 Hz), 2.48 (t, 2H, J = 7.4 Hz), 2.43 (s, 3H), 2.07 (q, 2H, J = 7.4 Hz). ^{13}C NMR (100 MHz, CD_3OD) δ 171.2, 167.2, 162.2, 154.2, 147.6, 142.1, 128.7, 126.7, 125.5, 123.4, 118.0, 117.1, 108.6, 47.3, 34.9, 25.2, 24.3, 23.4. HRMS (ESI) m/z: [$M+H^+$] calculated for $C_{18}H_{22}N_8O$: 367.1995; found: 367.1985.

5-{1-[(4-Amino-2-methylpyrimidin-5-yl)methyl]-1H-1,2,3-triazol-4-yl}-N-(2-aminophenyl)pentanamide **26c**

Prepared from Boc-**26c**. White solid (31 mg, 40%). m.p. 228-229 °C. ^{1}H NMR (400 MHz, CD_3OD) δ 8.04 (s, 1H), 7.79 (s, 1H), 7.08 (m, 1H), 7.03 (m, 1H), 6.85 (m, 1H), 6.71 (m, 1H), 5.45 (s, 2H), 2.77 (m, 2H), 2.45 (m, 2H), 2.42 (s, 3H), 1.77 (m, 4H). ^{13}C NMR (100 MHz, CD_3OD) δ 173.4, 167.5, 162.1, 154.9, 148.0, 141.9, 126.9, 125.7, 123.7, 121.9, 118.1, 117.1, 108.6, 47.3, 35.4, 28.6, 25.0, 24.6, 23.6. HRMS (ESI) m/z: [$M+H^+$] calculated for $C_{19}H_{24}N_8O$: 381.2151; found: 381.2155.

NMR spectra

^{1}H NMR of **9b** in CD_3OD:

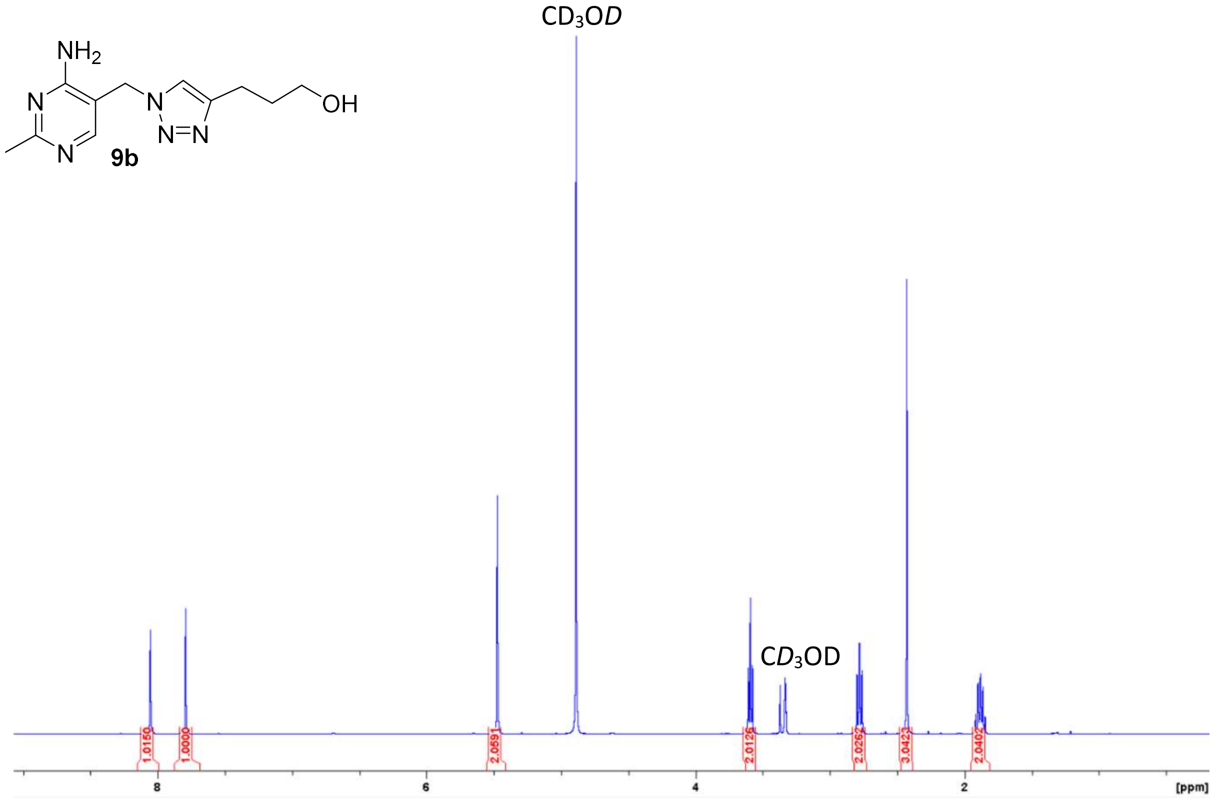

^{13}C NMR of **9b** in CD_3OD:

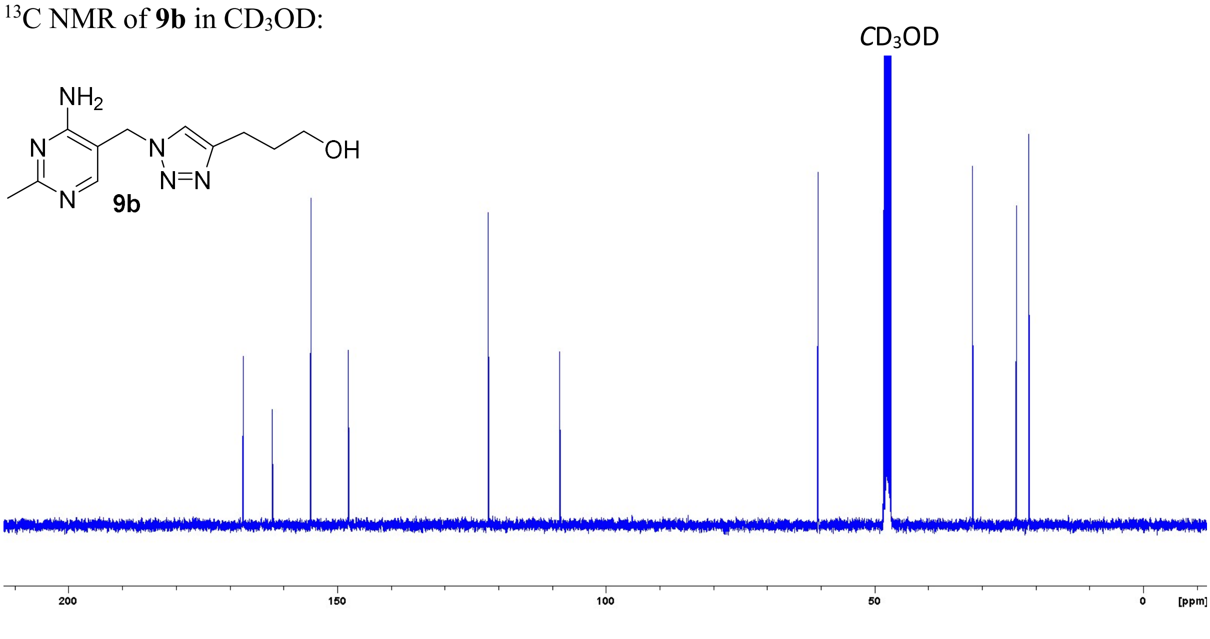

^{1}H NMR of **9c** in CD_3OD:

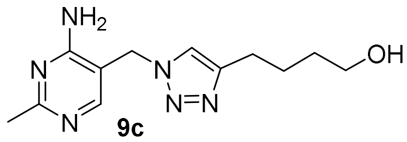

9c

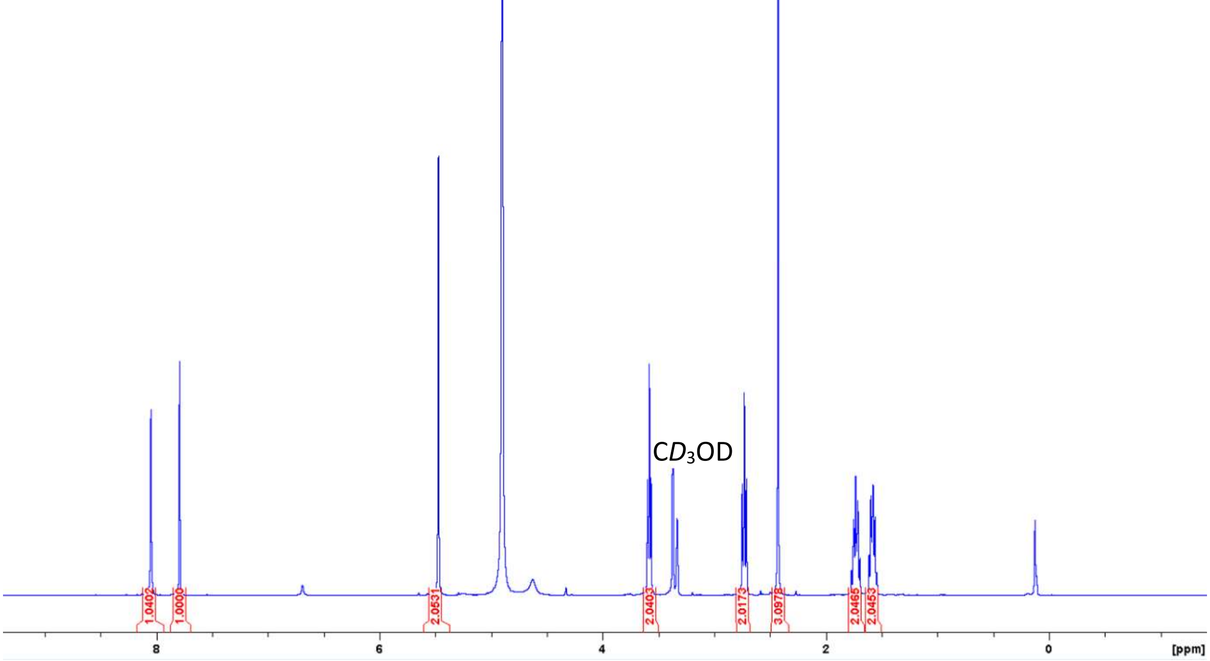

^{13}C NMR of **9c** in CD_3OD:

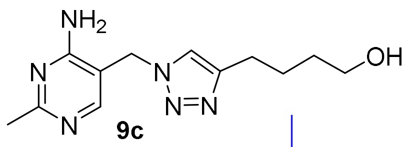

9c

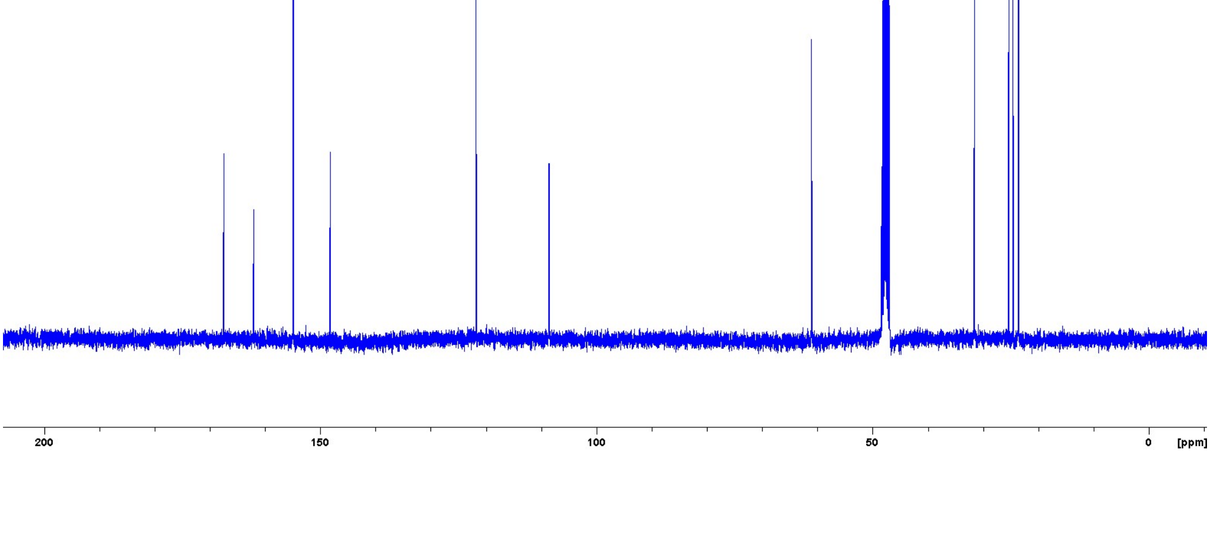

^{1}H NMR of **10b** in CD_3OD:

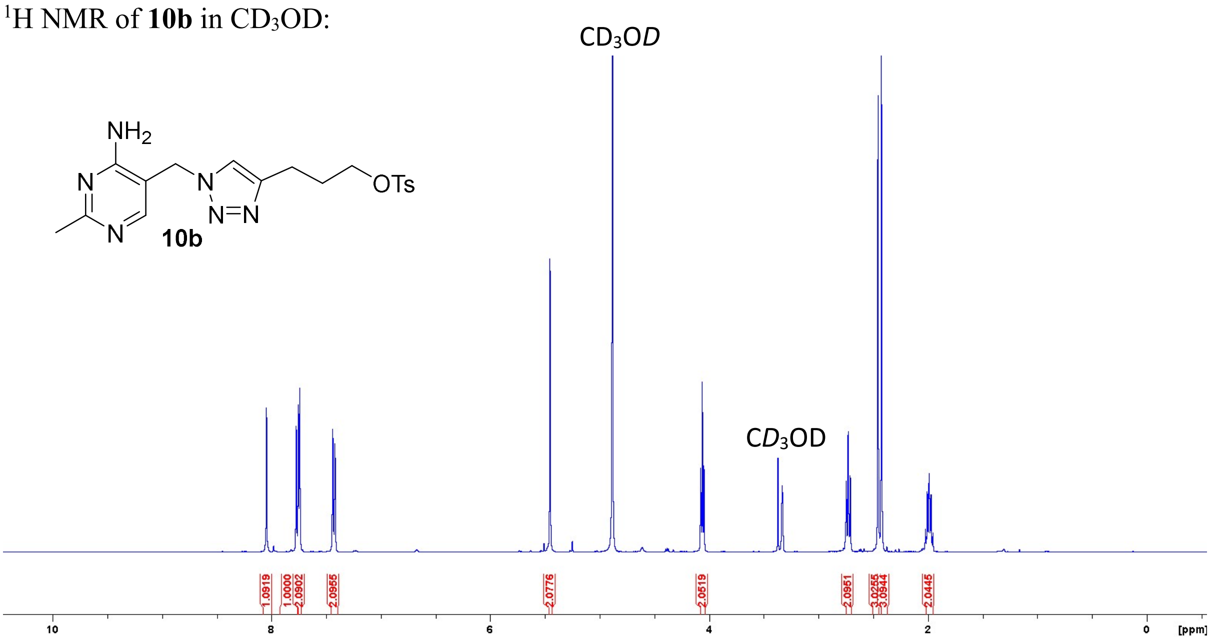

^{1}H NMR of **10c** in CD_3OD:

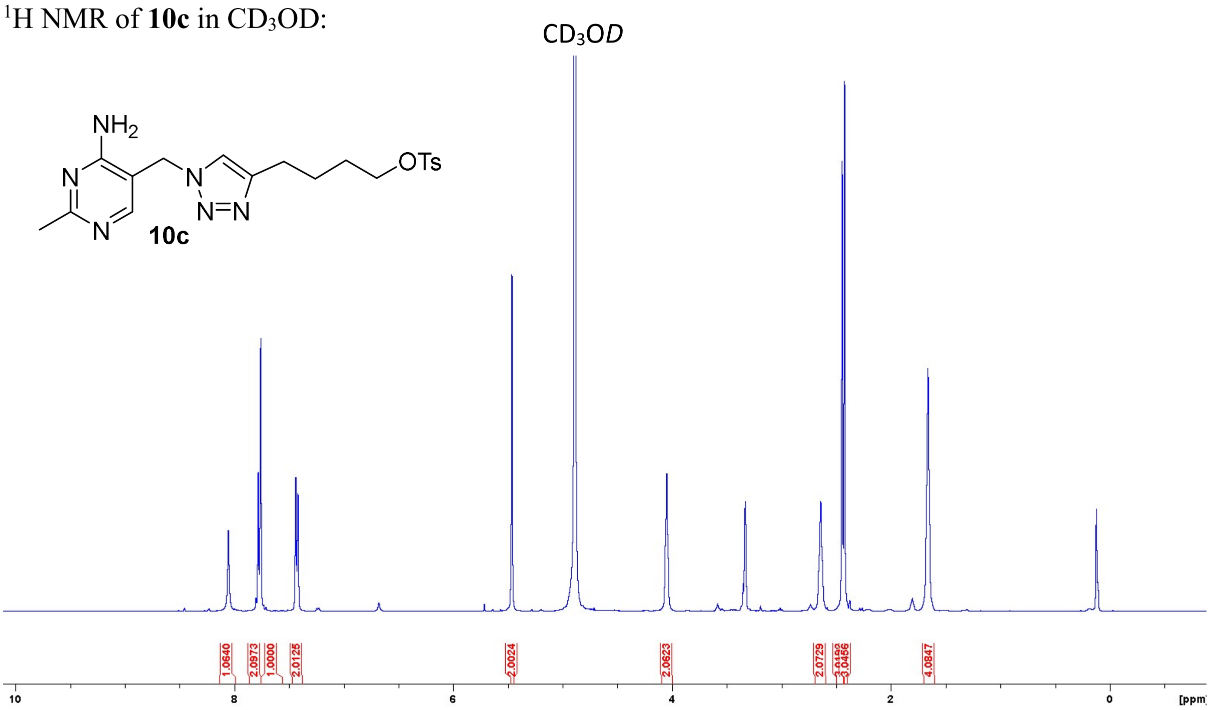

^{1}H NMR of **11b** in CD_3OD:

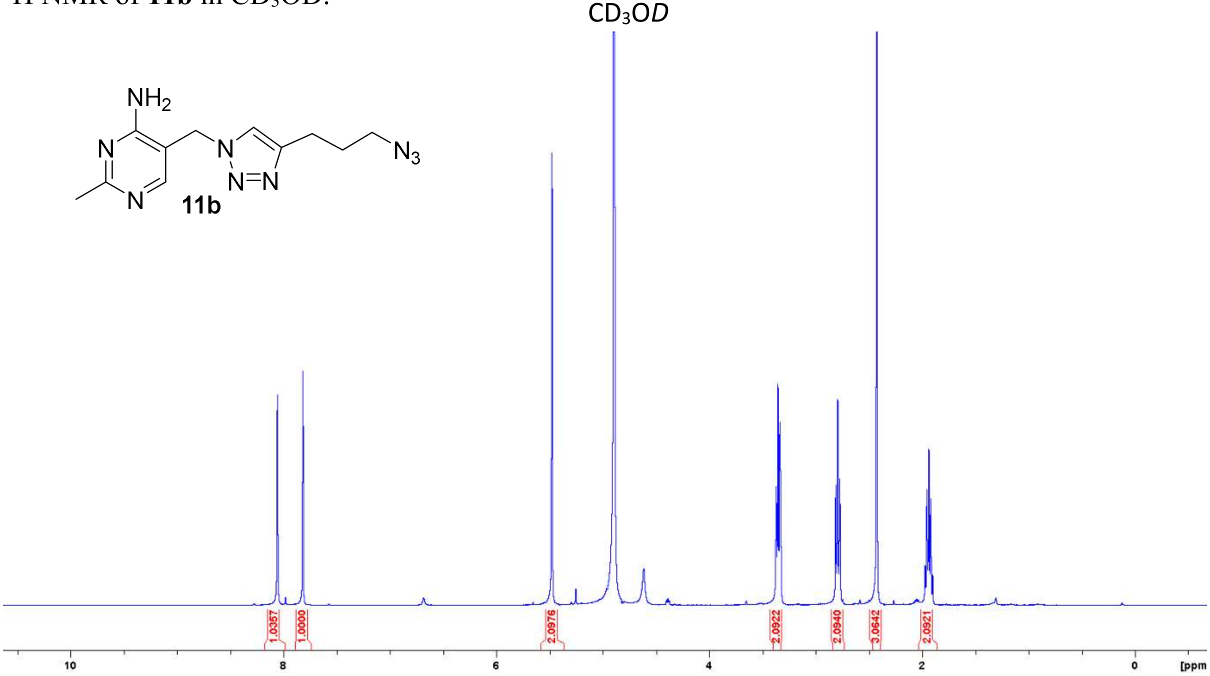

^{13}C NMR of **11b** in CD_3OD:

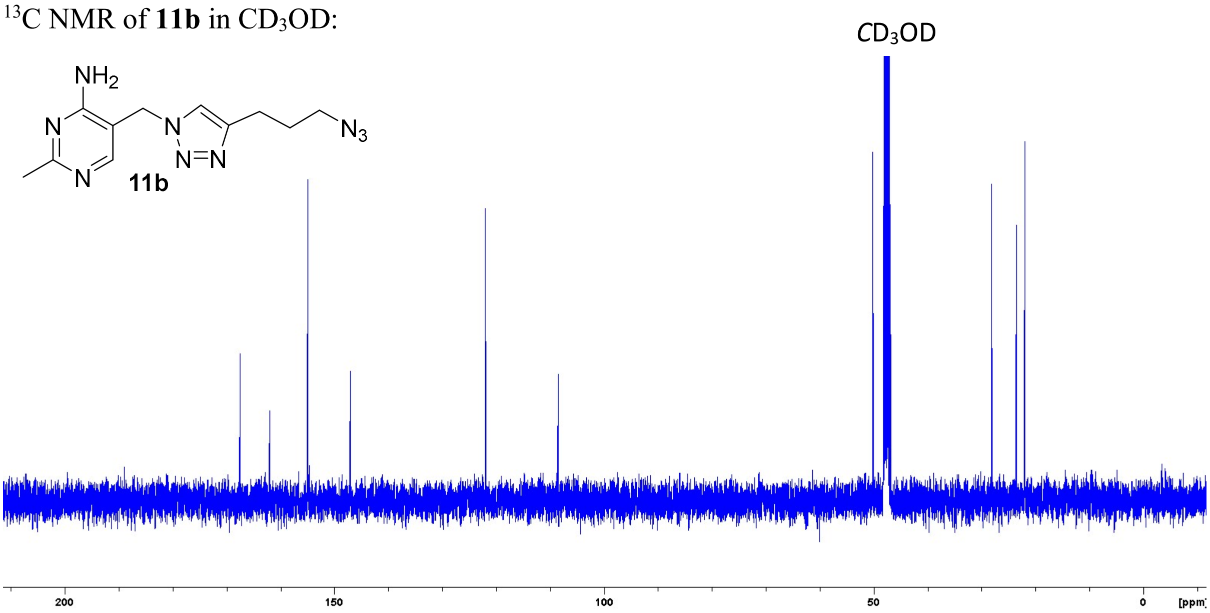

^{1}H NMR of **11c** in CD_3OD:

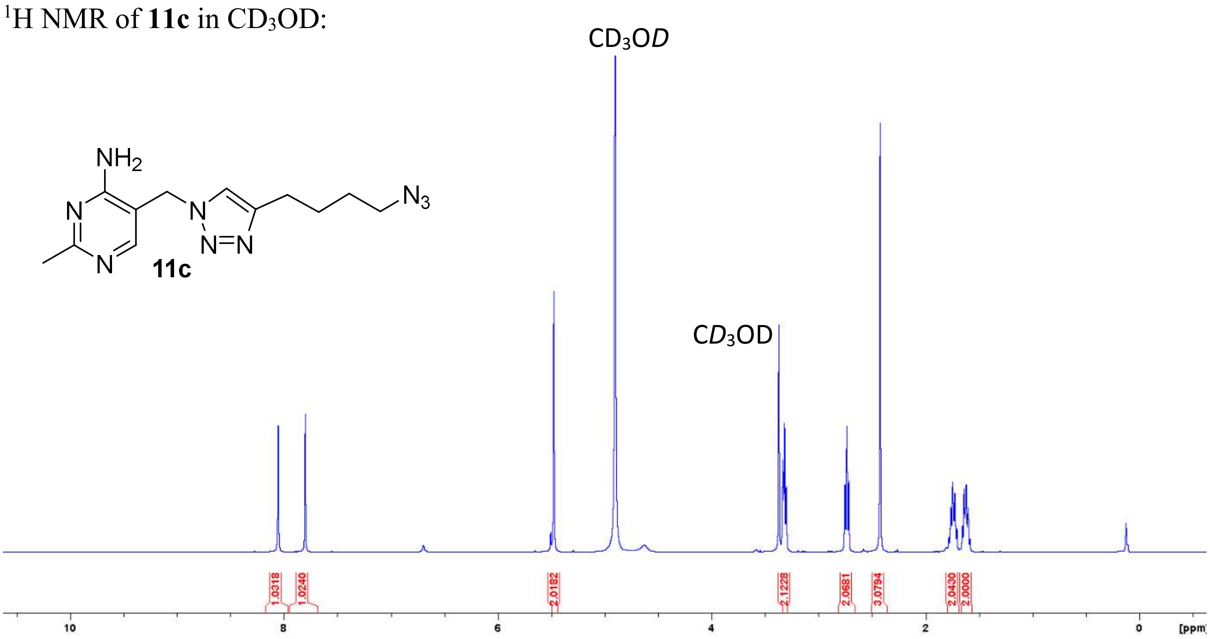

^{13}C NMR of **11c** in CD_3OD:

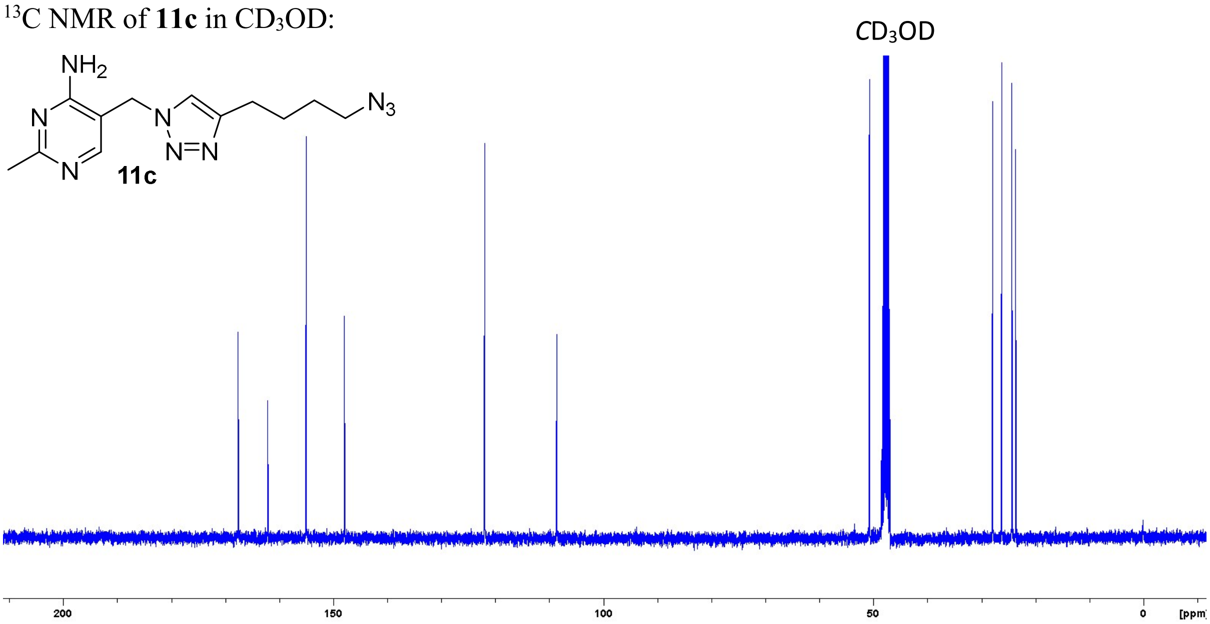

^{1}H NMR of **12b** in CD_3OD:

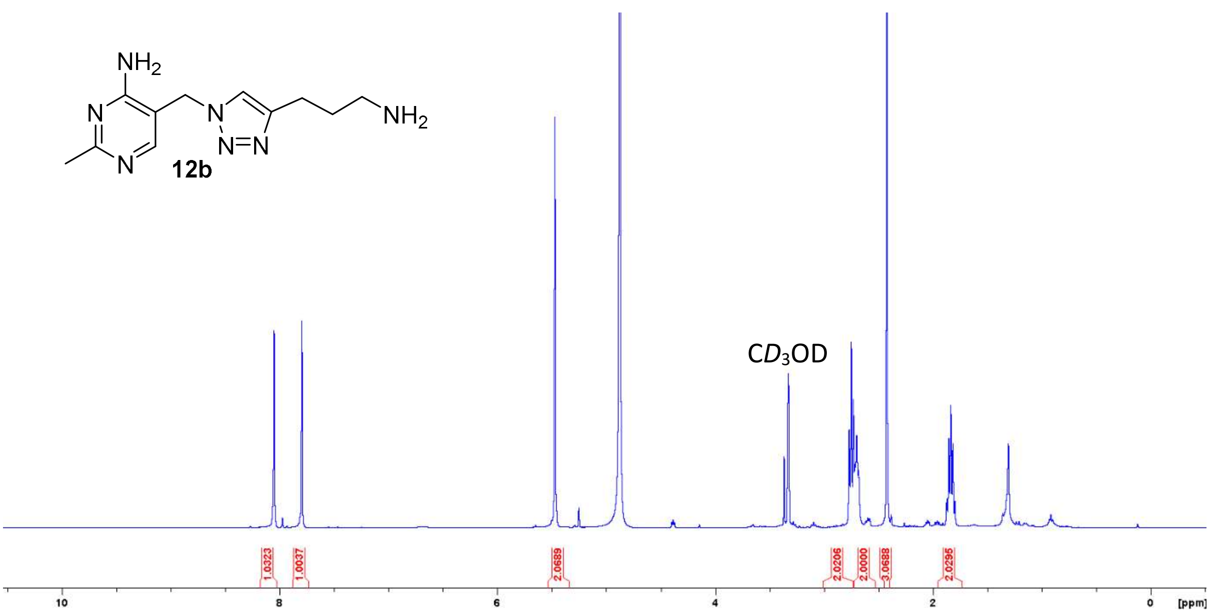

^{13}C NMR of **12b** in CD_3OD:

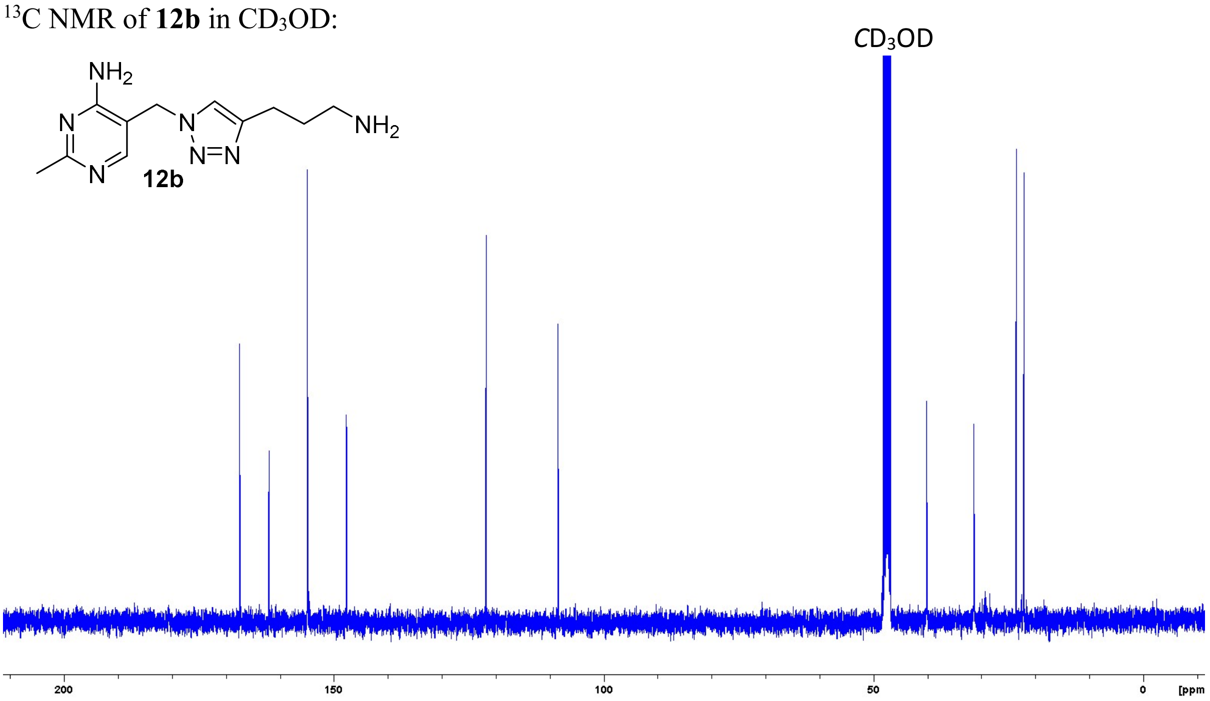

^{1}H NMR of **12c** in CD_3OD:

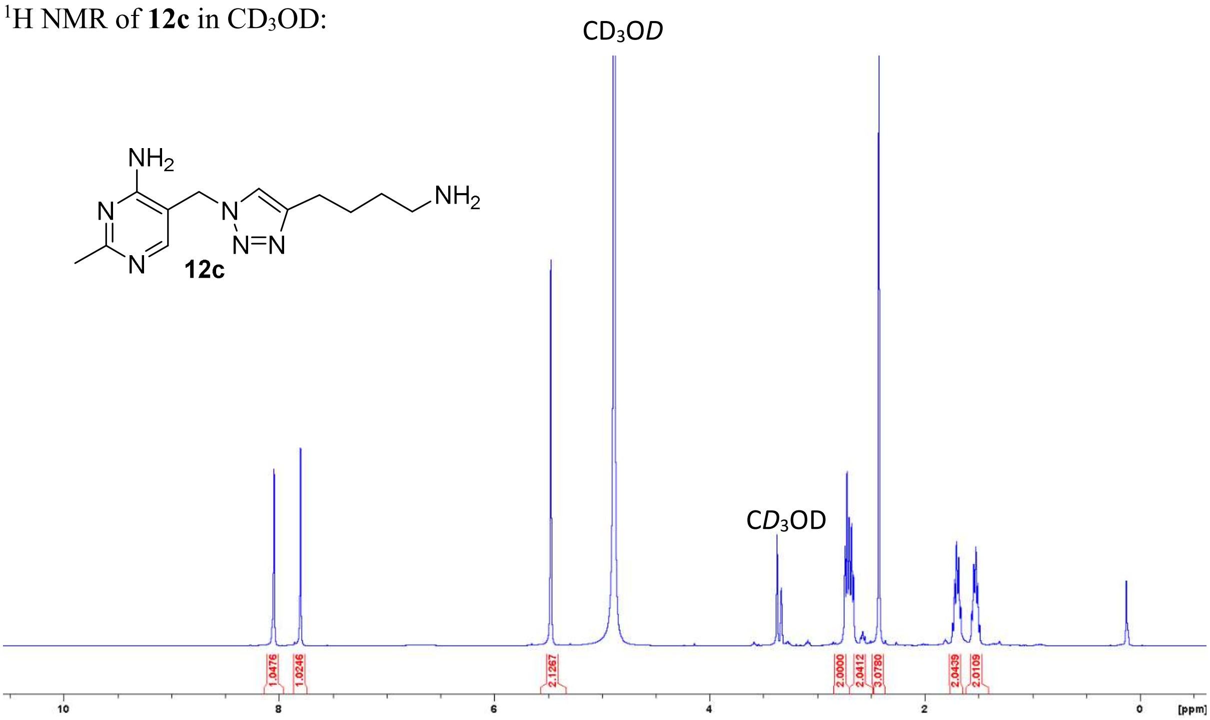

^{13}C NMR of **12c** in CD_3OD:

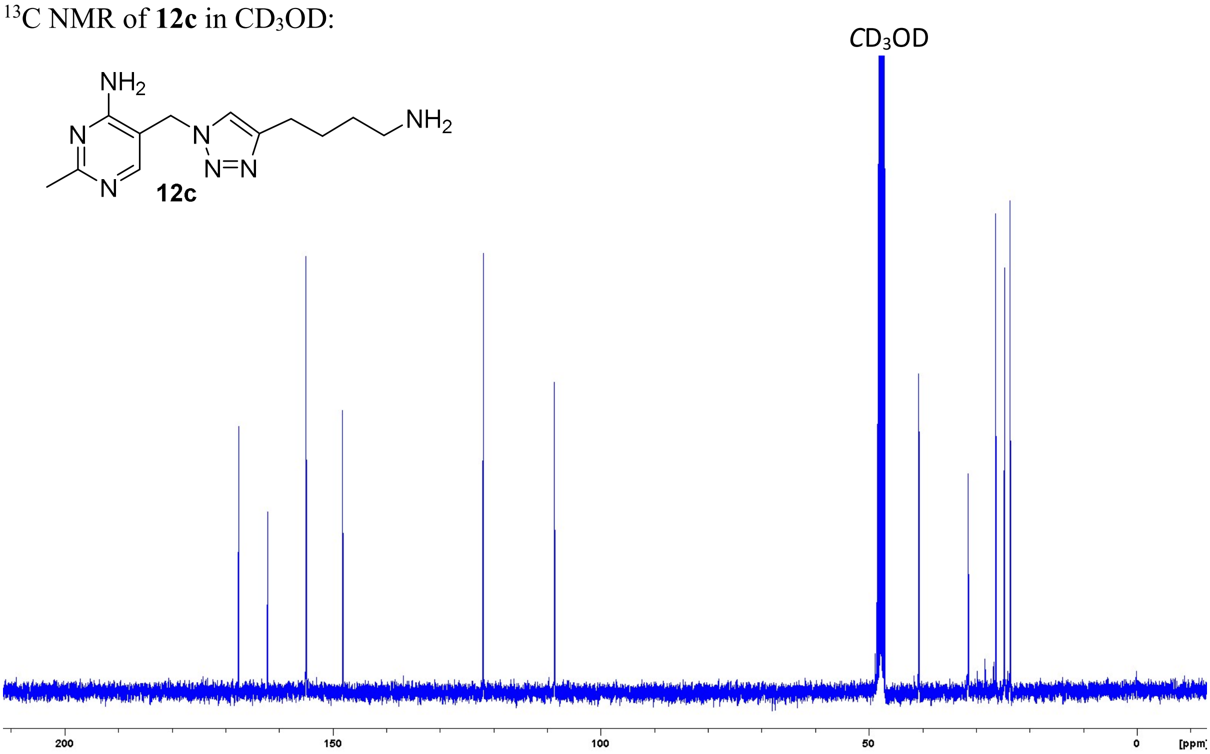

^{1}H NMR of **13a** in CD_3OD:

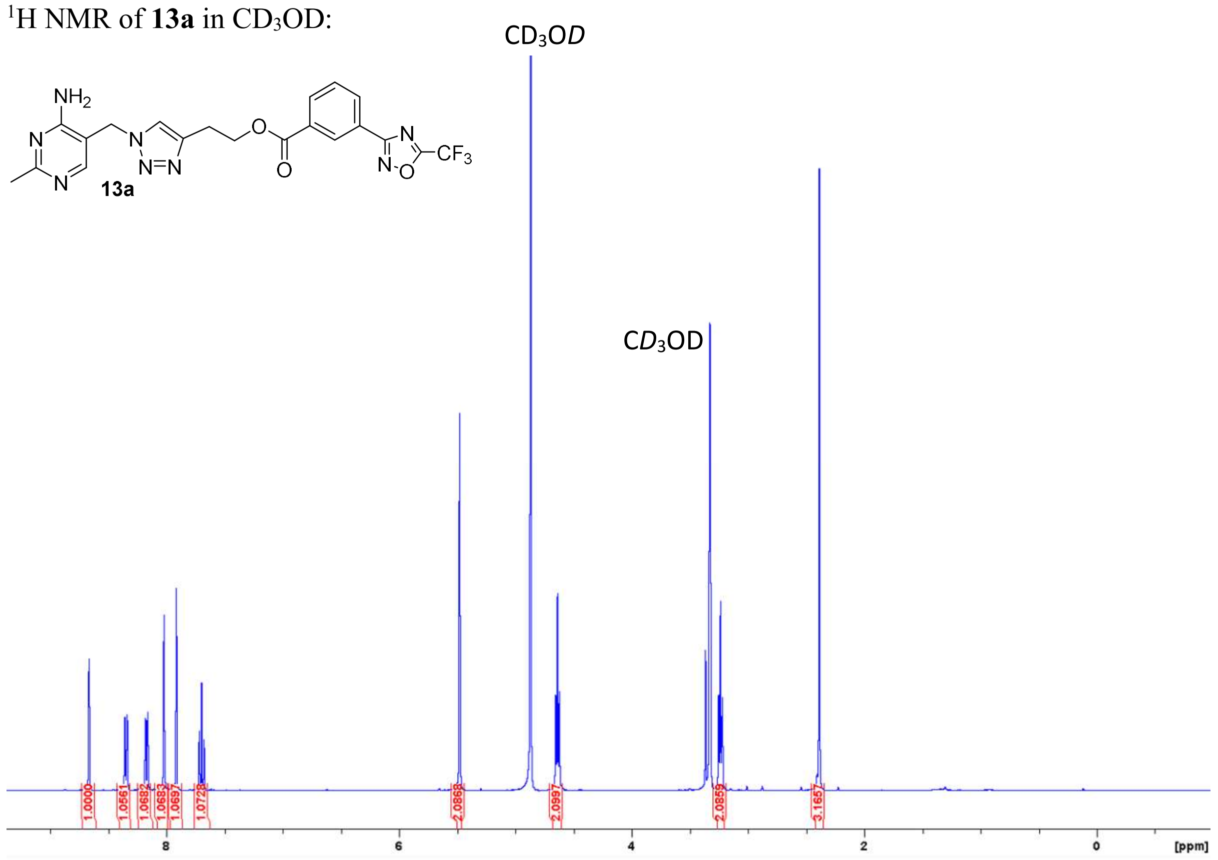

^{13}C NMR of **13a** in CD_3OD:

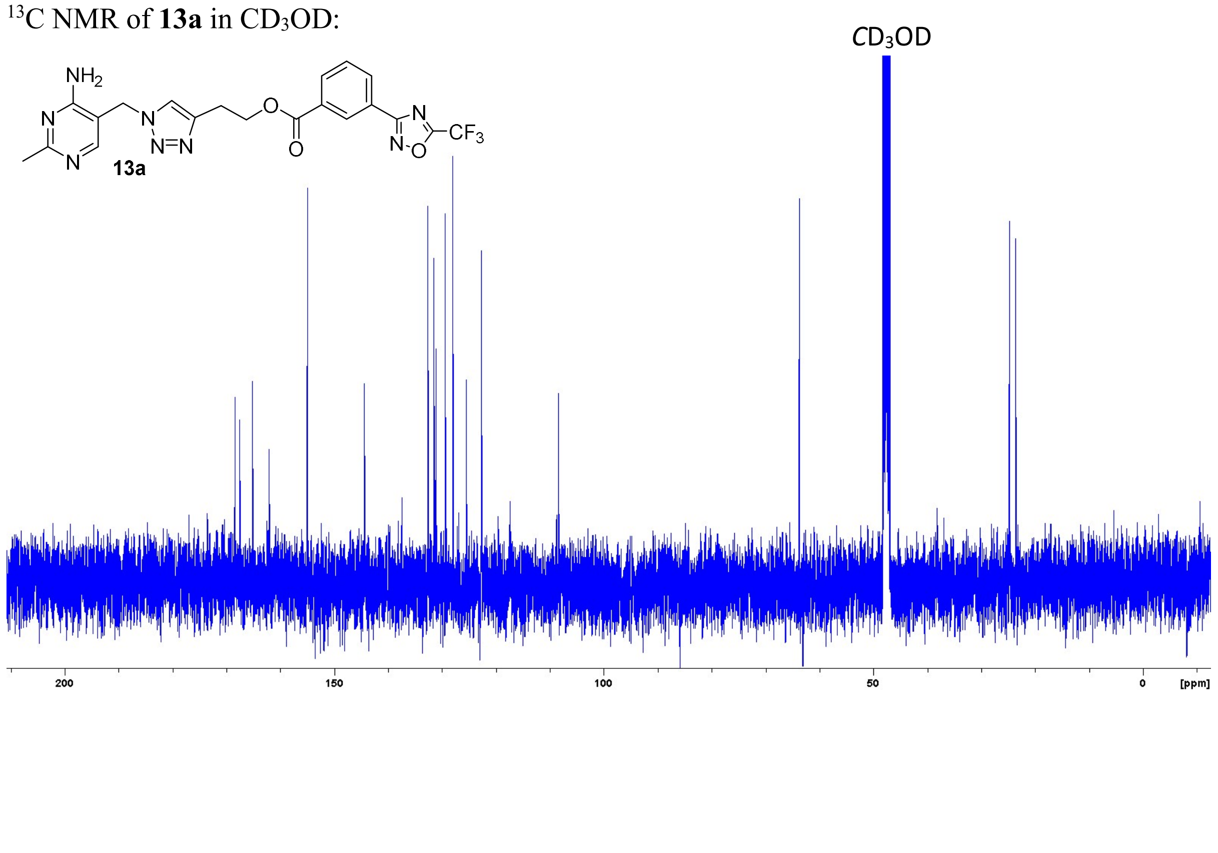

^{13}C DEPT-135 NMR of **13a** in CD_3OD:

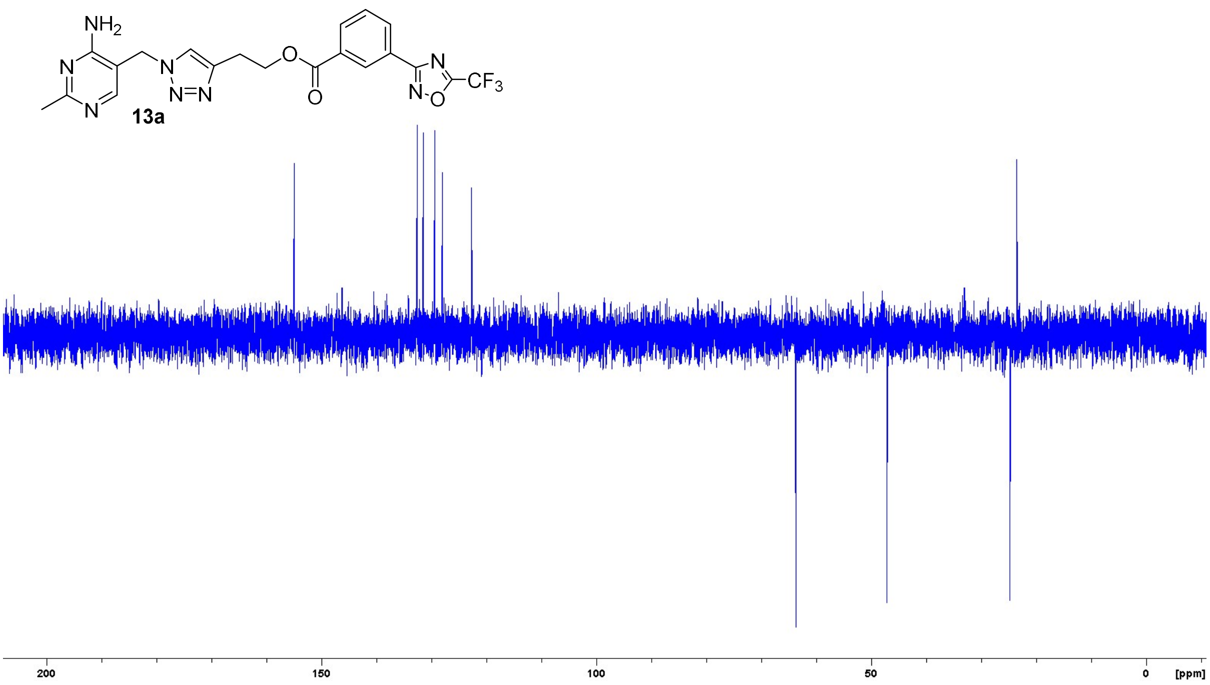

^{1}H NMR of **14a** in CD_3OD:

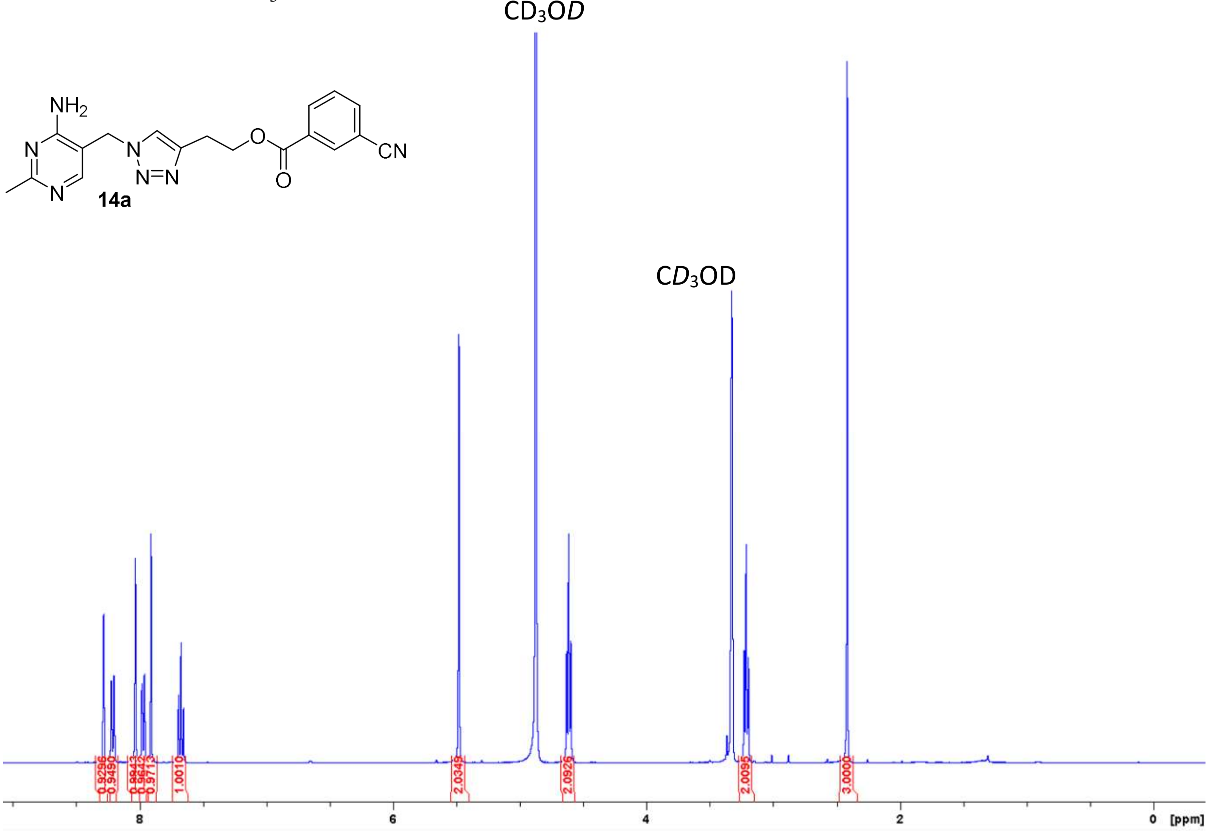

^{13}C NMR of **14a** in CD_3OD:

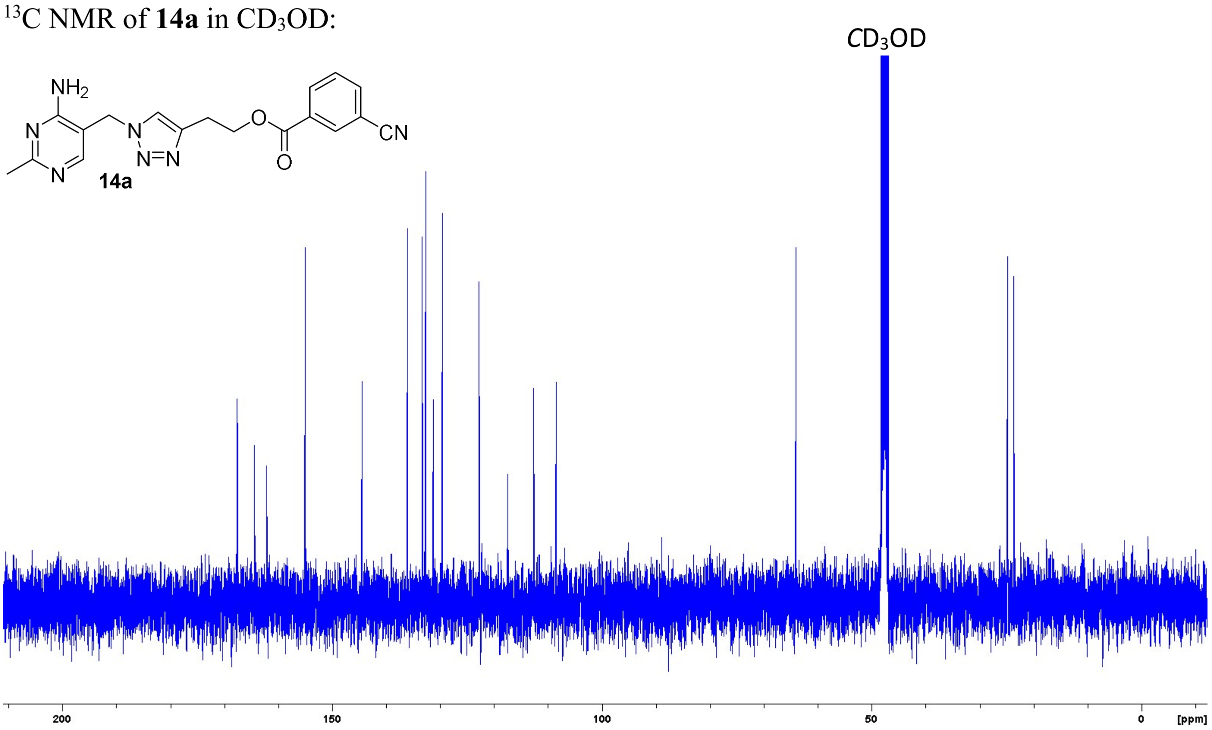

^{13}C DEPT-135 NMR of **14a** in CD_3OD:

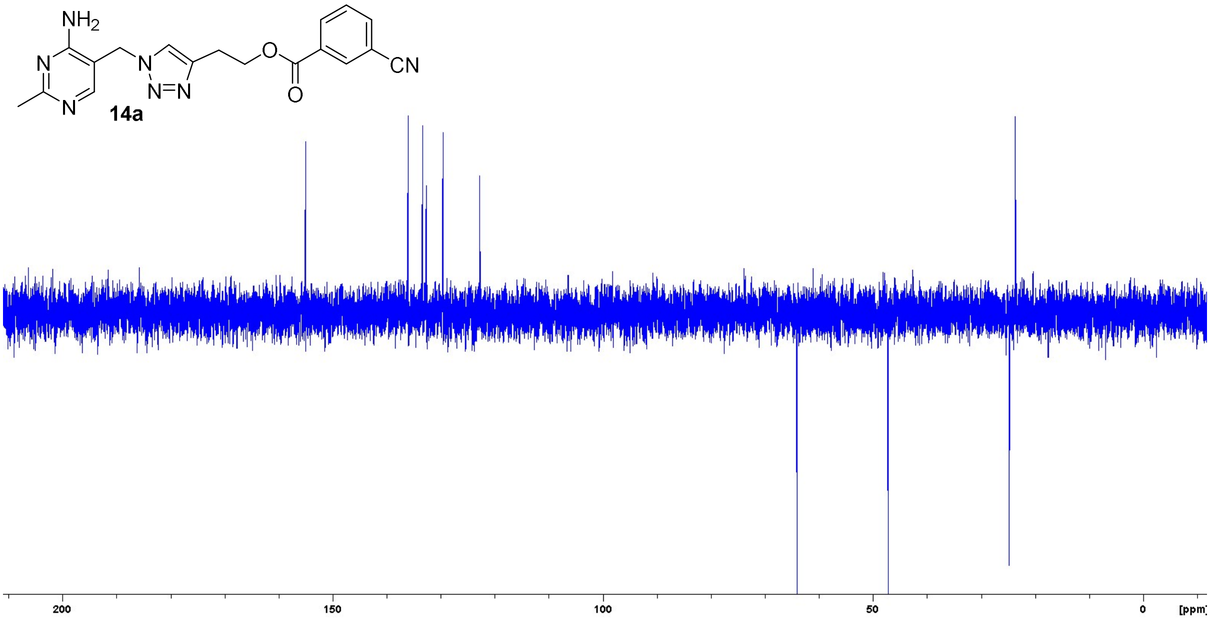

^{1}H NMR of **15a** in CD_3SOCD_3:

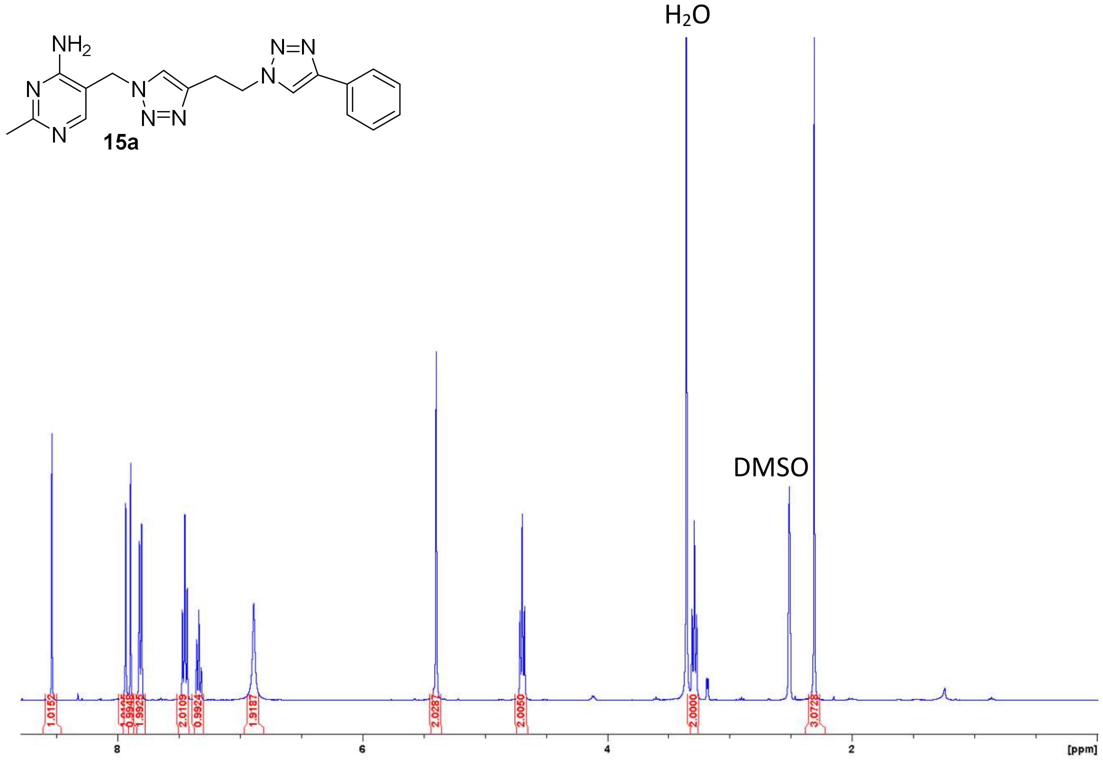

^{13}C NMR of **15a** in CD_3SOCD_3:

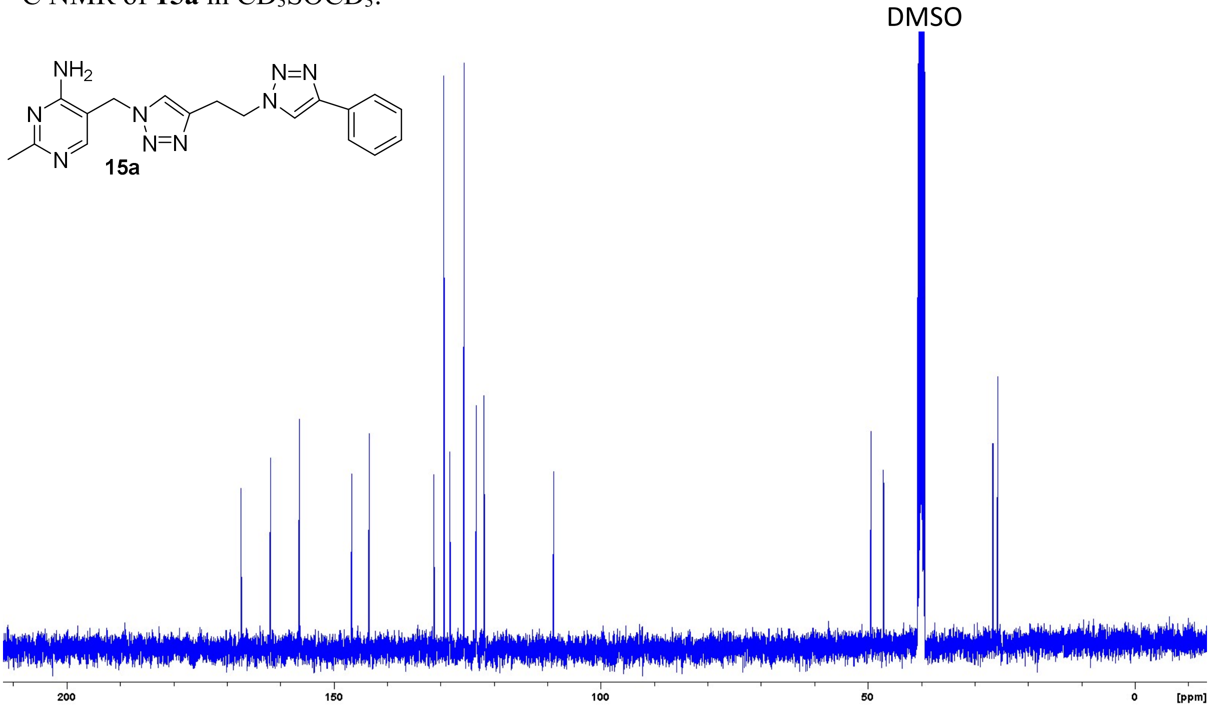

^{1}H NMR of **15b** in CD_3OD:

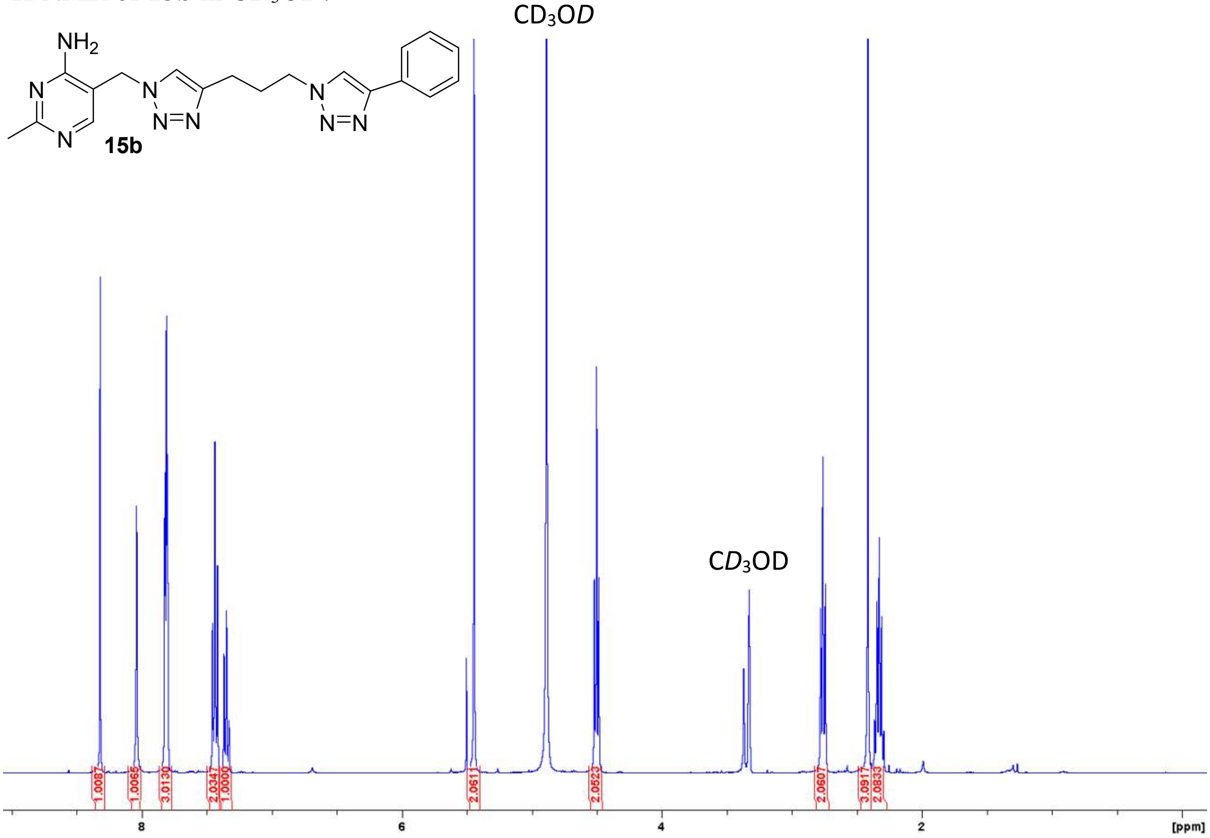

^{13}C NMR of **15b** in CD_3OD:

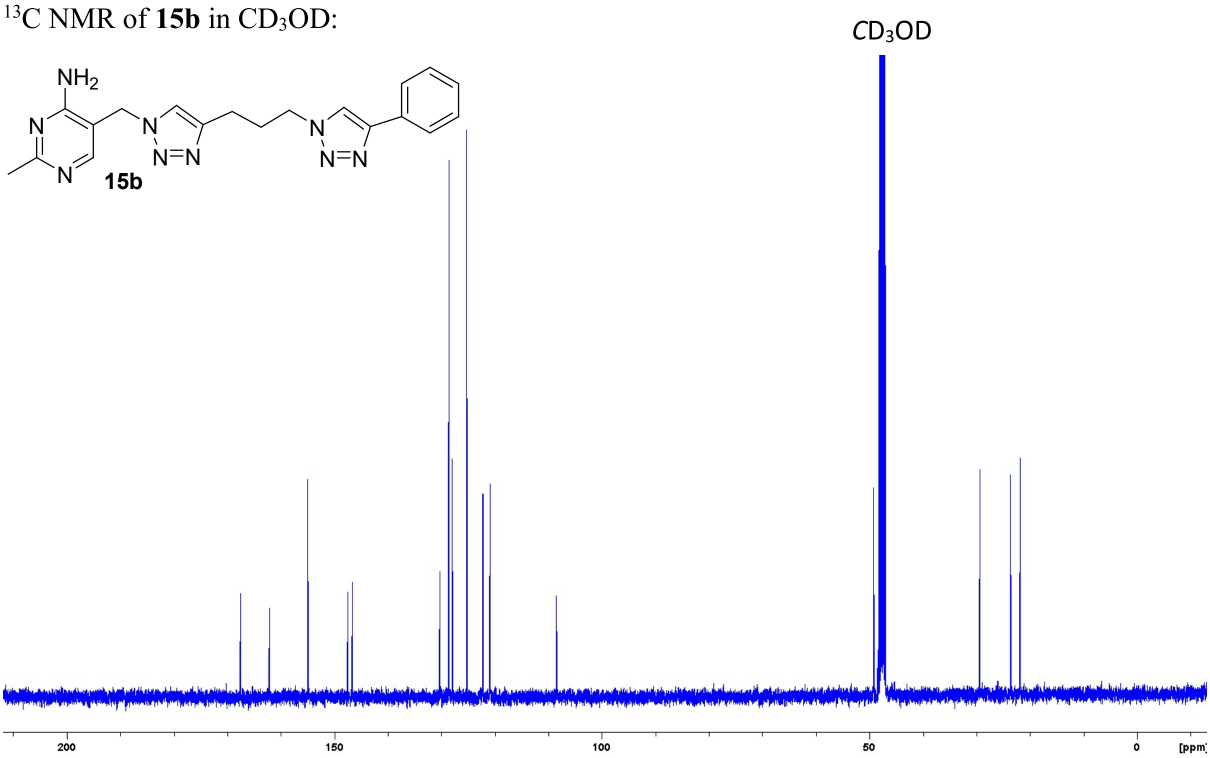

^{1}H NMR of **15c** in CD_3SOCD_3:

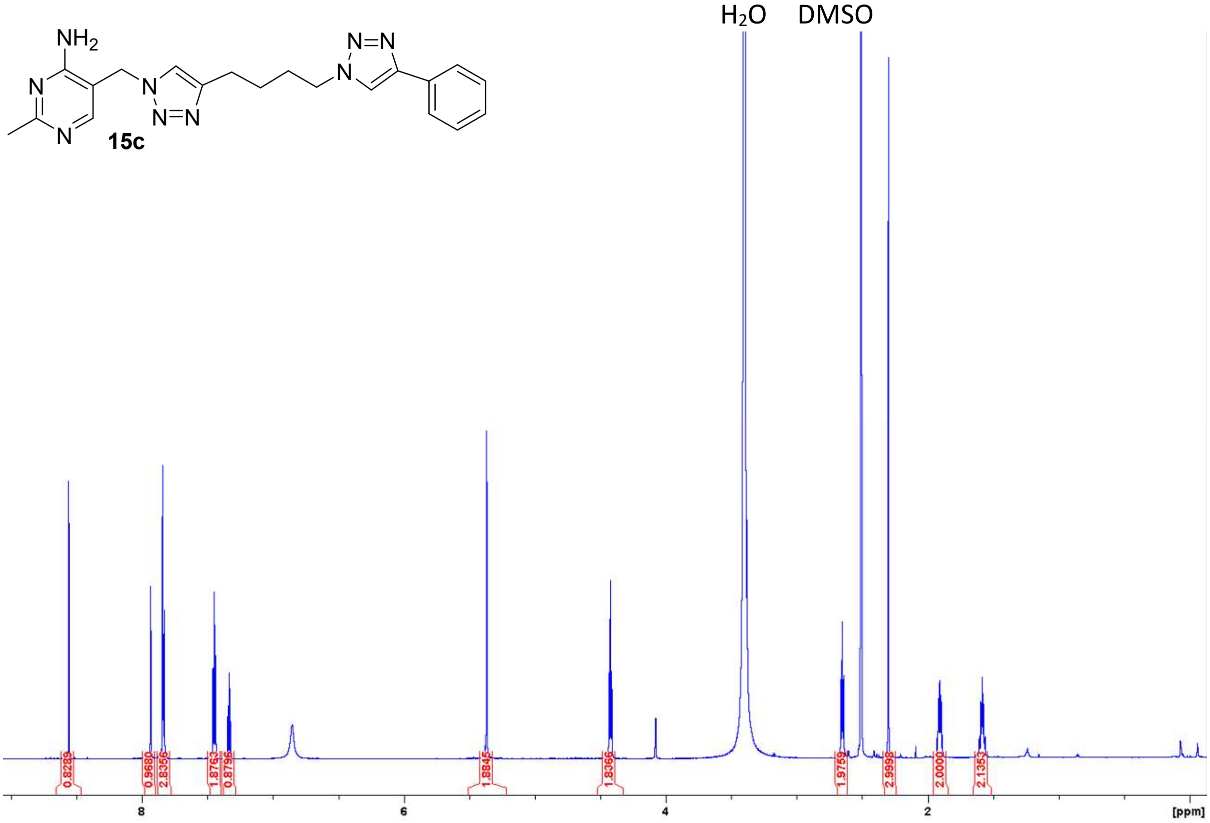

^{13}C NMR of **15c** in CD_3SOCD_3:

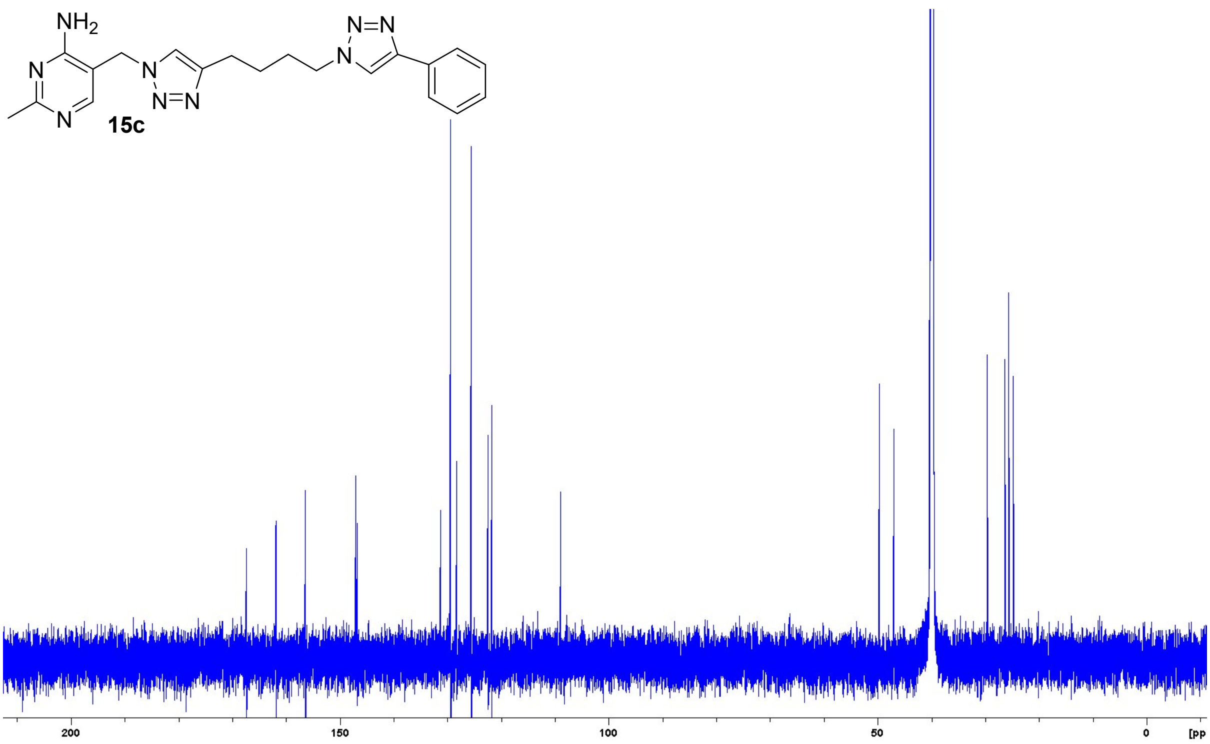

^{1}H NMR of **16a** in CD_3SOCD_3:

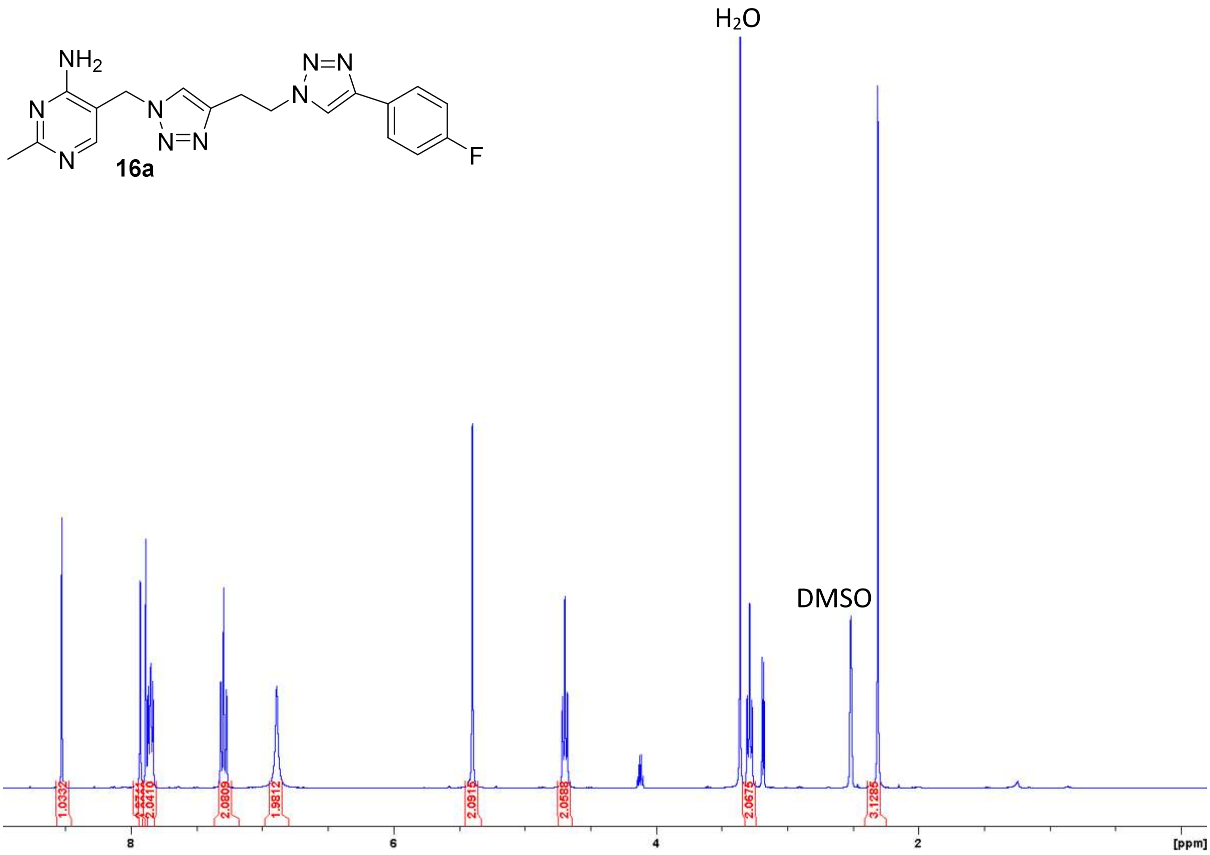

^{13}C NMR of **16a** in CD_3SOCD_3:

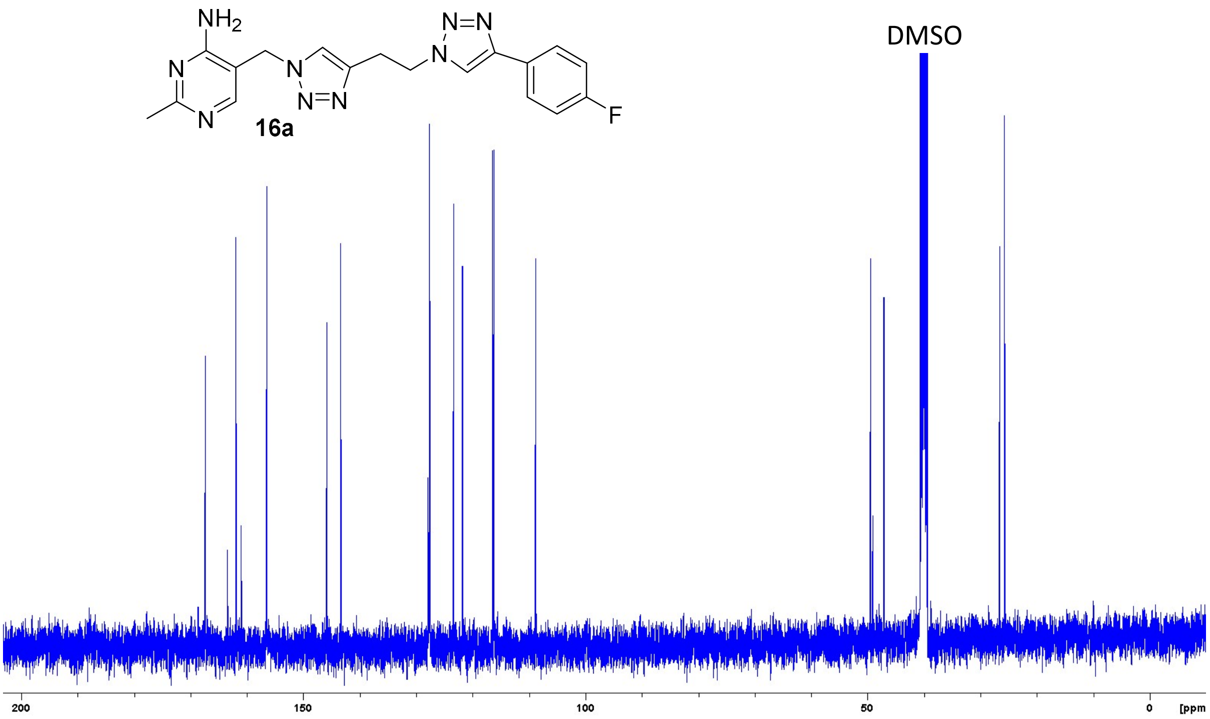

^{1}H NMR of **16b** in CD_3OD:

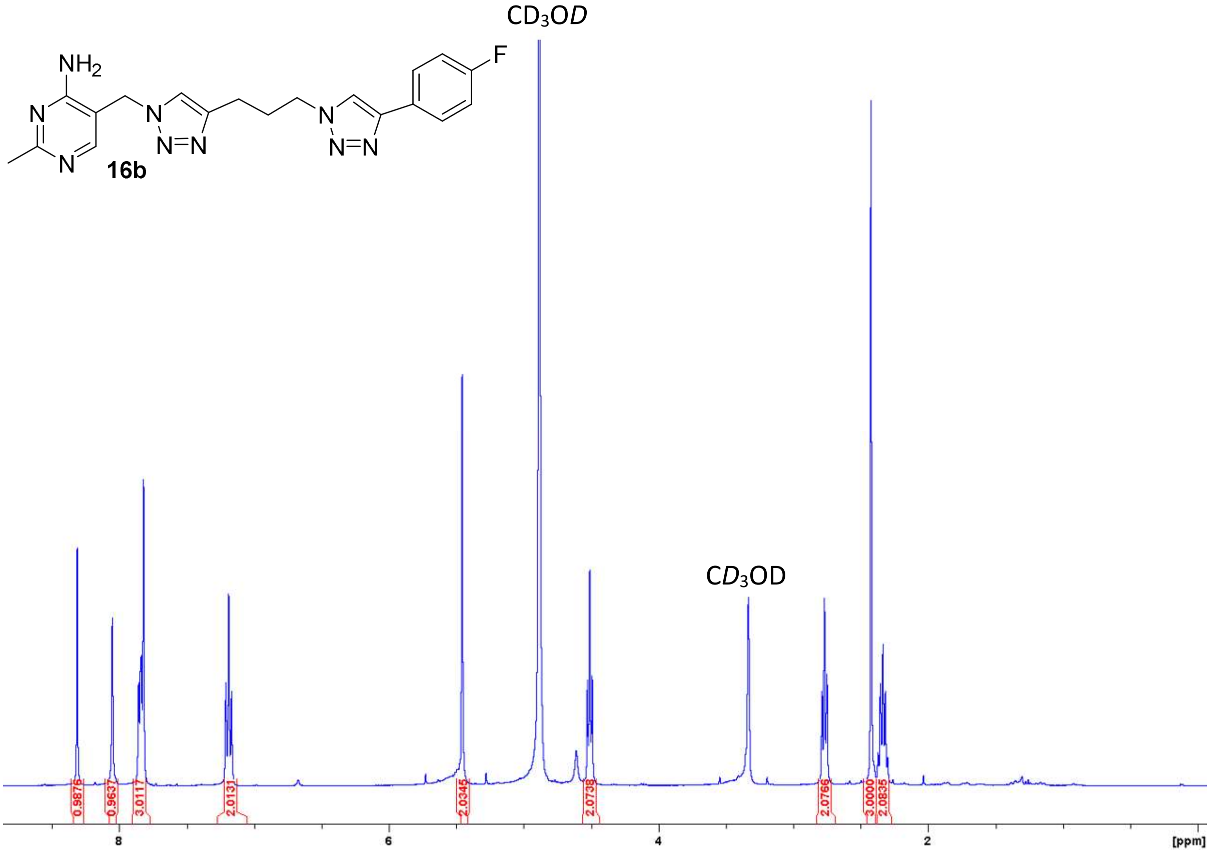

^{13}C NMR of **16b** in CD_3OD:

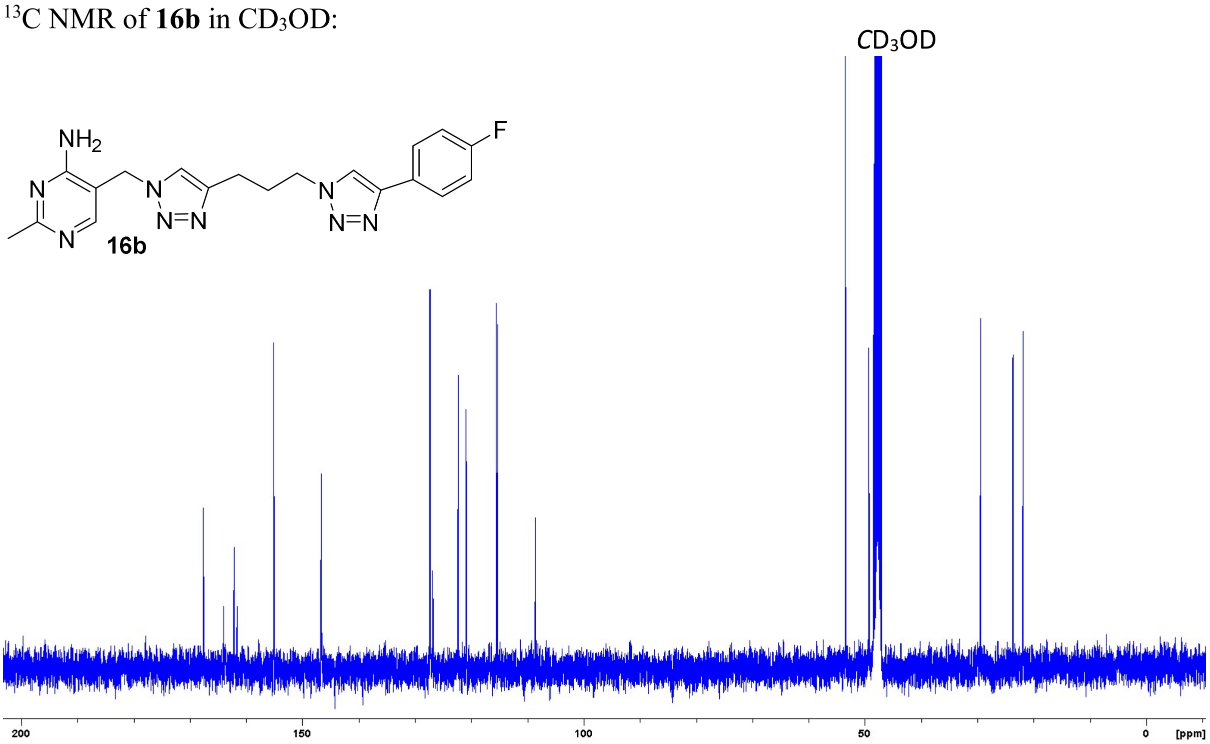

^{1}H NMR of **16c** in CD_3OD:

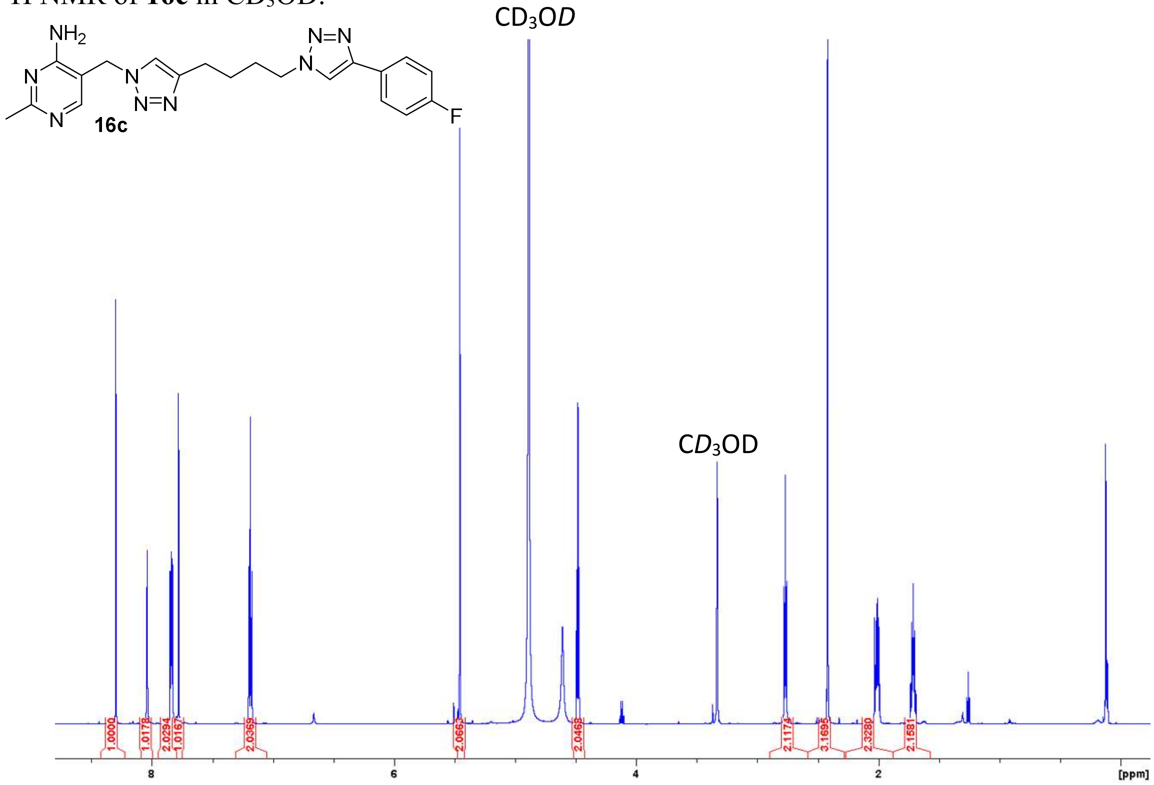

^{13}C NMR of **16c** in CD_3OD:

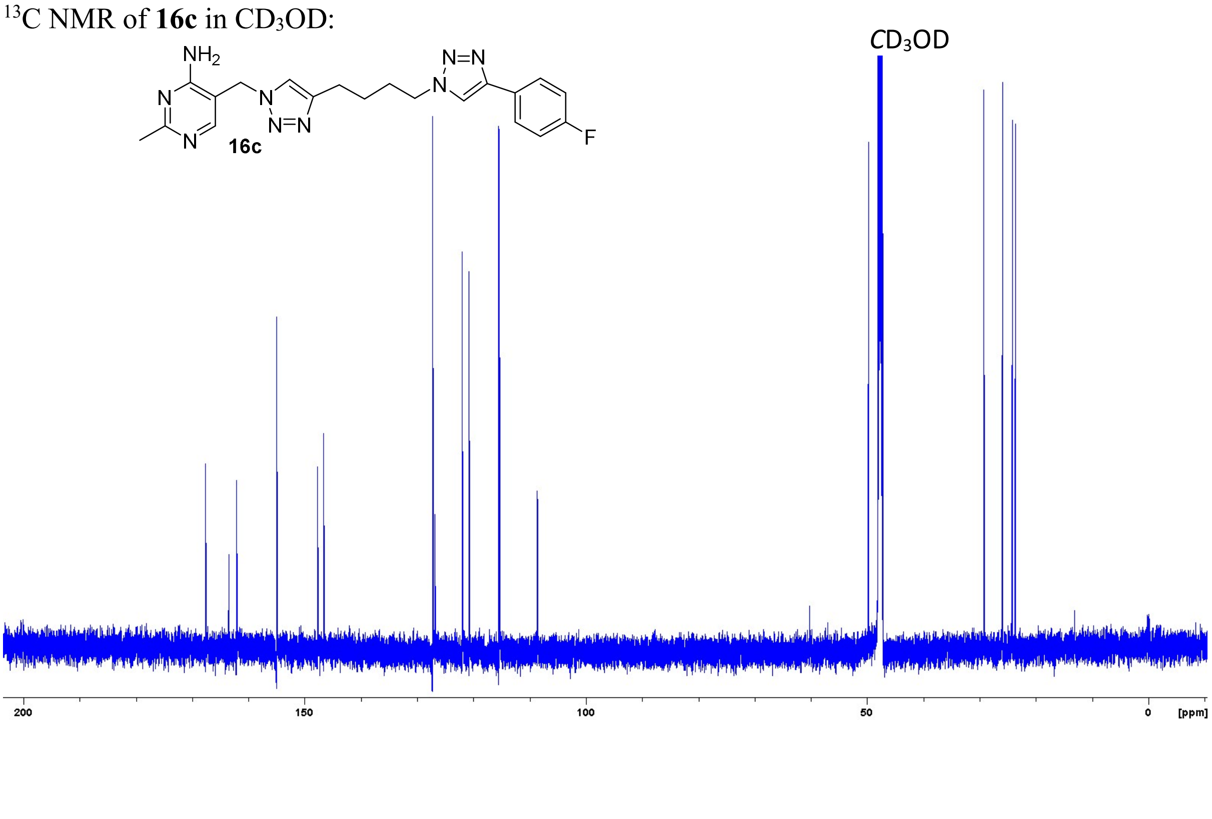

^{1}H NMR of **17a** in CD_3SOCD_3:

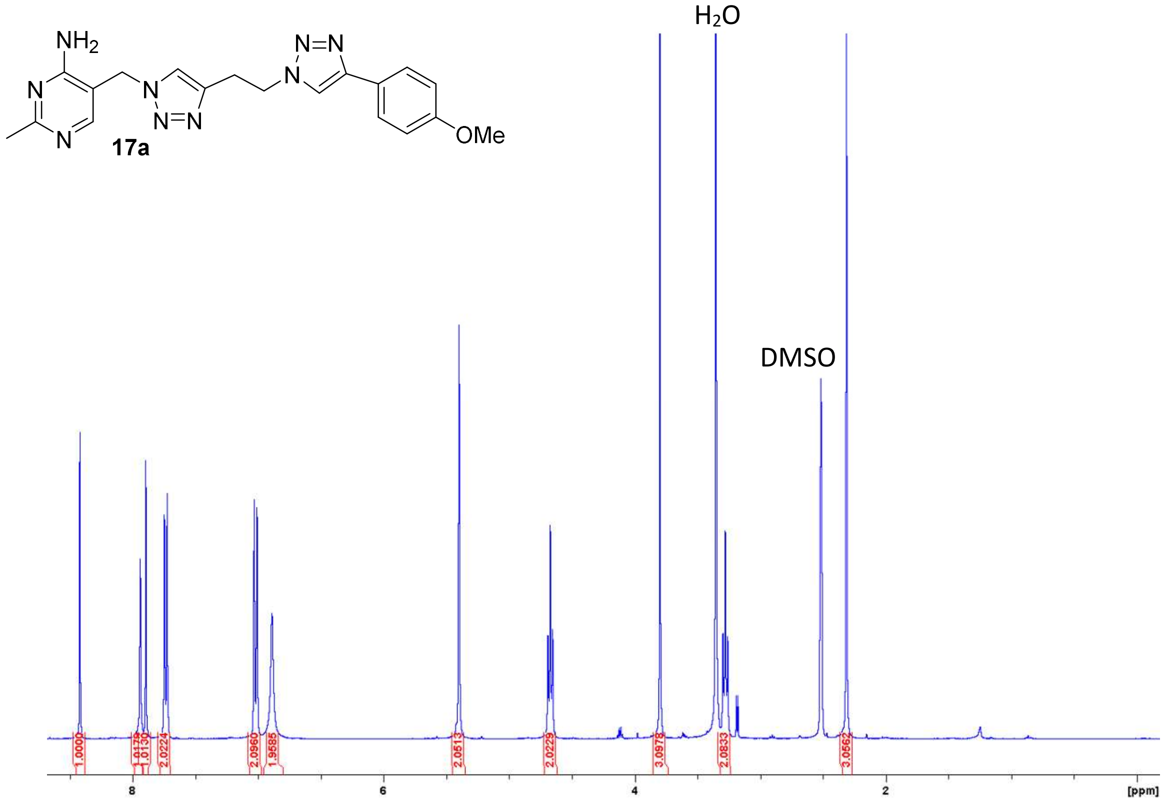

^{13}C NMR of **17a** in CD_3SOCD_3:

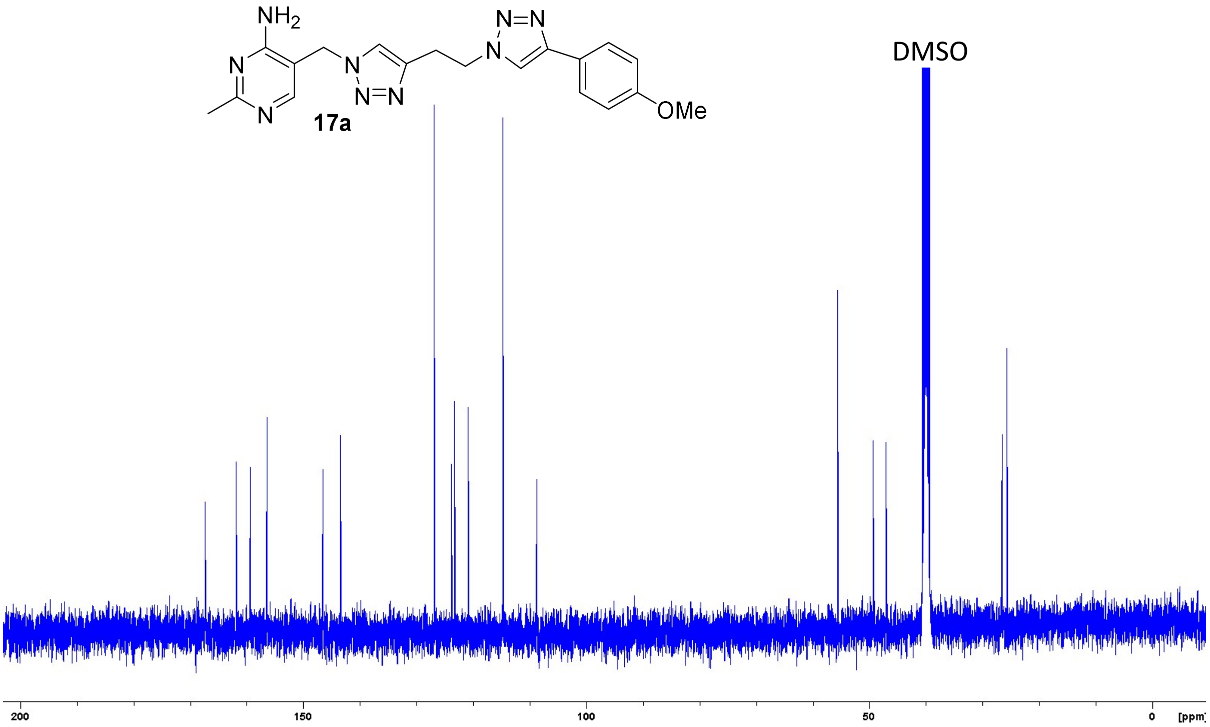

^{1}H NMR of **17b** in CD_3SOCD_3:

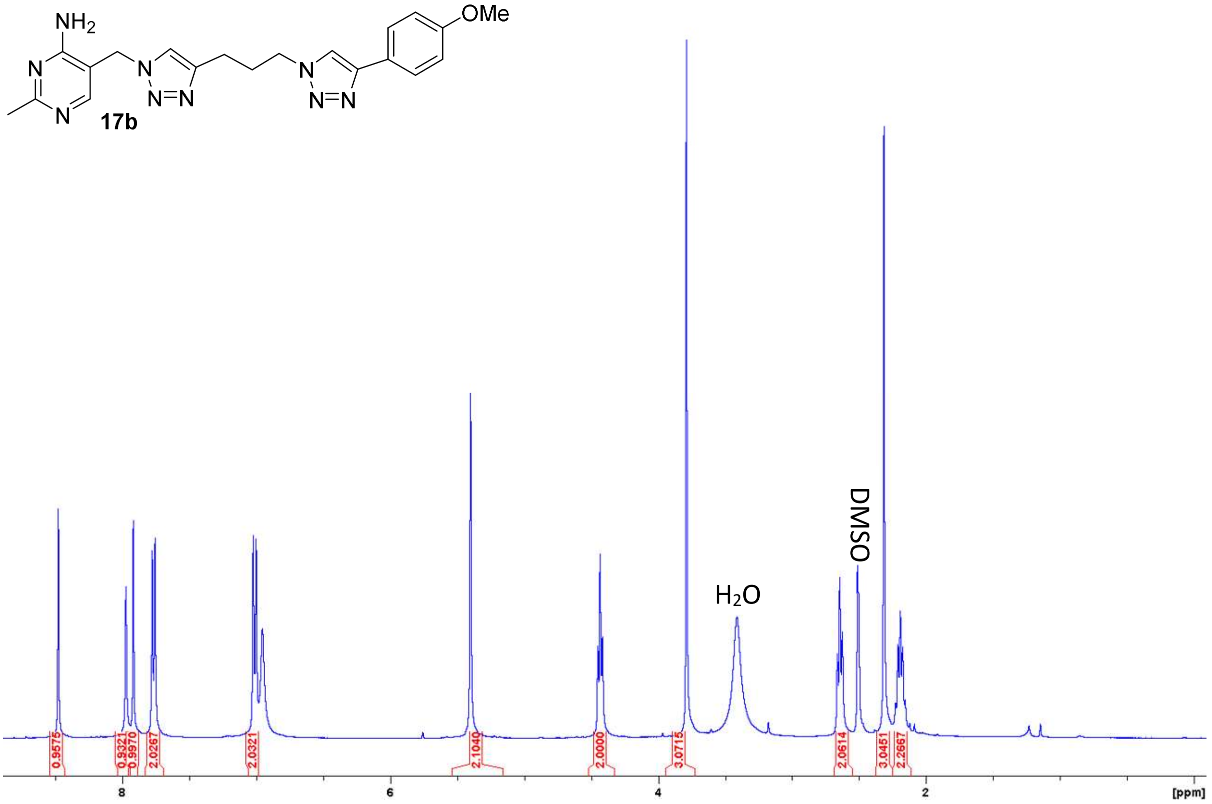

^{13}C NMR of **17b** in CD_3SOCD_3:

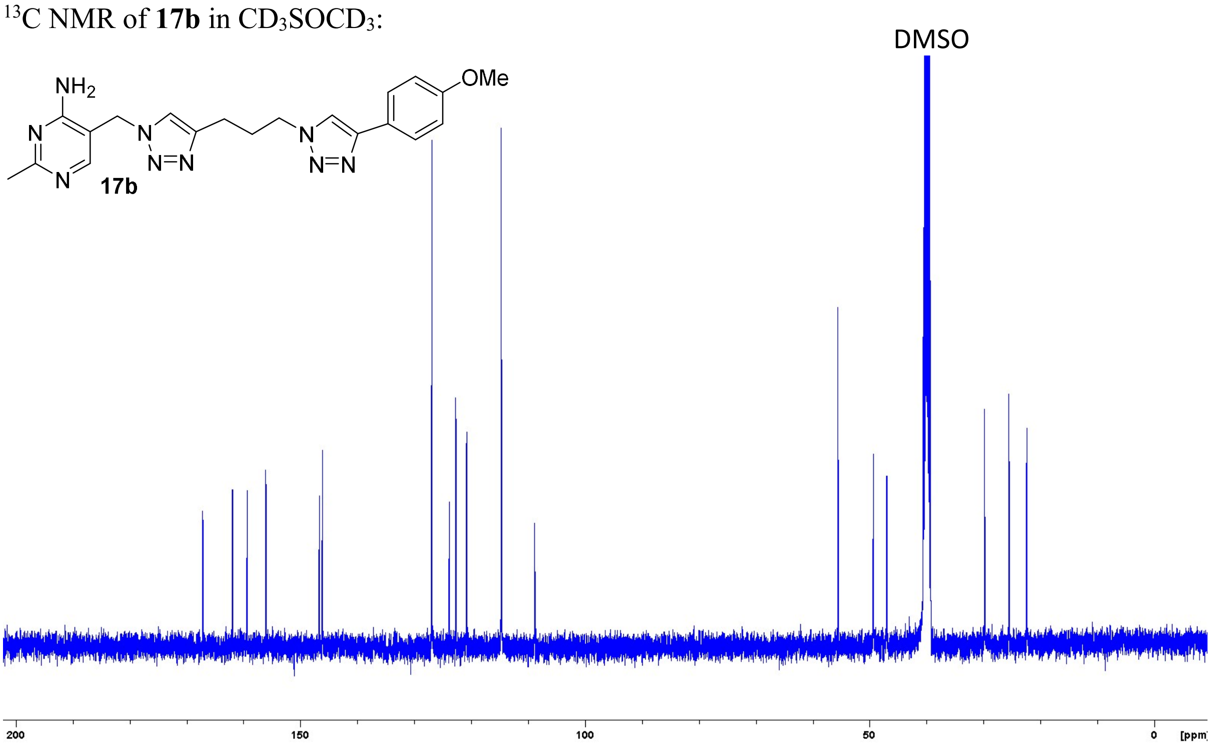

^{1}H NMR of **17c** in CD_3SOCD_3:

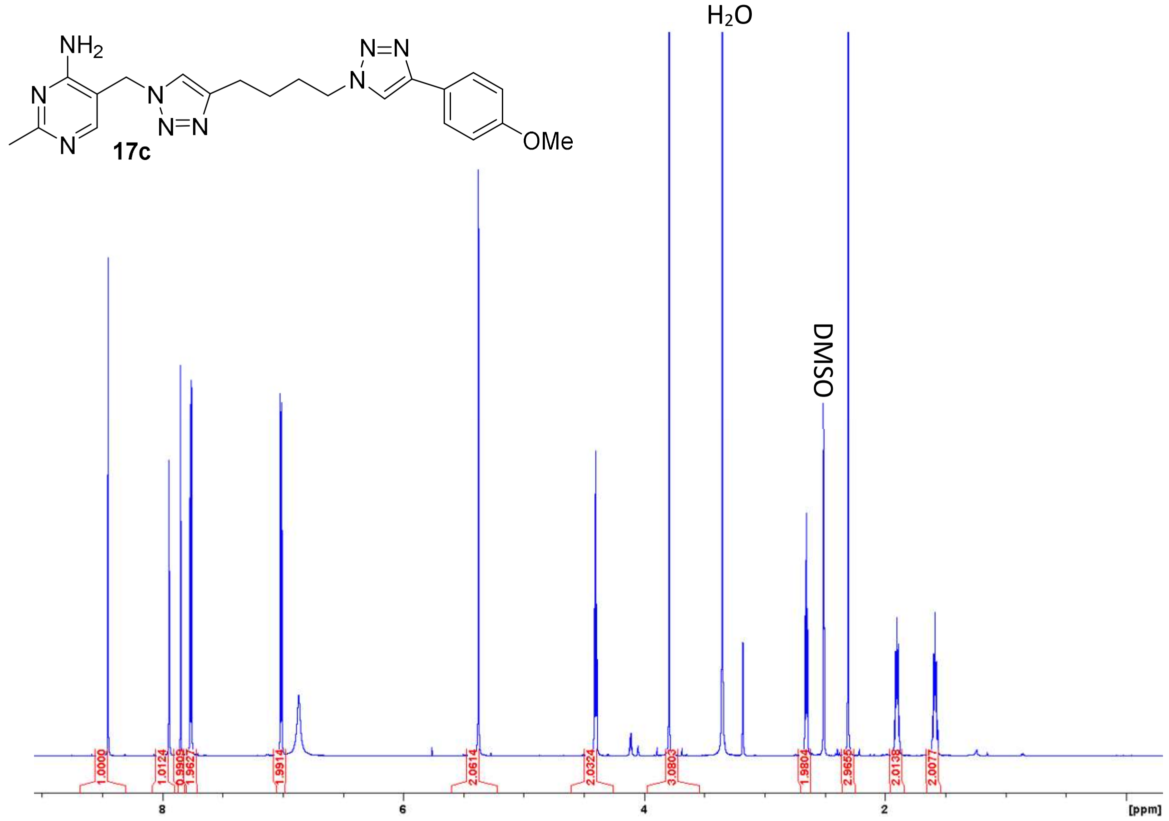

^{13}C NMR of **17c** in CD_3SOCD_3:

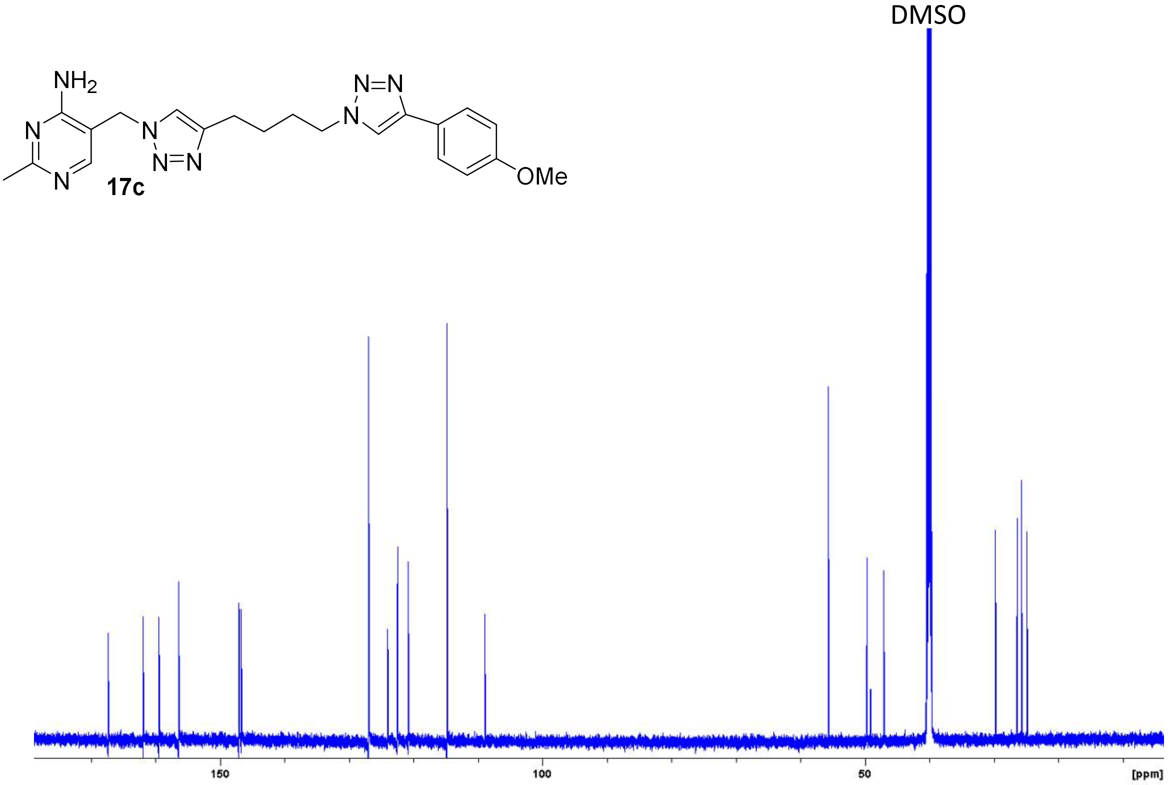

^{1}H NMR of **18a** in CD_3OD:

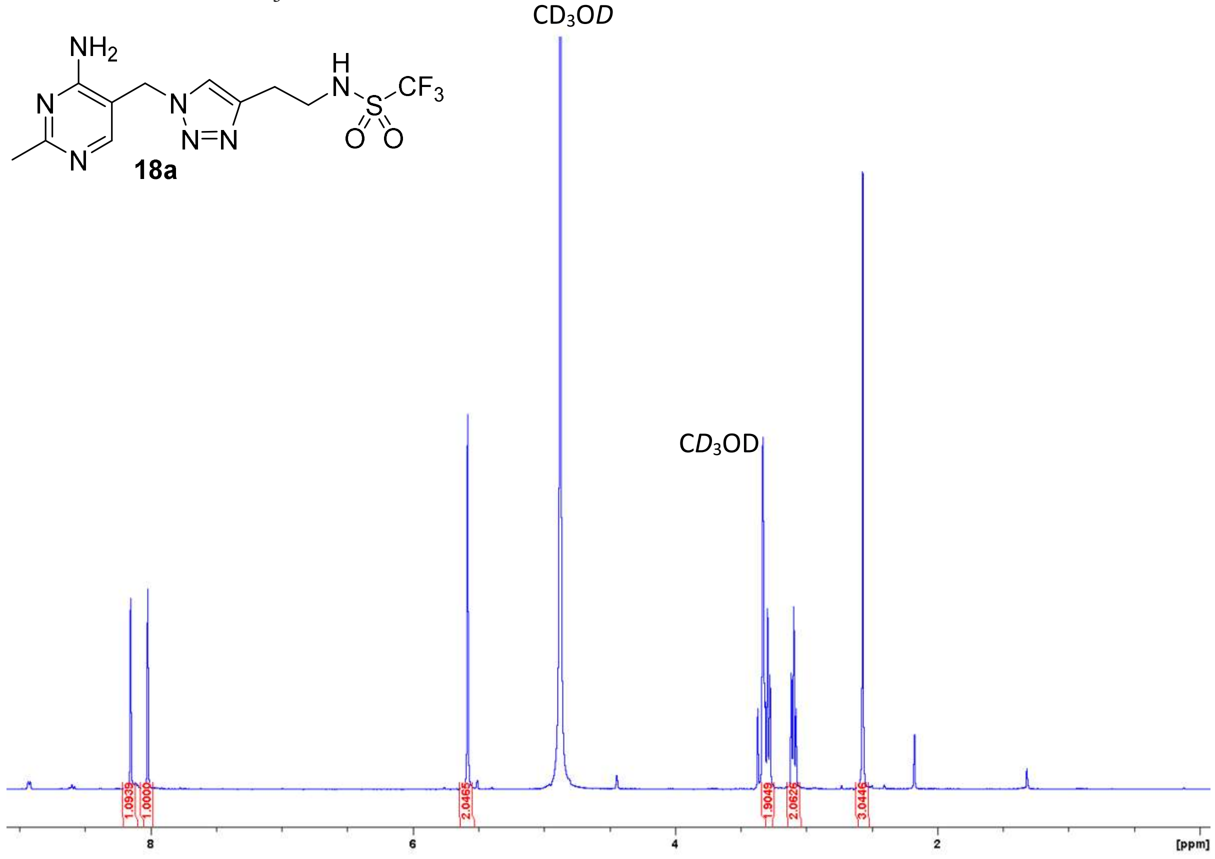

^{13}C NMR of **18a** in CD_3OD:

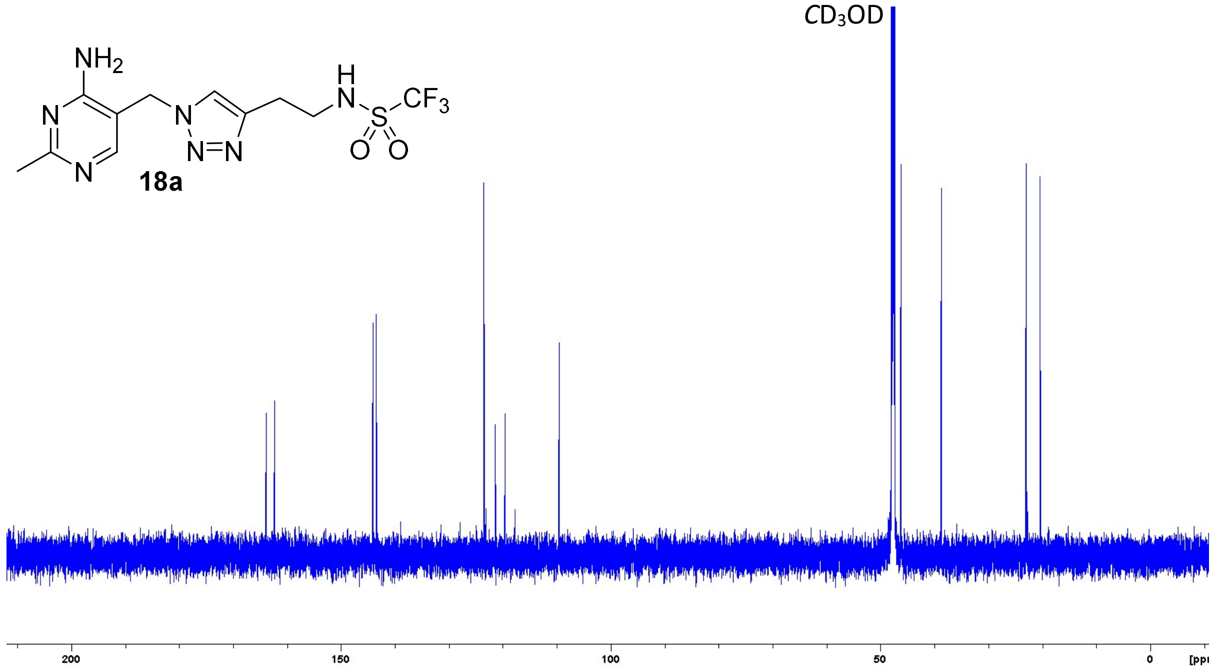

^{1}H NMR of **18b** in CD_3OD:

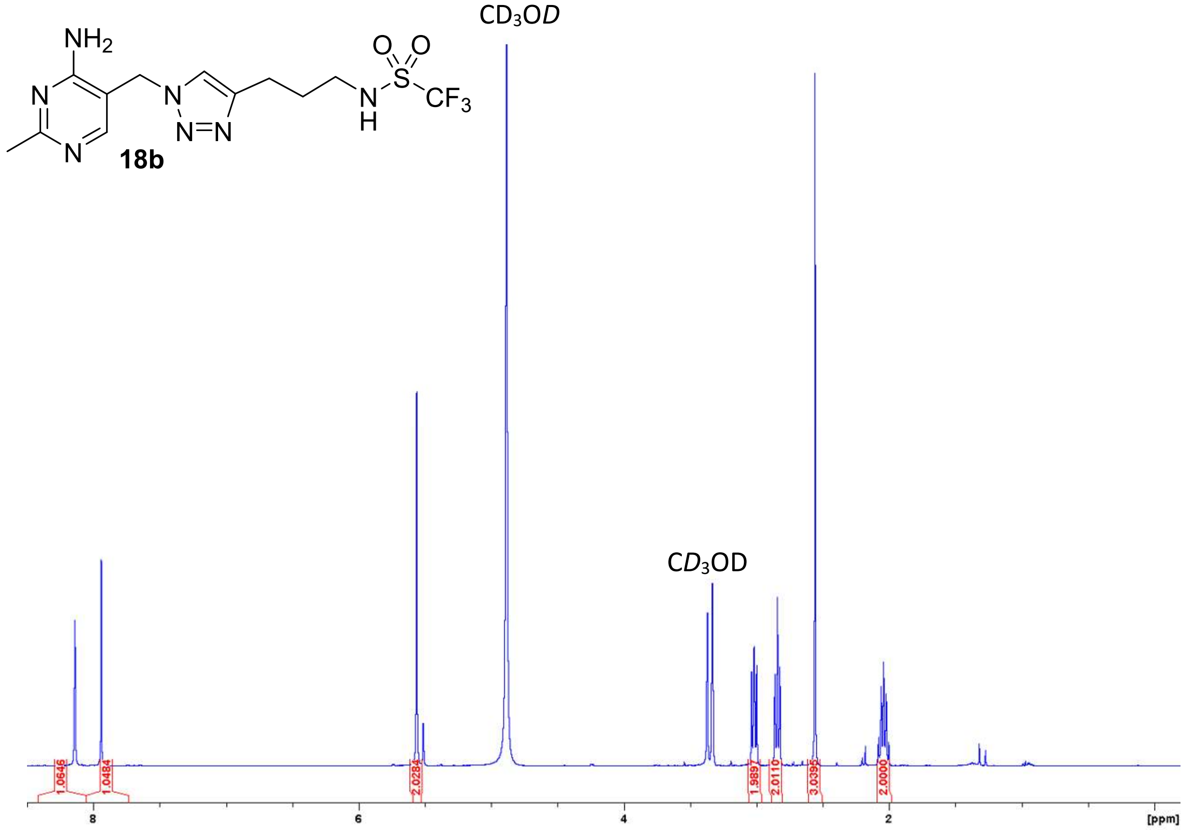

^{13}C NMR of **18b** in CD_3OD:

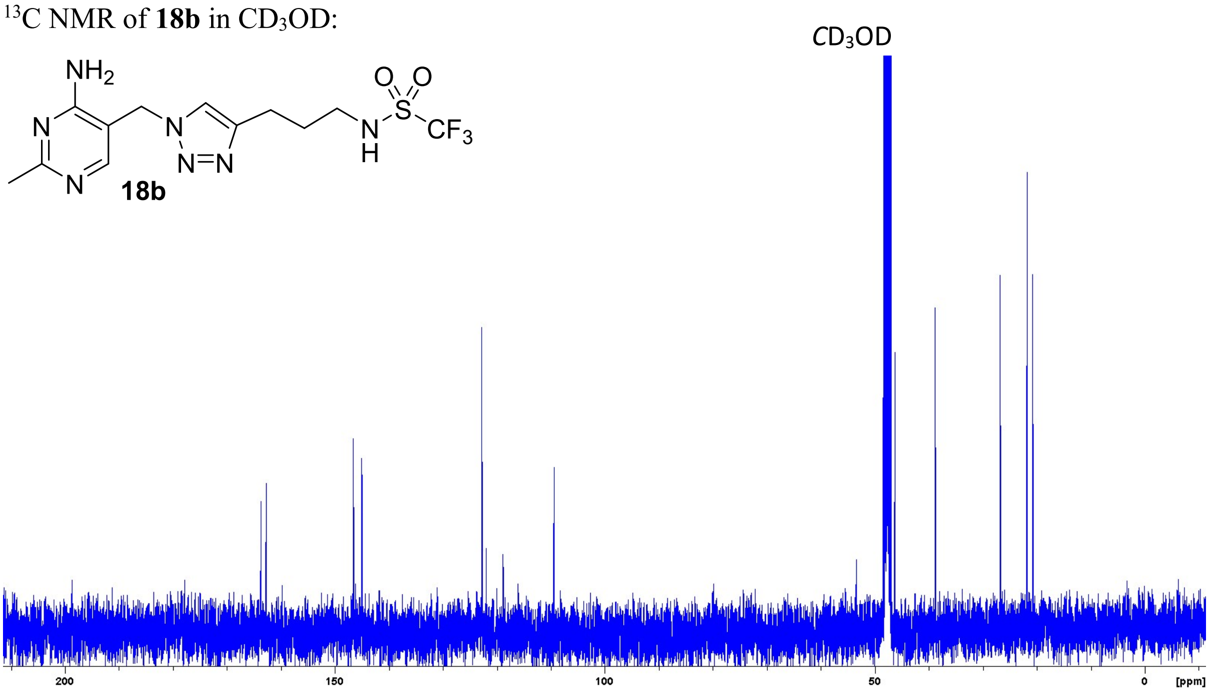

^{1}H NMR of **18c** in CD_3OD:

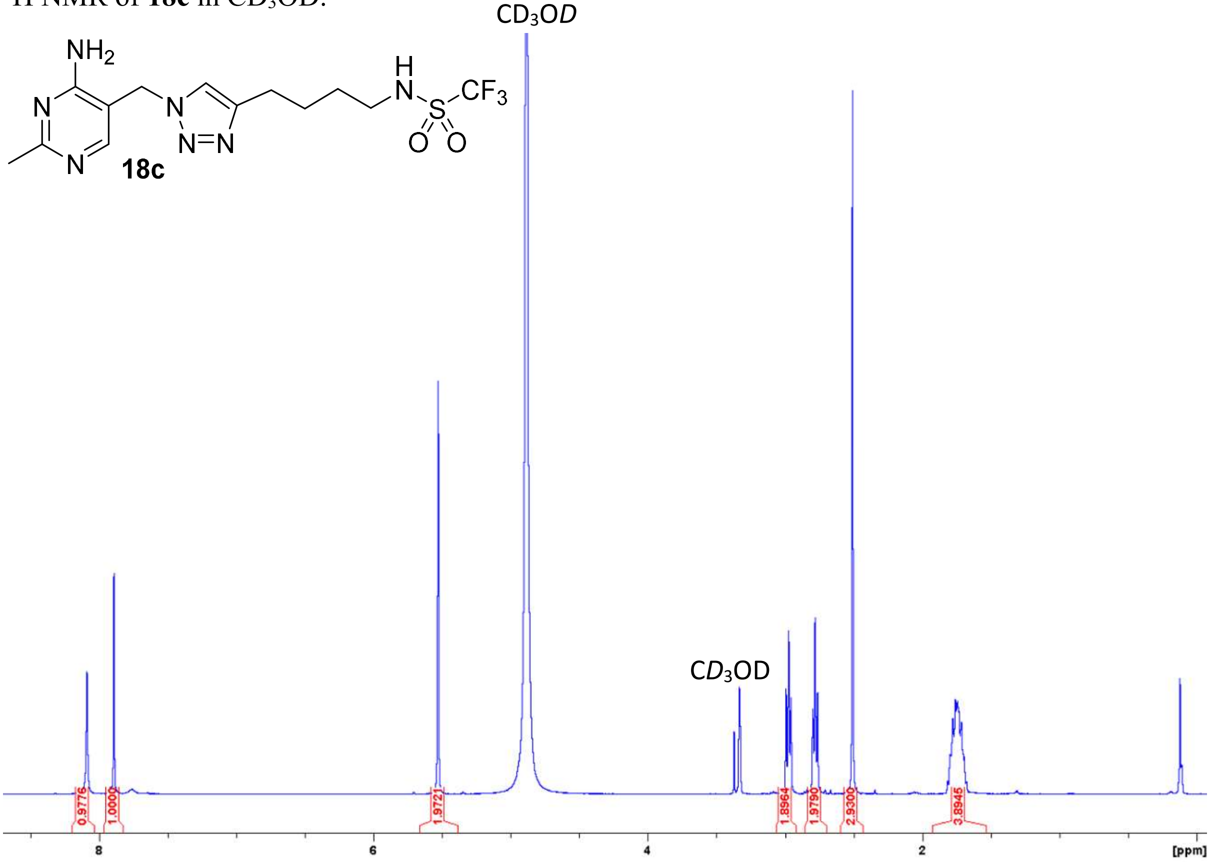

^{13}C NMR of **18c** in CD_3OD:

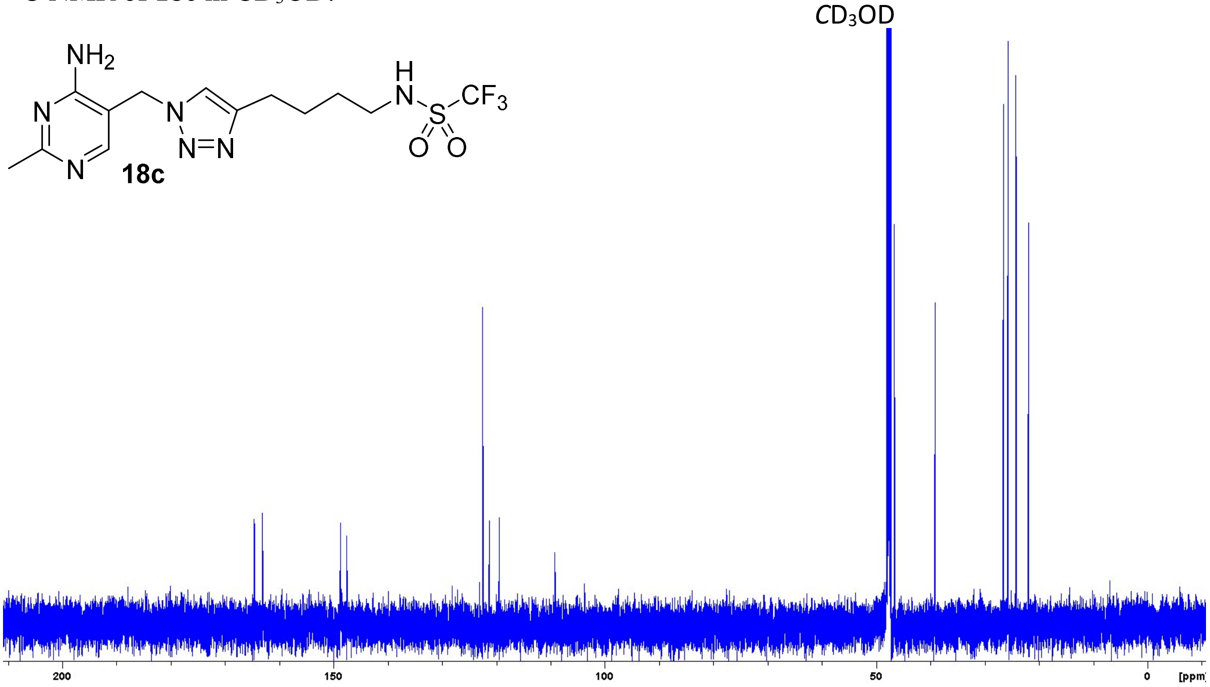

^{1}H NMR of **19a** in CD_3OD:

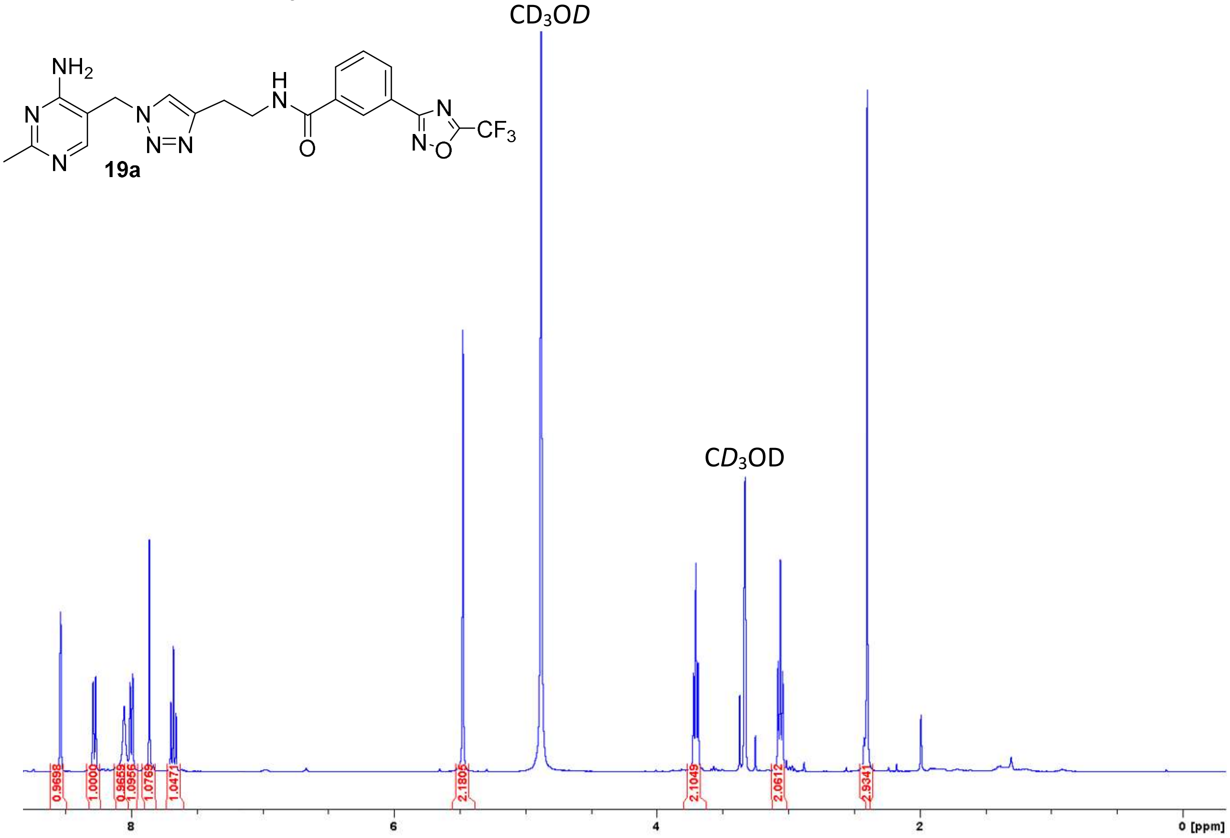

^{13}C NMR of **19a** in CD_3OD:

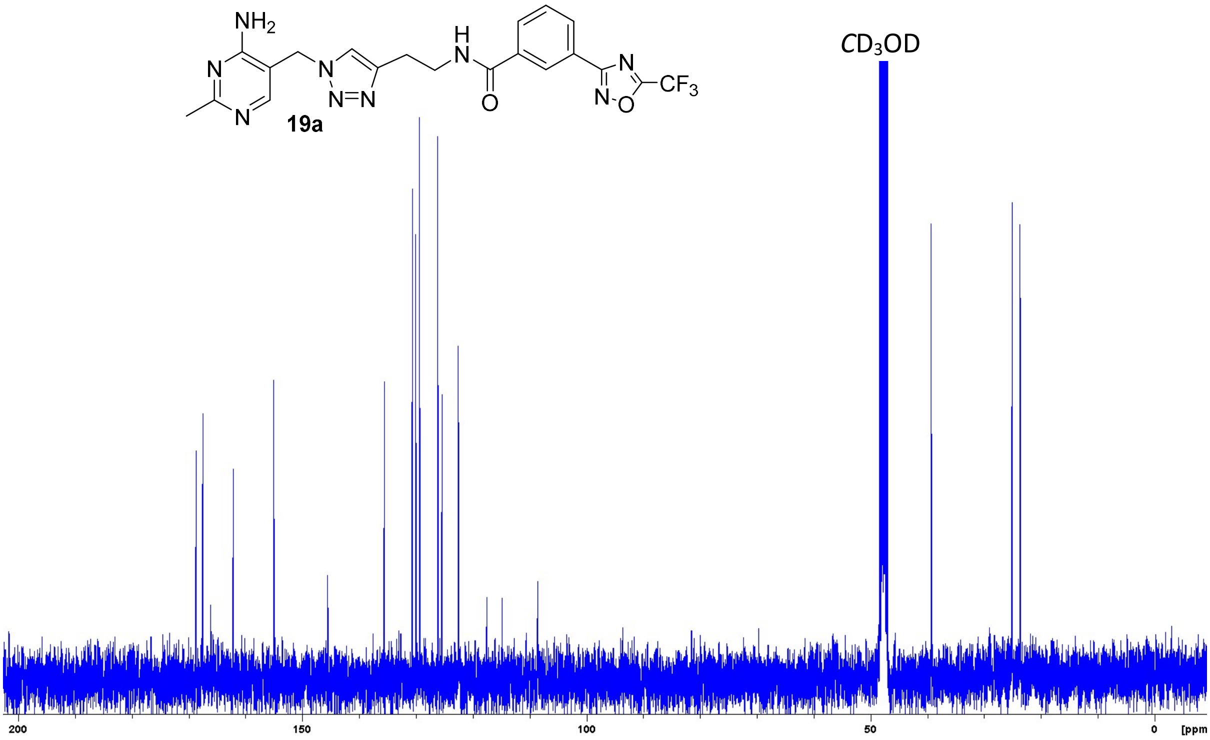

^{1}H NMR of **20a** in CD_3SOCD_3:

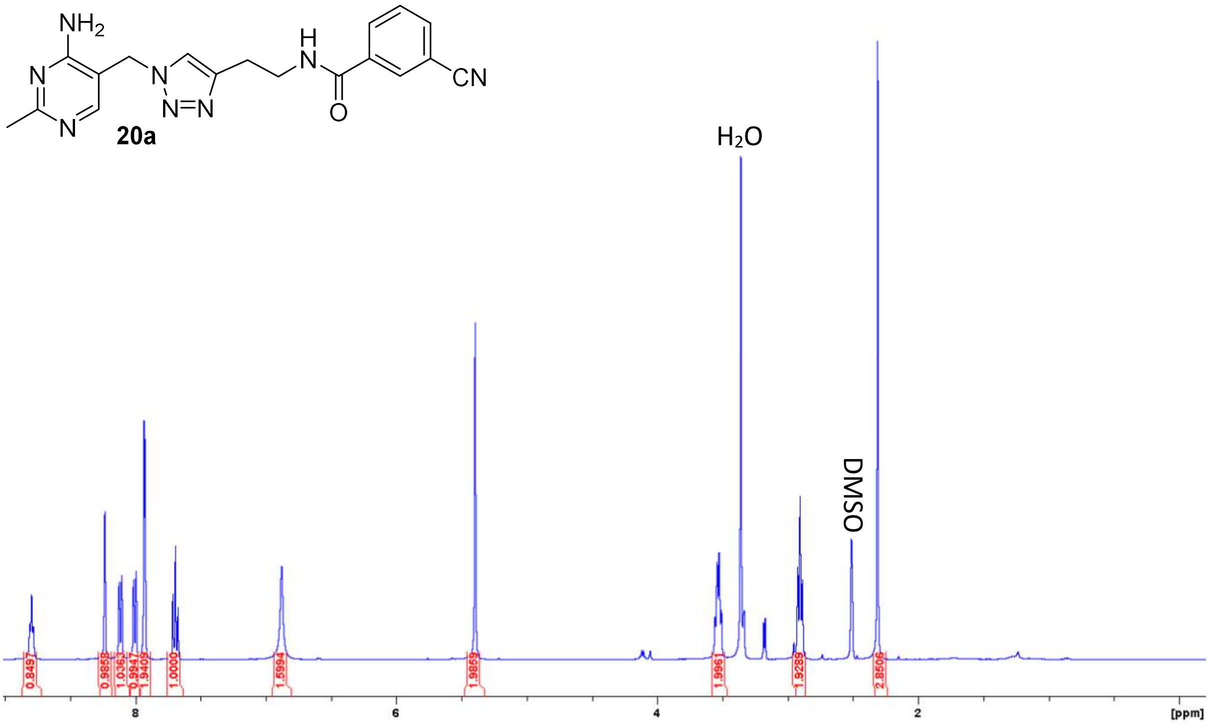

^{13}C NMR of **20a** in CD_3SOCD_3:

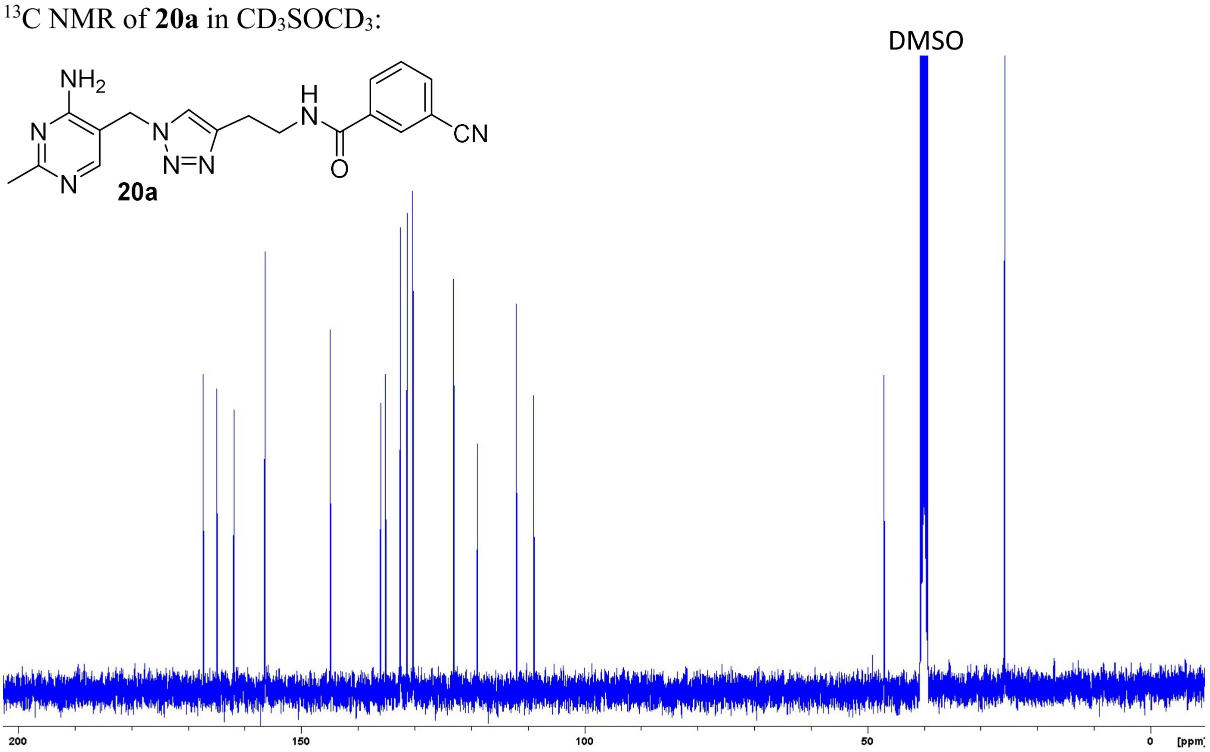

^{1}H NMR of **21a** in CD_3SOCD_3:

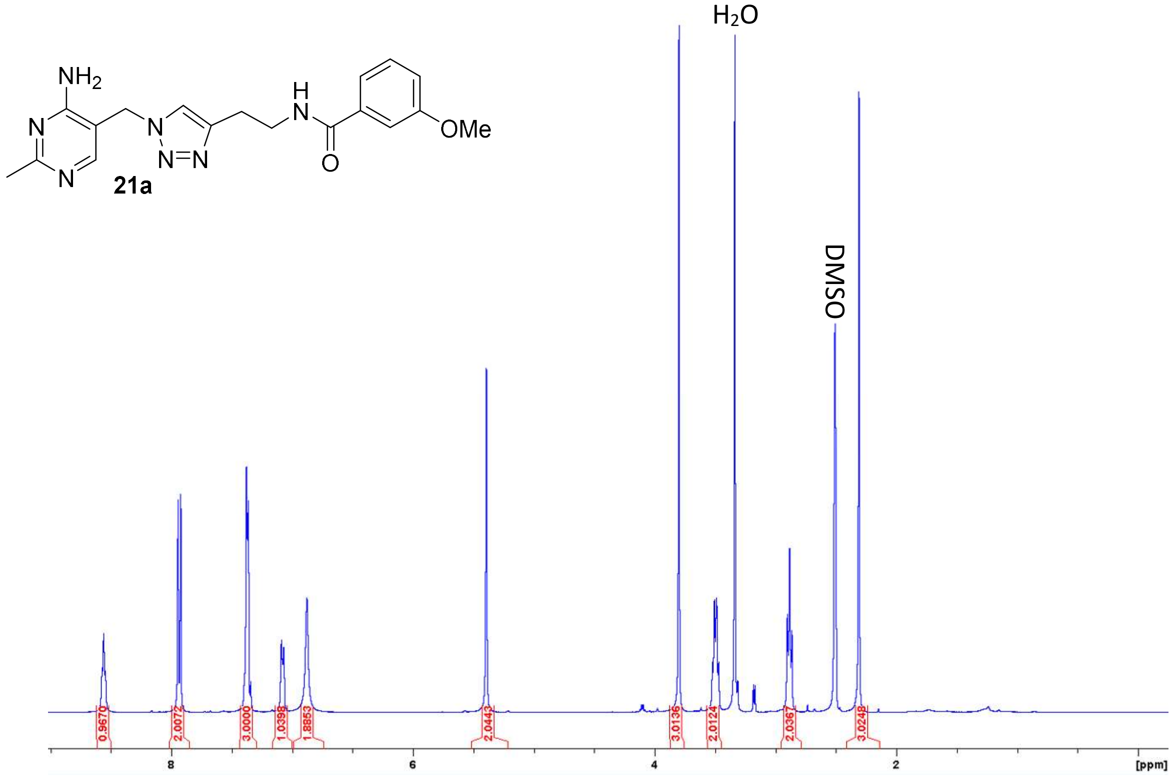

^{13}C NMR of **21a** in CD_3SOCD_3:

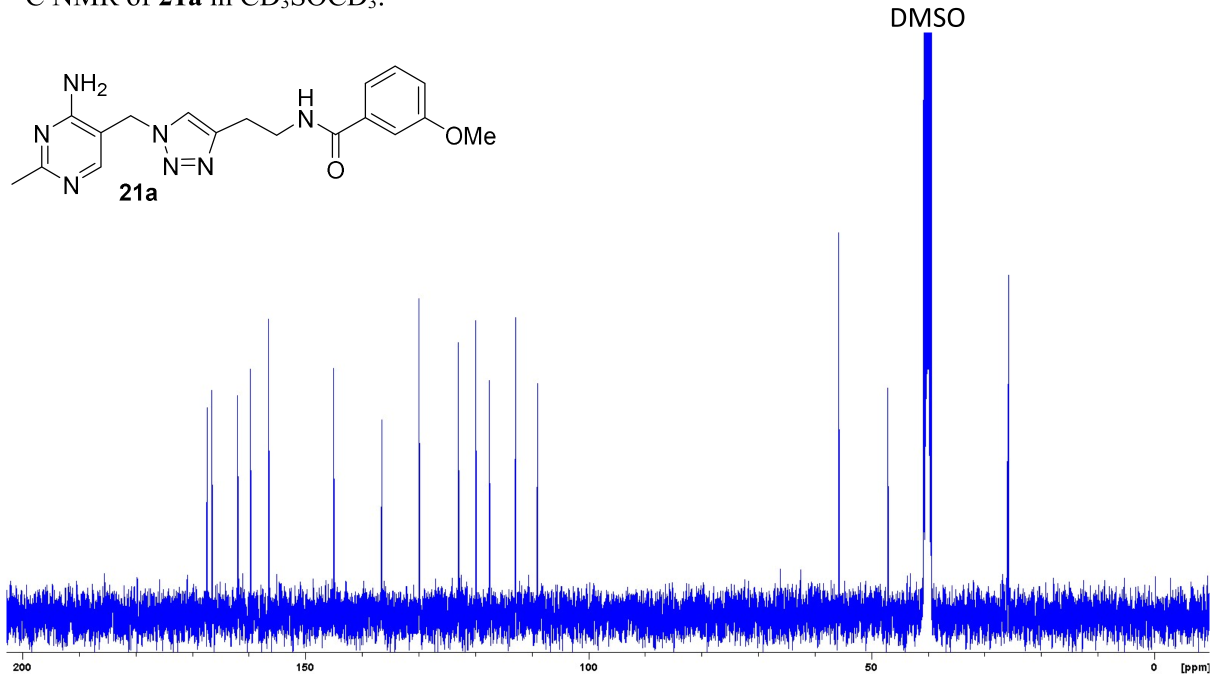

^{1}H NMR of **21b** in CD_3OD:

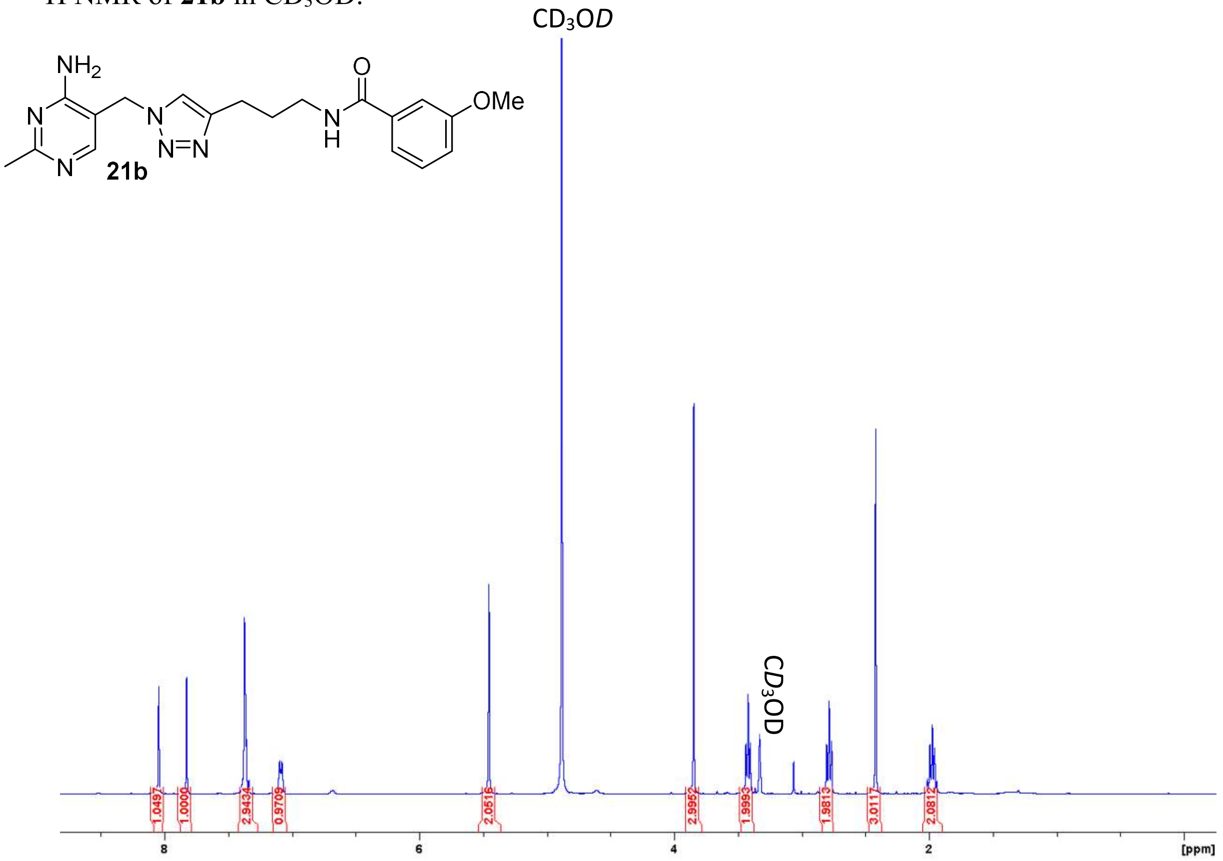

^{13}C NMR of **21b** in CD_3OD:

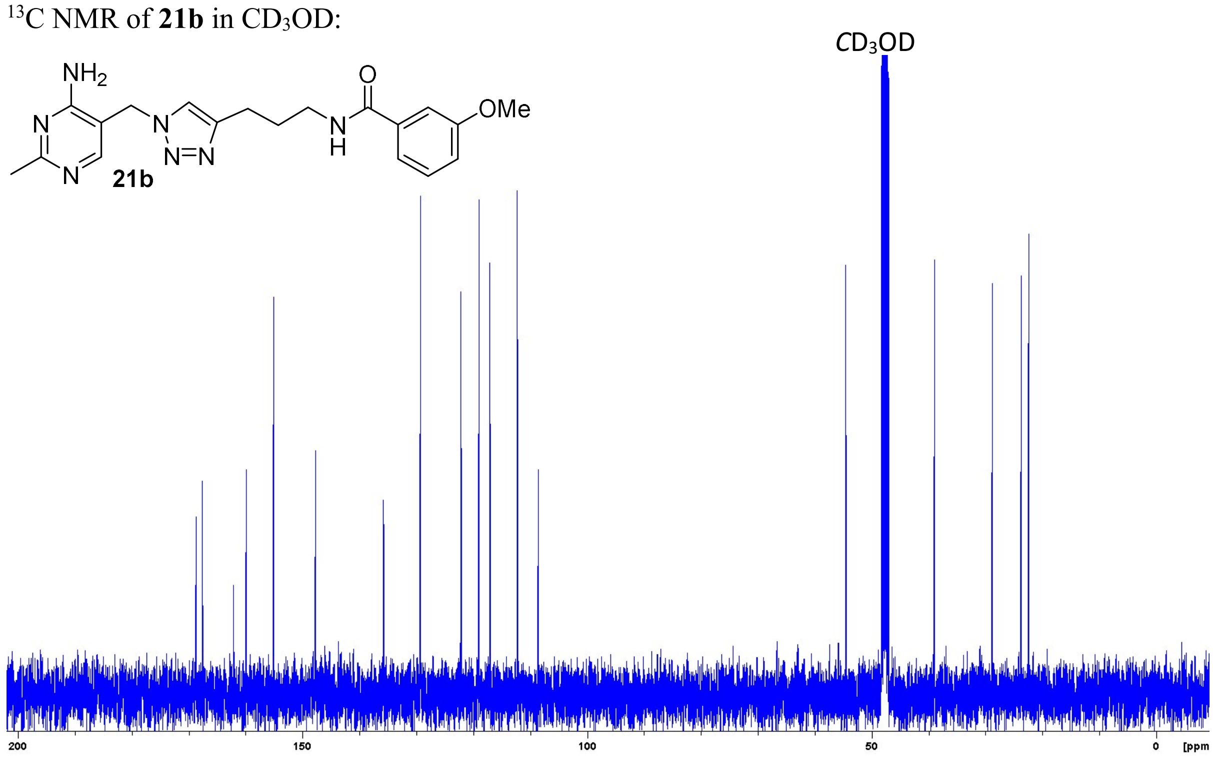

^{1}H NMR of **21c** in CD_3OD:

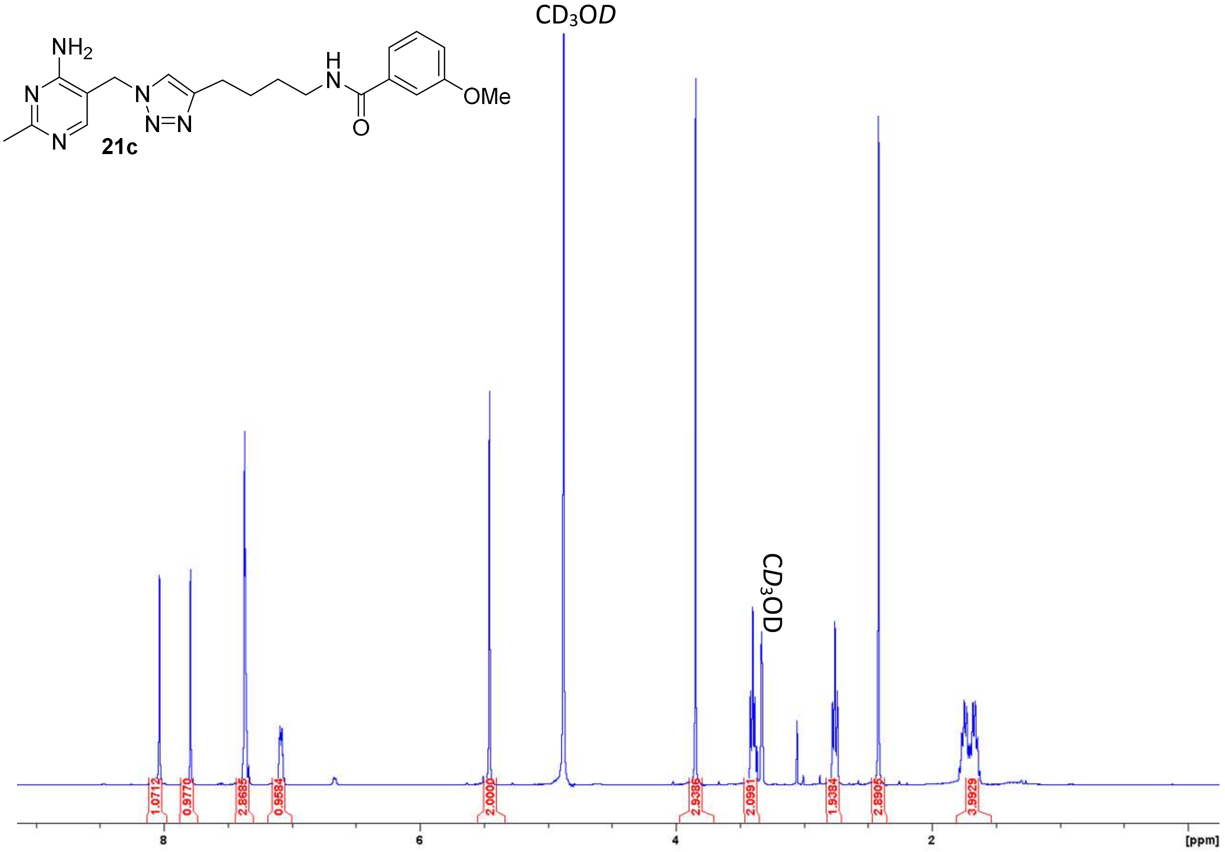

^{13}C NMR of **21c** in CD_3OD:

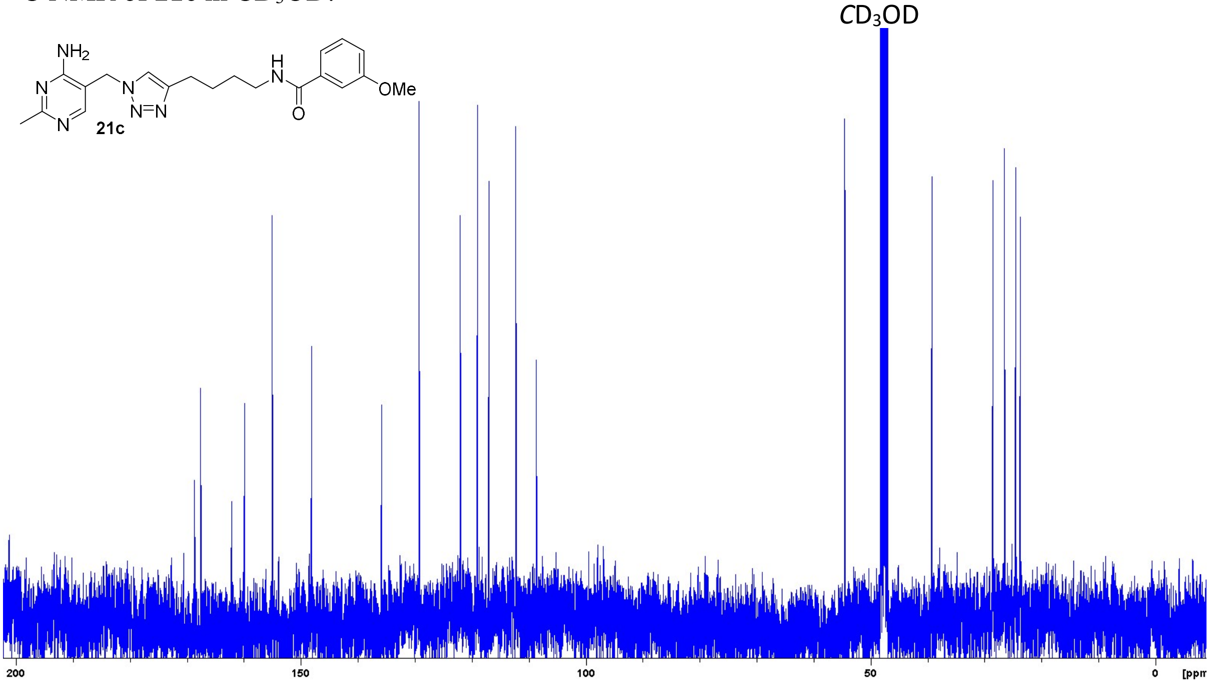

^{1}H NMR of **22a** in CD_3SOCD_3:

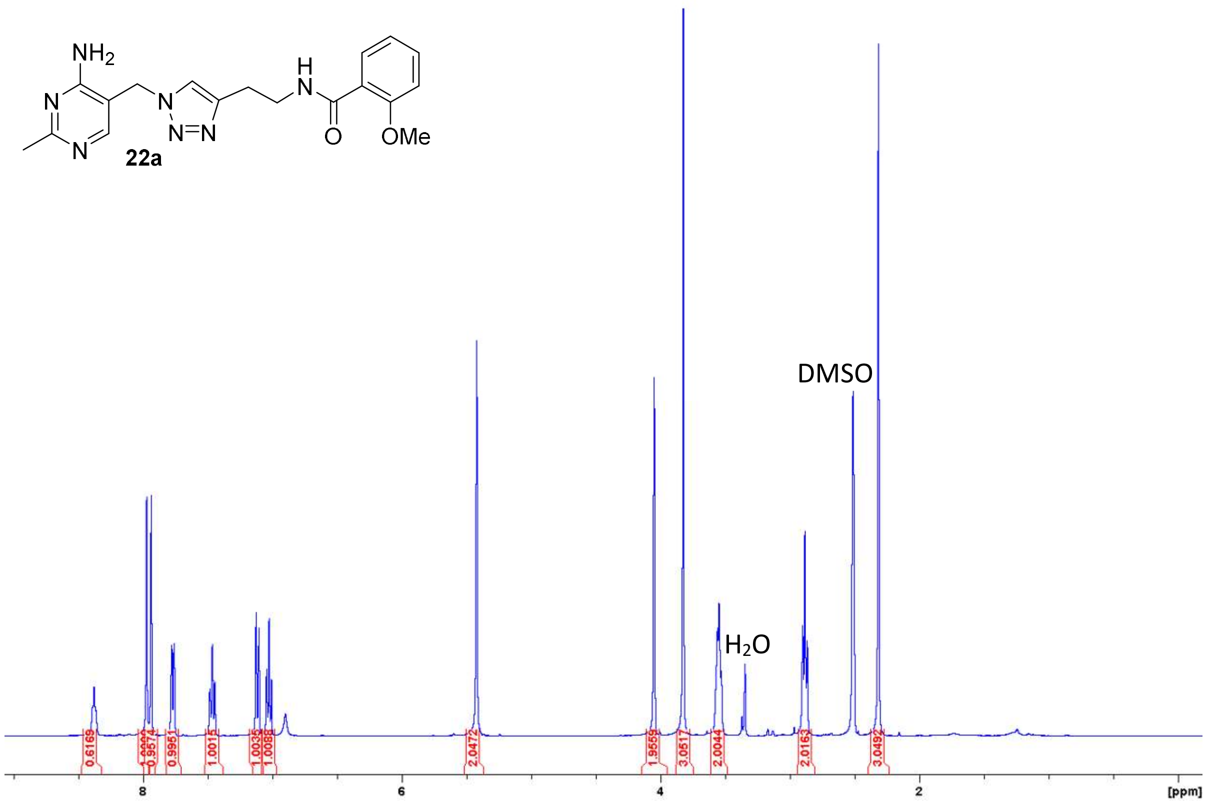

^{13}C NMR of **22a** in CD_3SOCD_3:

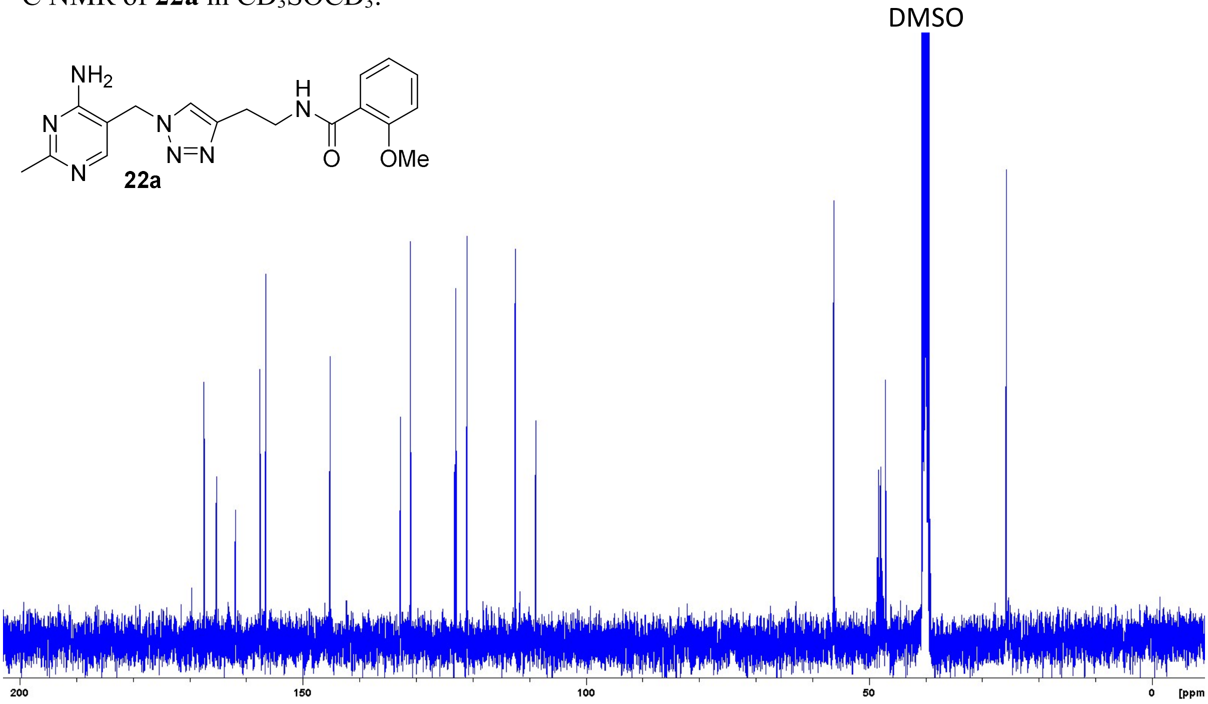

^{1}H NMR of **22b** in CD_3OD:

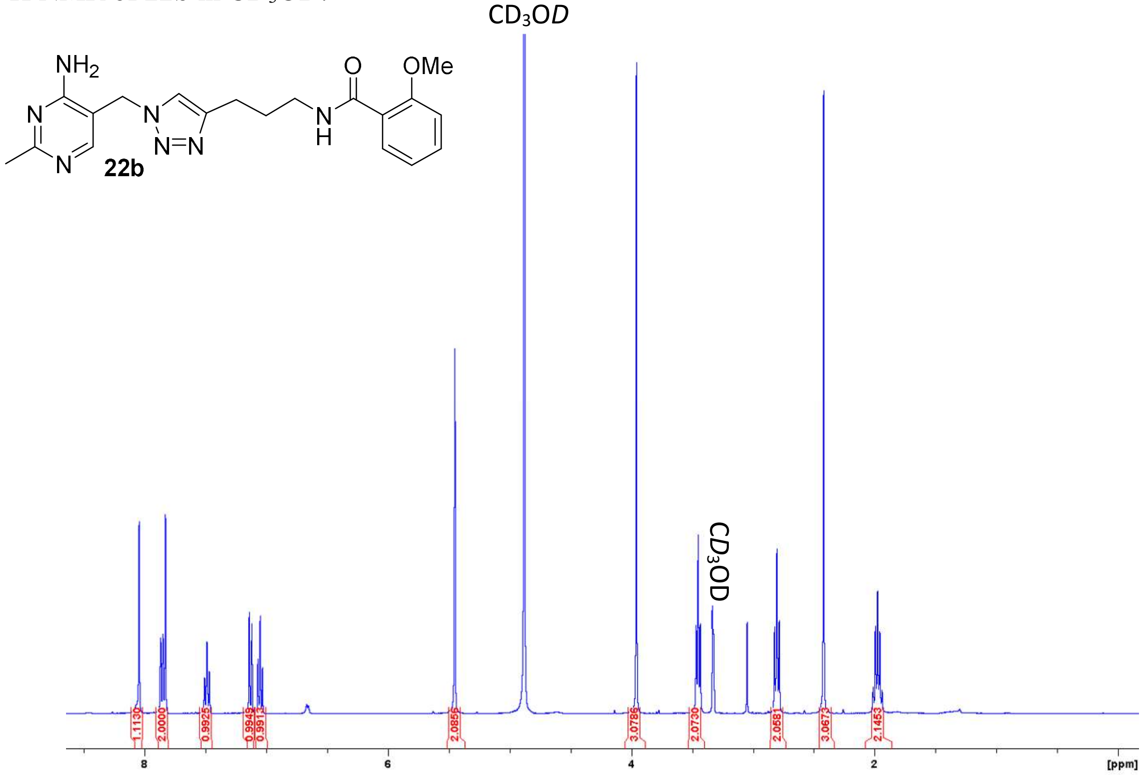

^{13}C NMR of **22b** in CD_3OD:

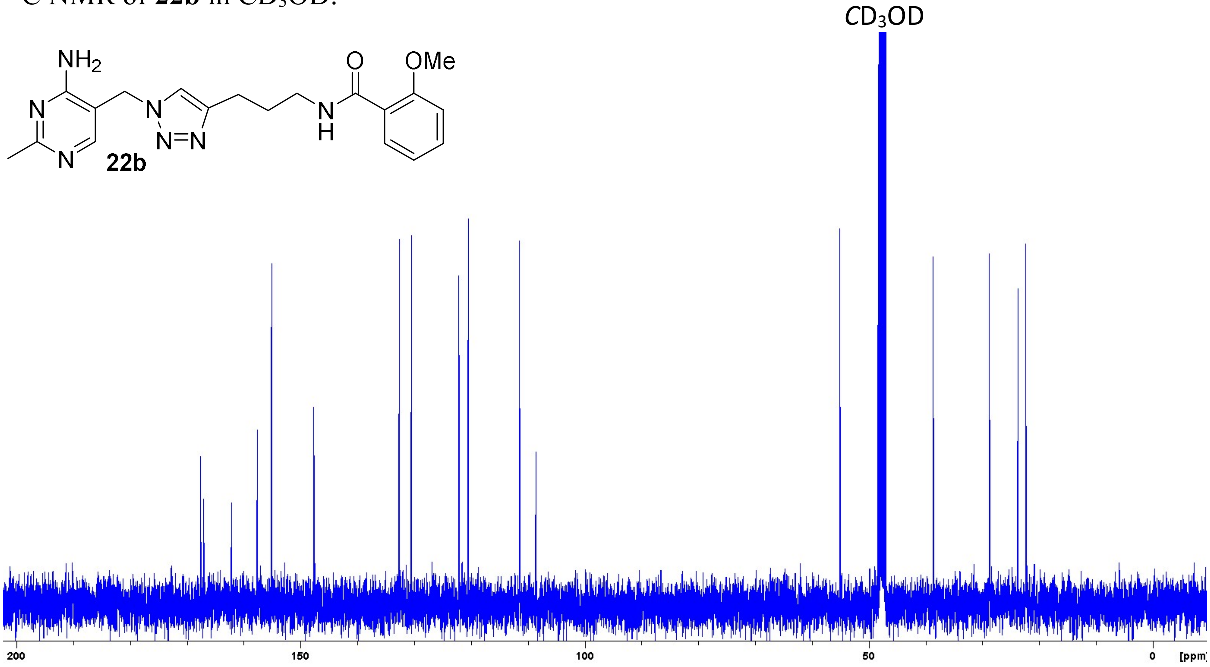

^{1}H NMR of **22c** in CD_3OD:

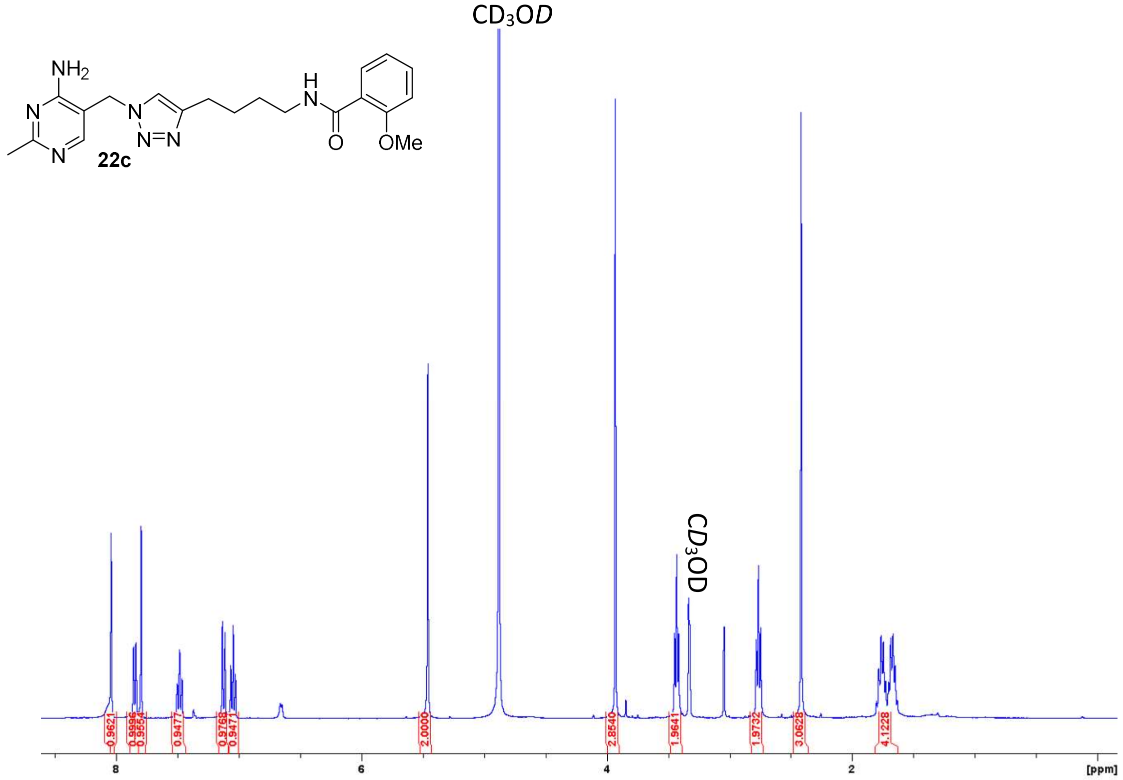

^{13}C NMR of **22c** in CD_3OD:

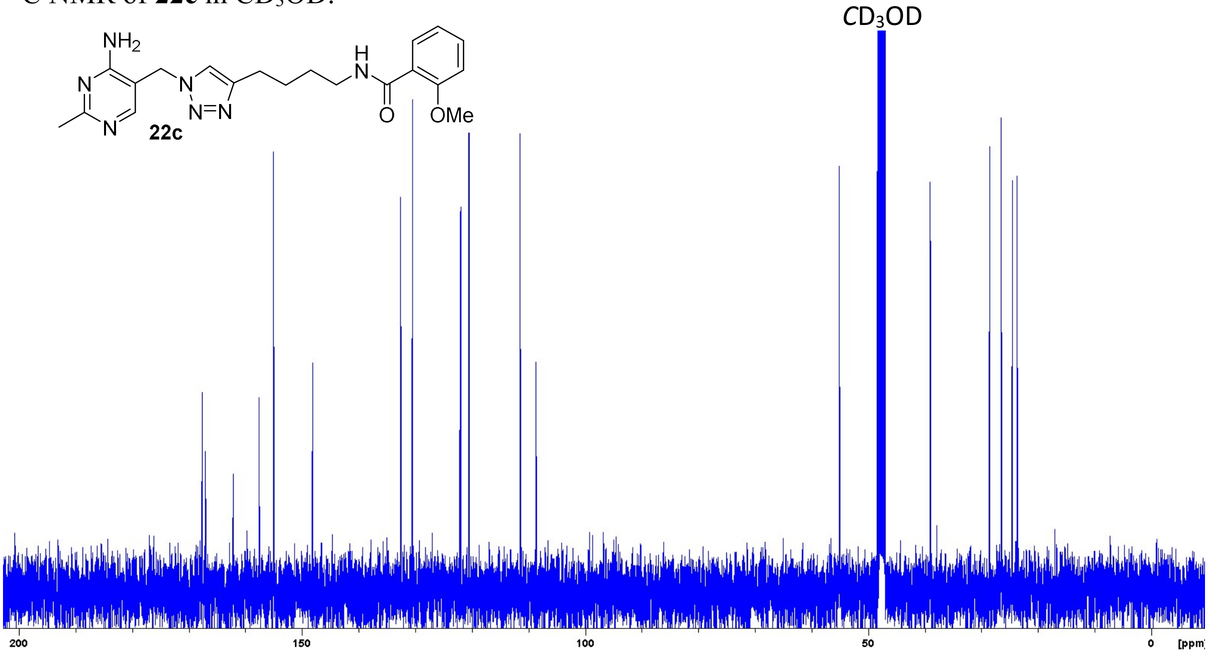

^{1}H NMR of *O*-Bn-**24a** in CD_3OD:

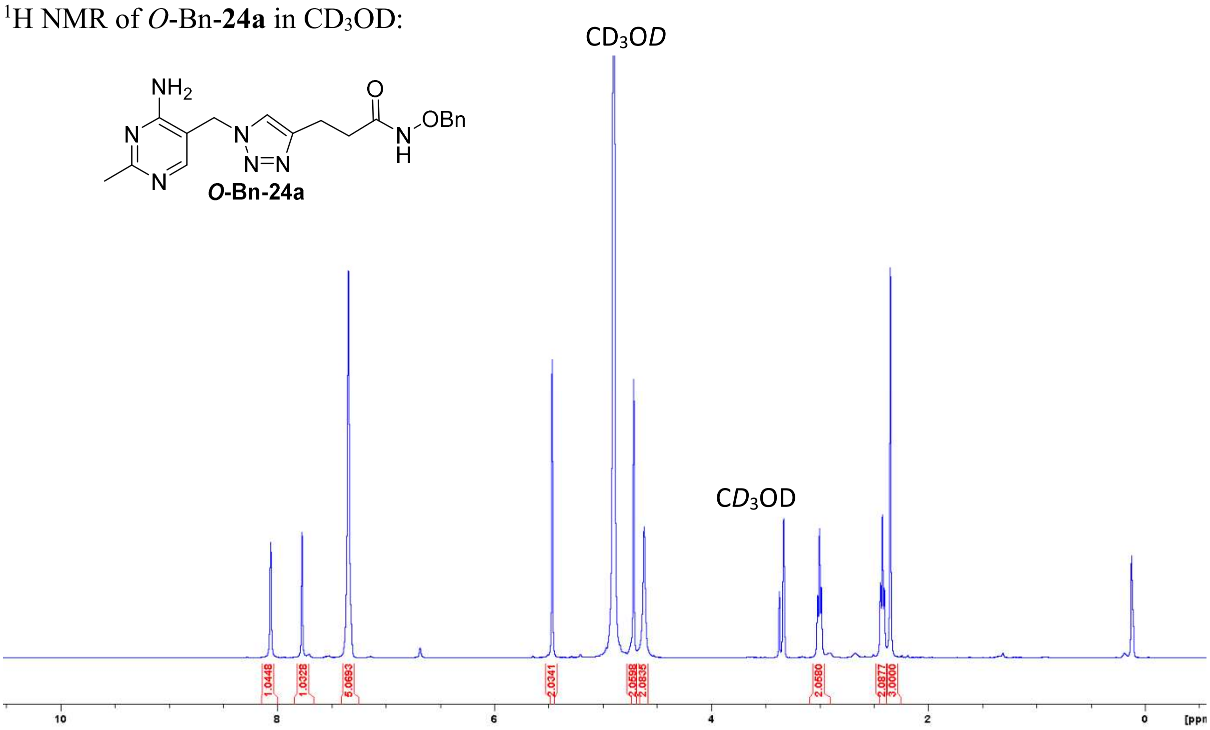

^{13}C NMR of *O*-Bn-**24a** in CD_3OD:

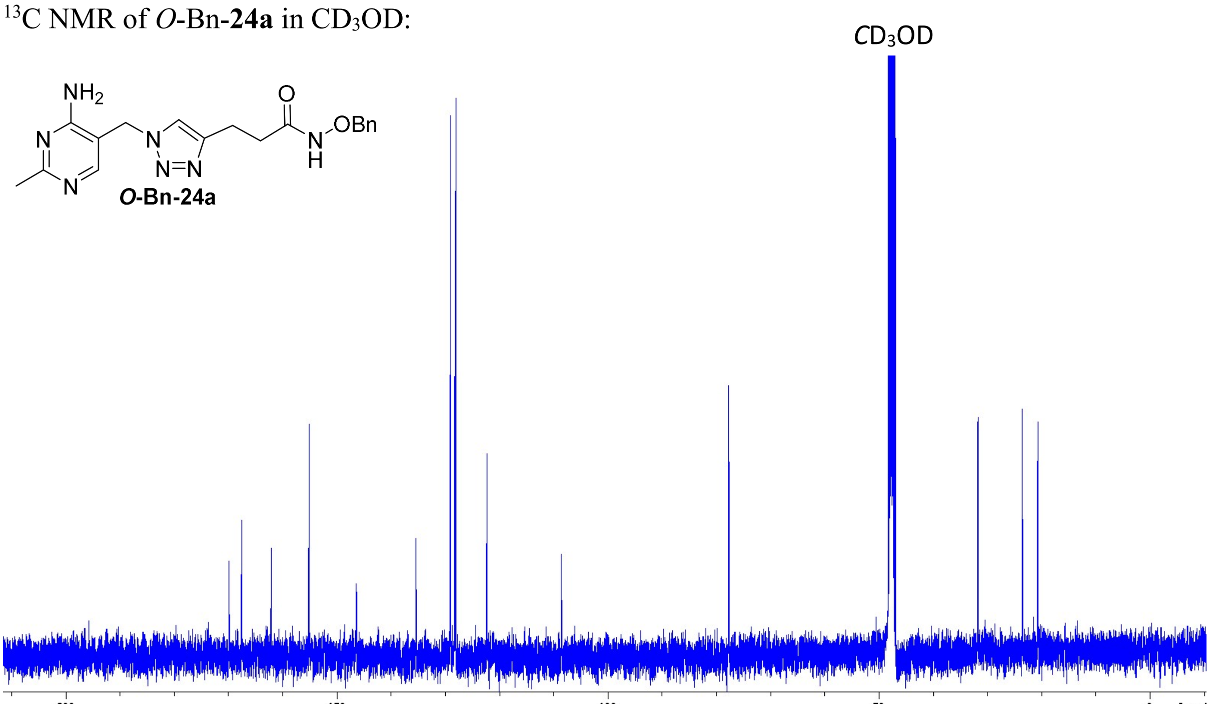

^{1}H NMR of *O*-Bn-**24b** in CD_3OD:

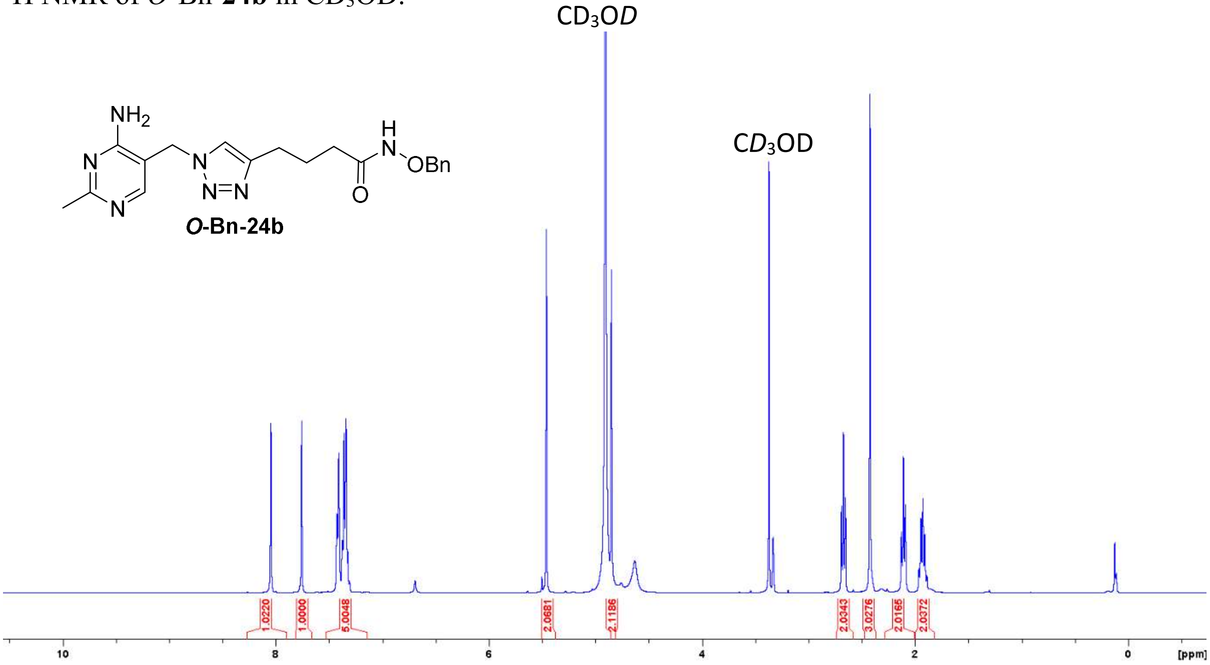

^{13}C NMR of *O*-Bn-**24b** in CD_3OD:

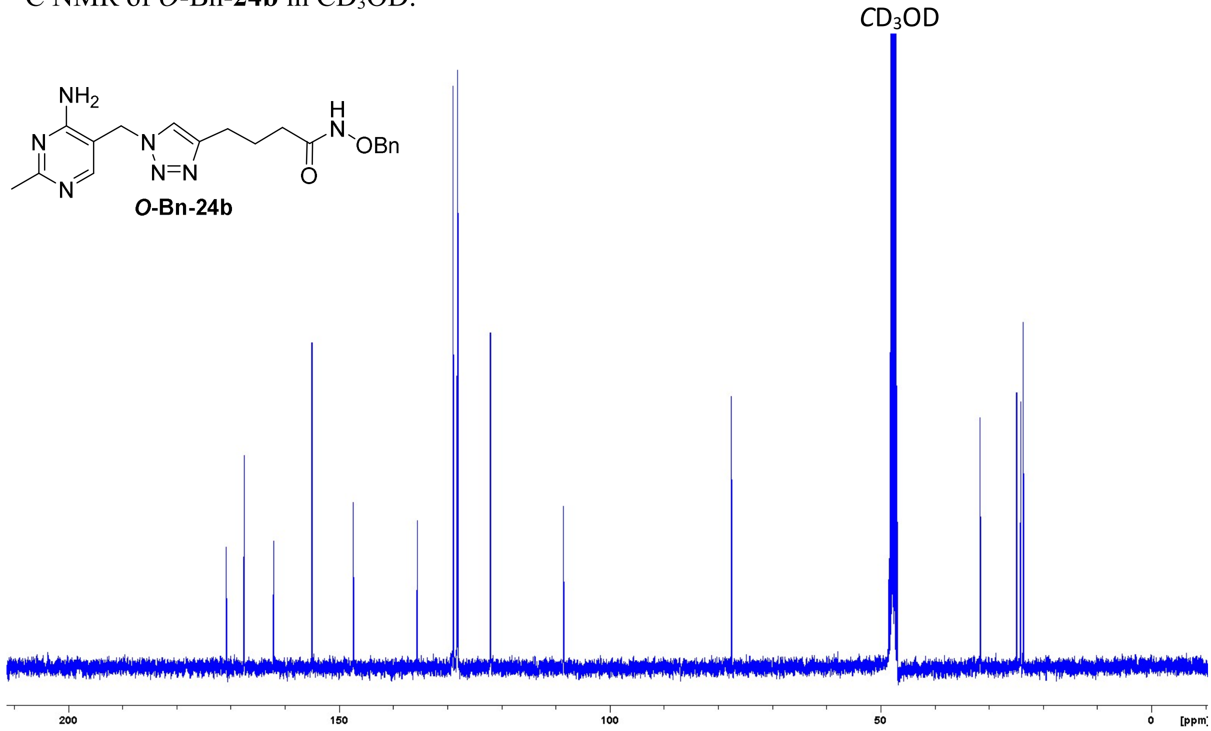

^{1}H NMR of *O*-Bn-**24c** in CD_3OD:

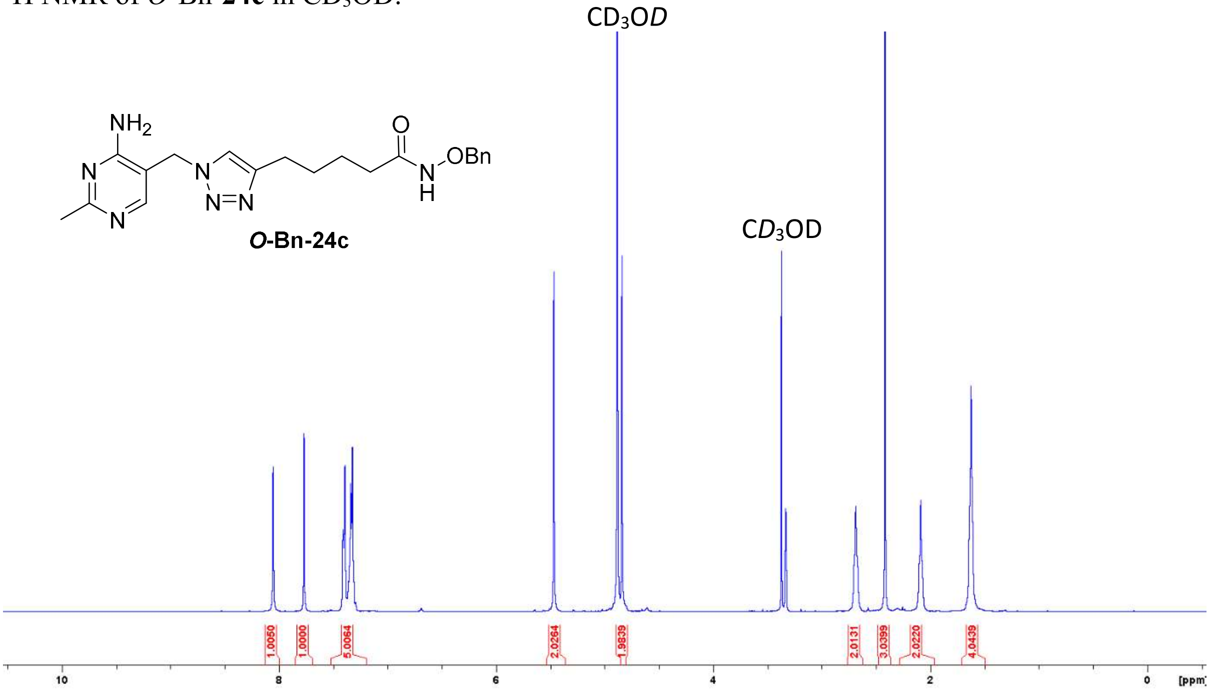

^{13}C NMR of *O*-Bn-**24c** in CD_3OD:

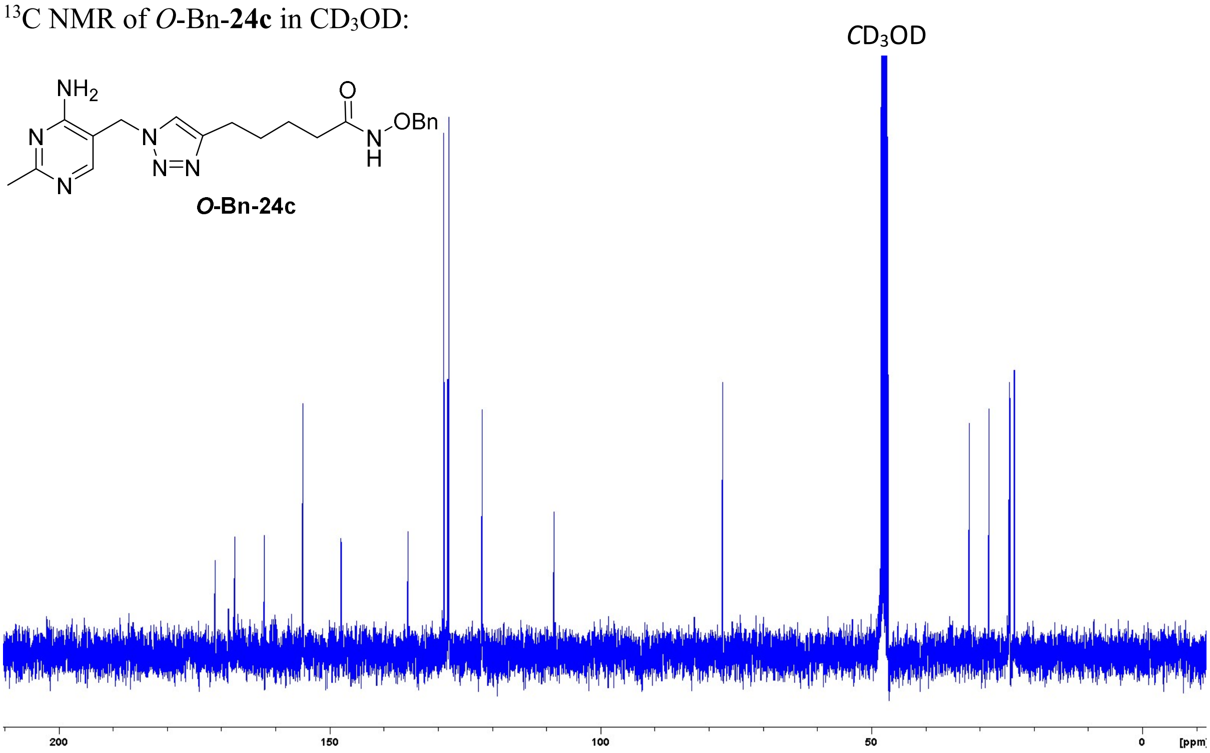

^{1}H NMR of **24a** in CD_3OD:

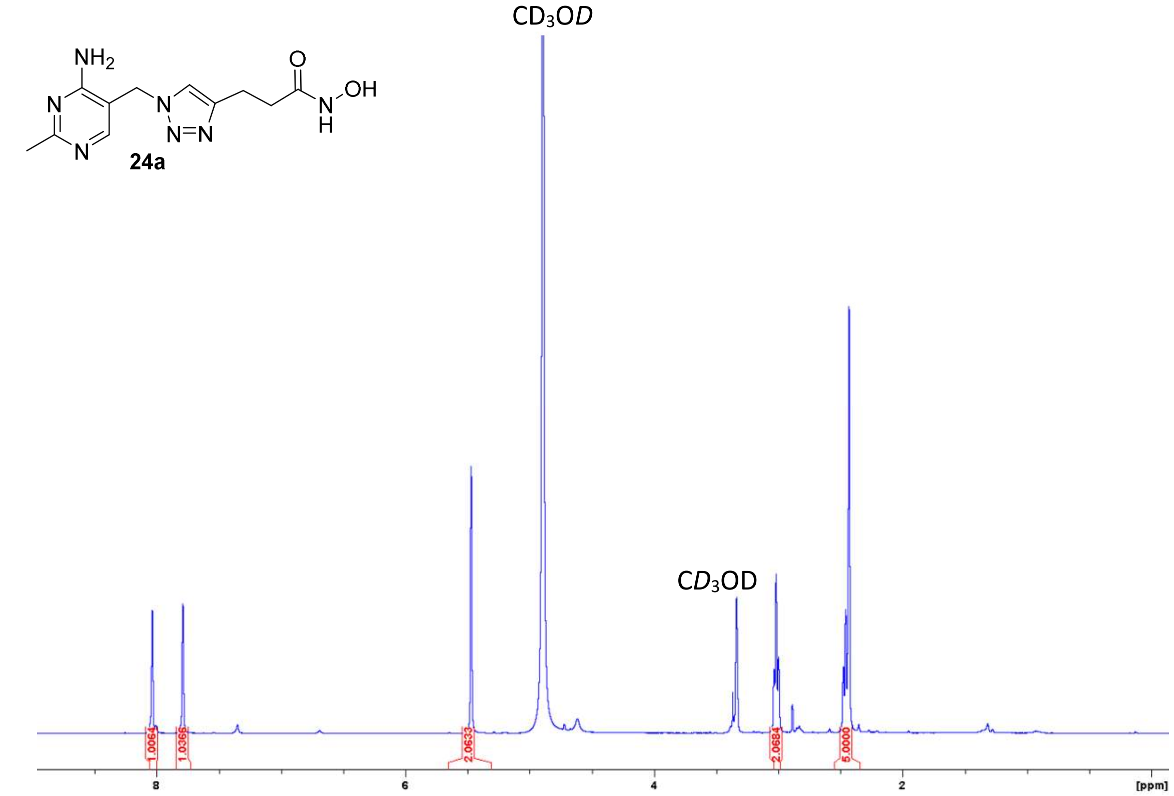

^{13}C NMR of **24a** in CD_3OD:

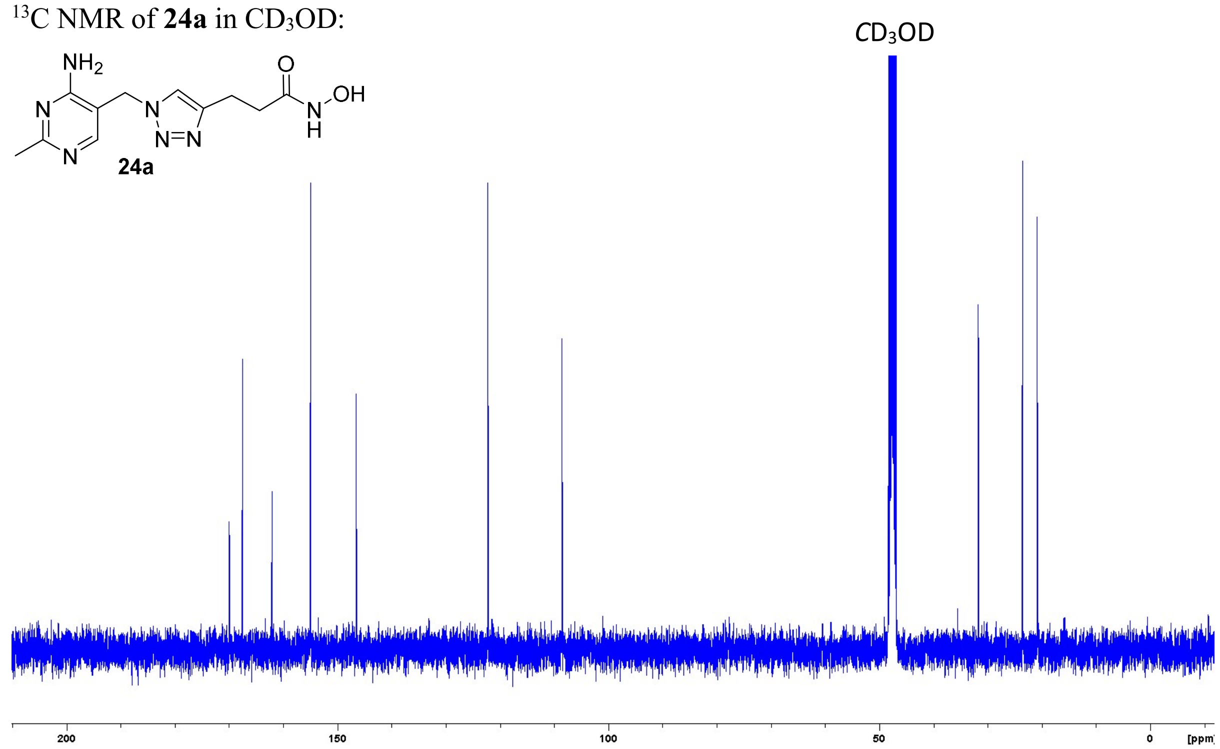

^{1}H NMR of **24b** in CD_3OD:

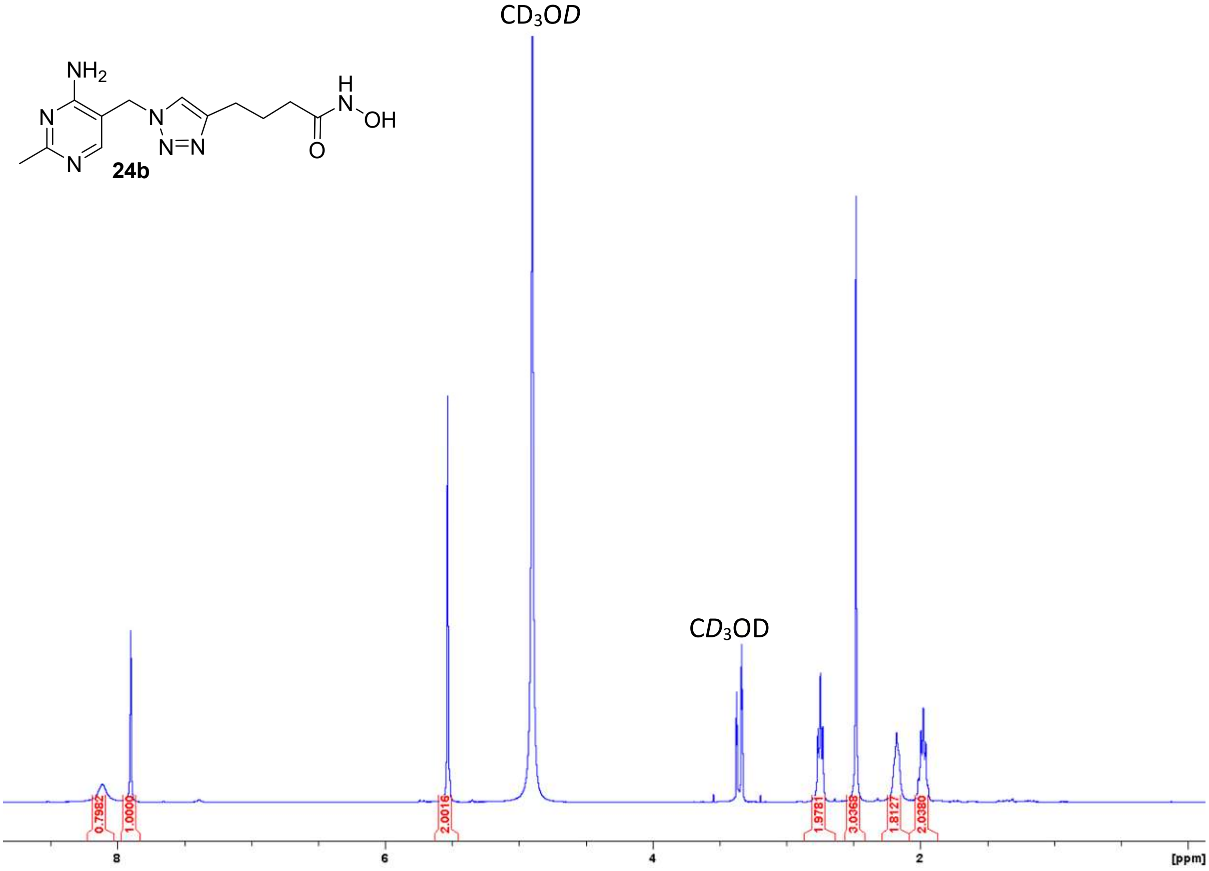

^{13}C NMR of **24b** in CD_3OD:

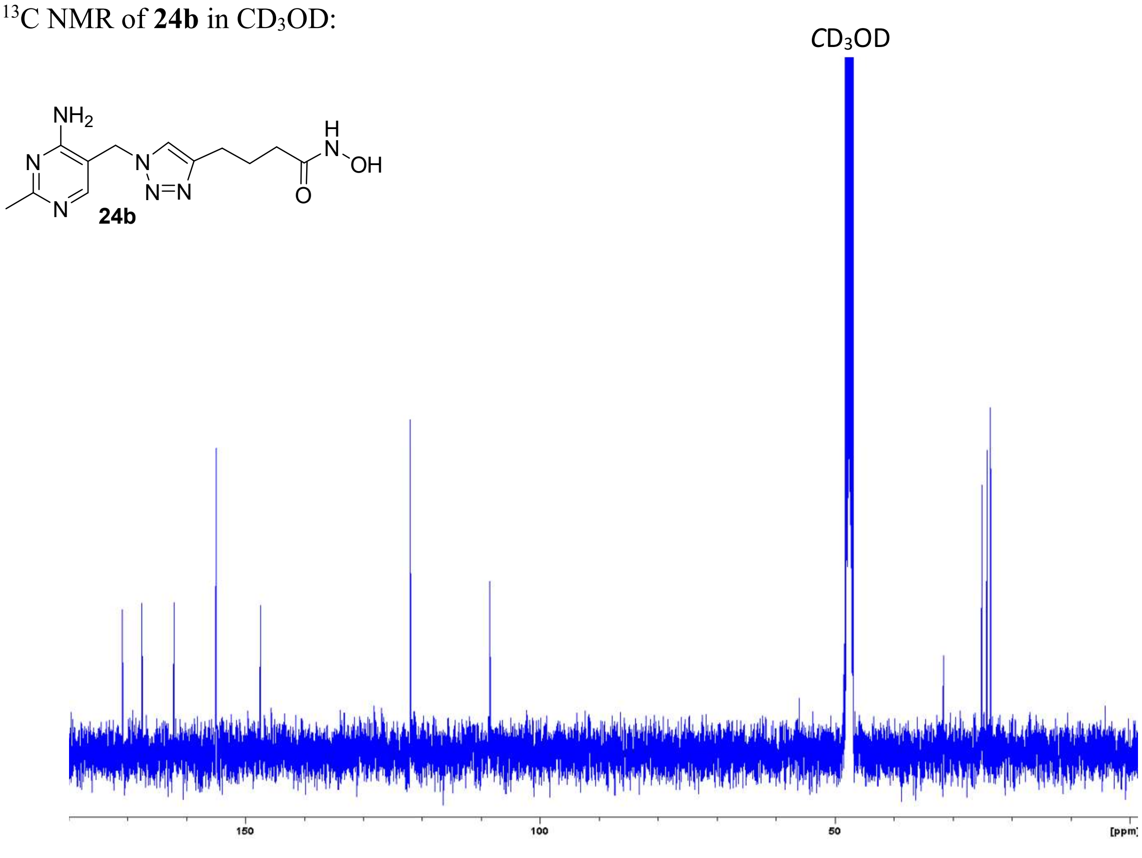

^{13}C DEPT-135 NMR of **24b** in CD_3OD:

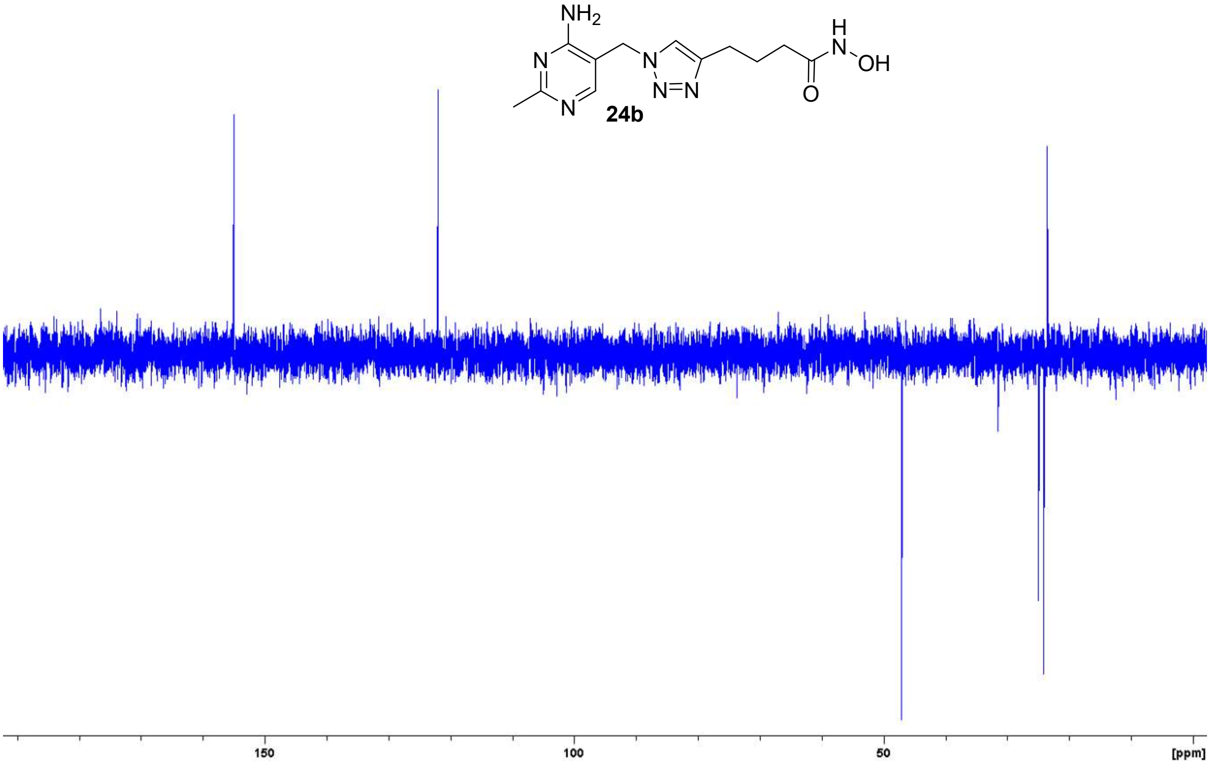

^{1}H NMR of **24c** in CD_3OD:

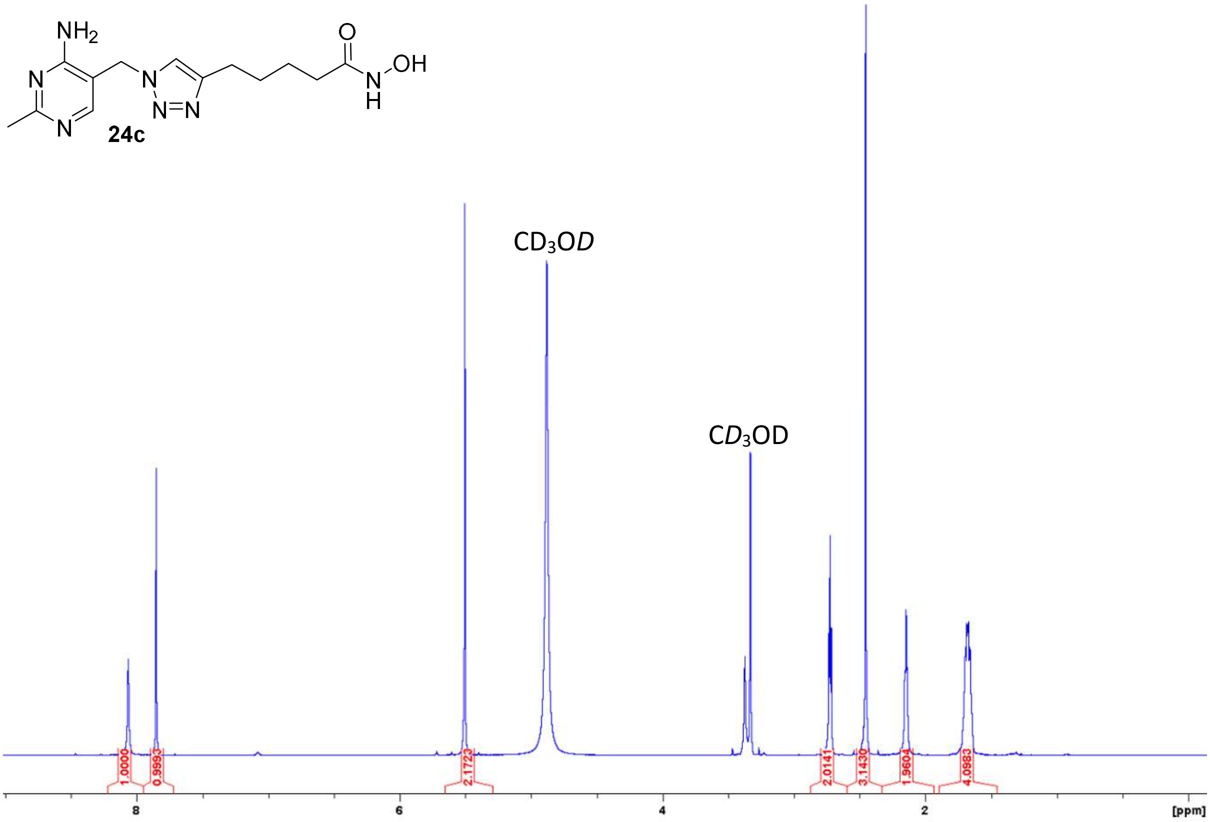

^{13}C NMR of **24c** in CD_3OD:

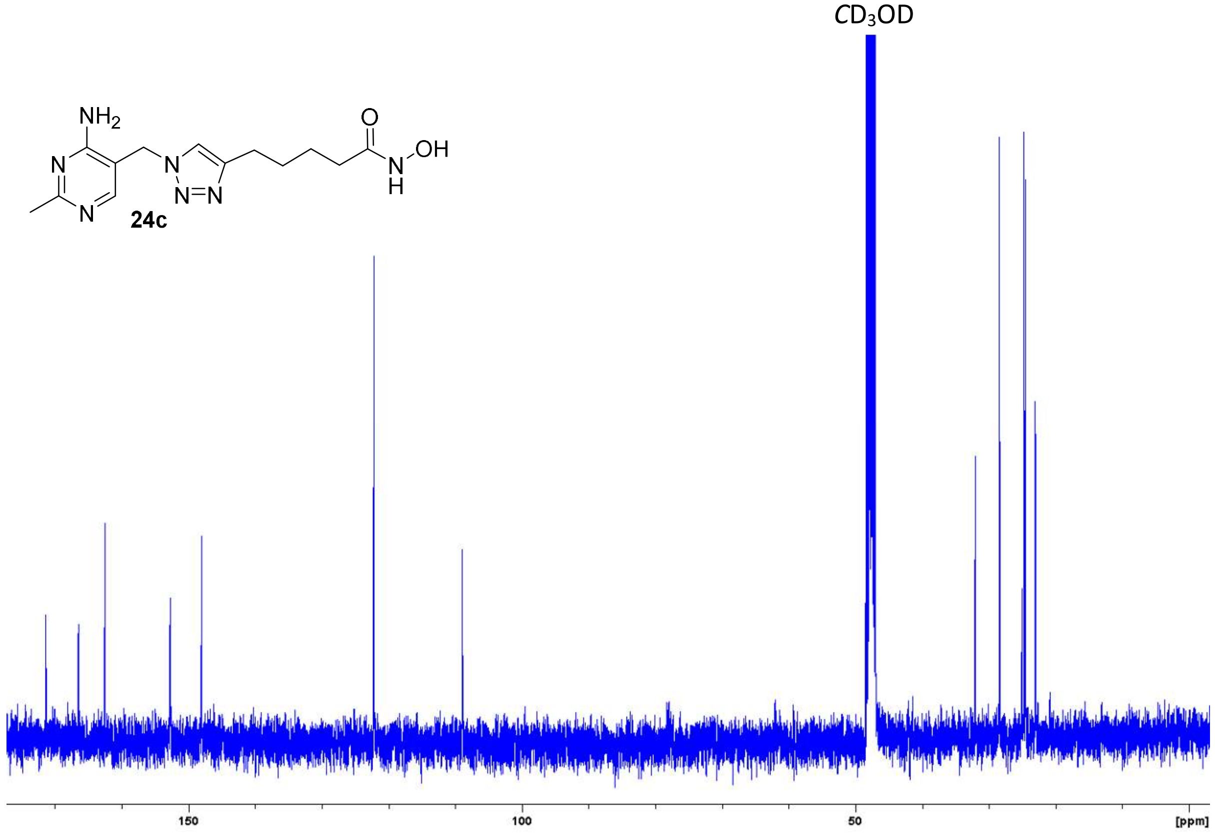

^{13}C DEPT-135 NMR of **24c** in CD_3OD:

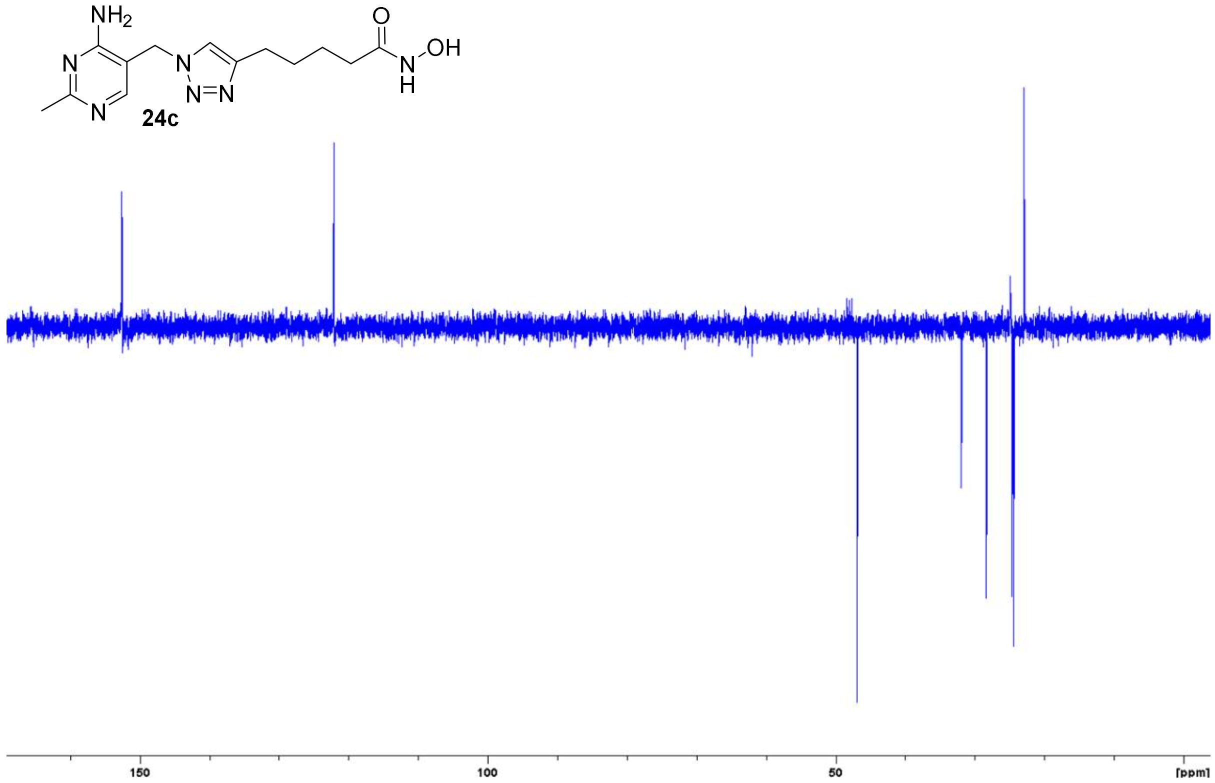

^{1}H NMR of Boc-**26a** in CD_3OD:

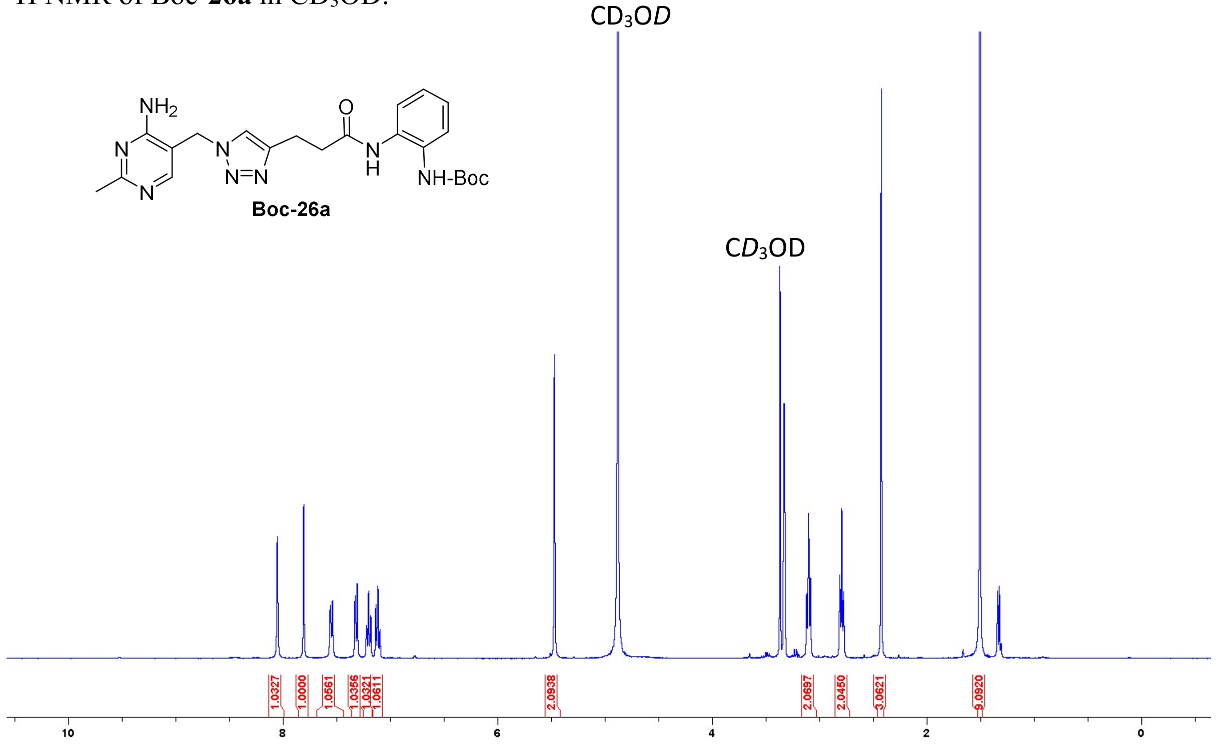

^{13}C NMR of Boc-**26a** in CD_3OD:

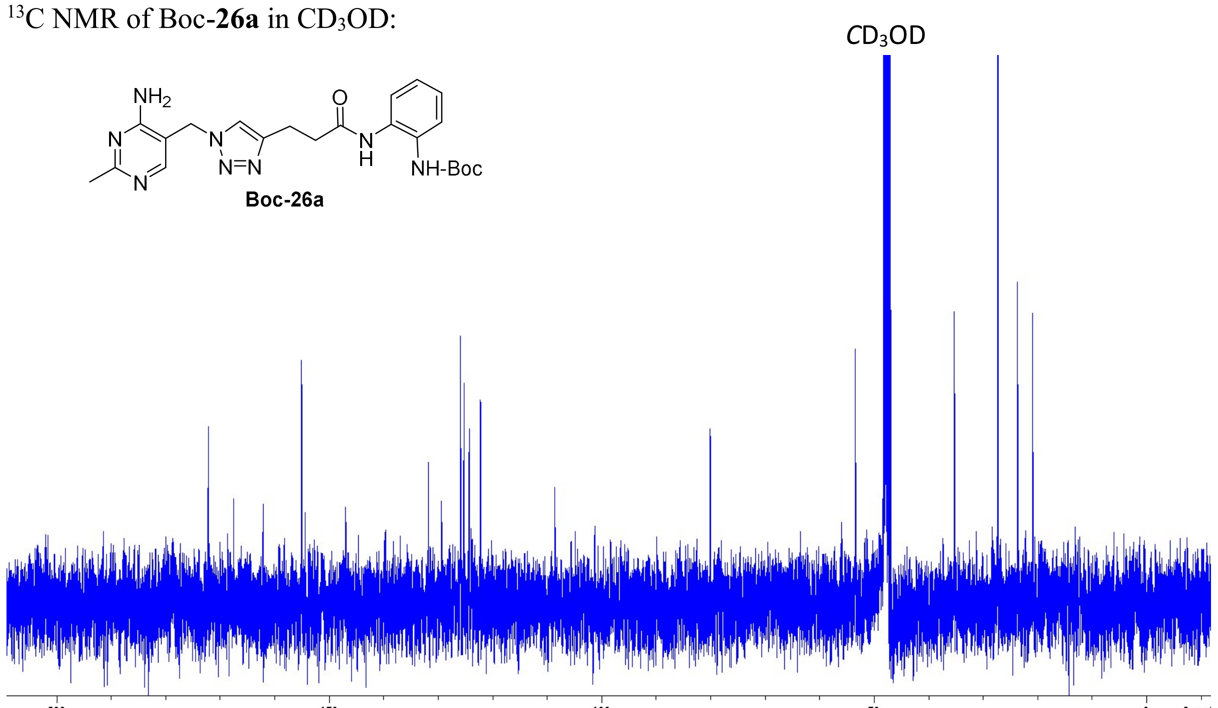

^{1}H NMR of Boc-**26b** in CD_3OD:

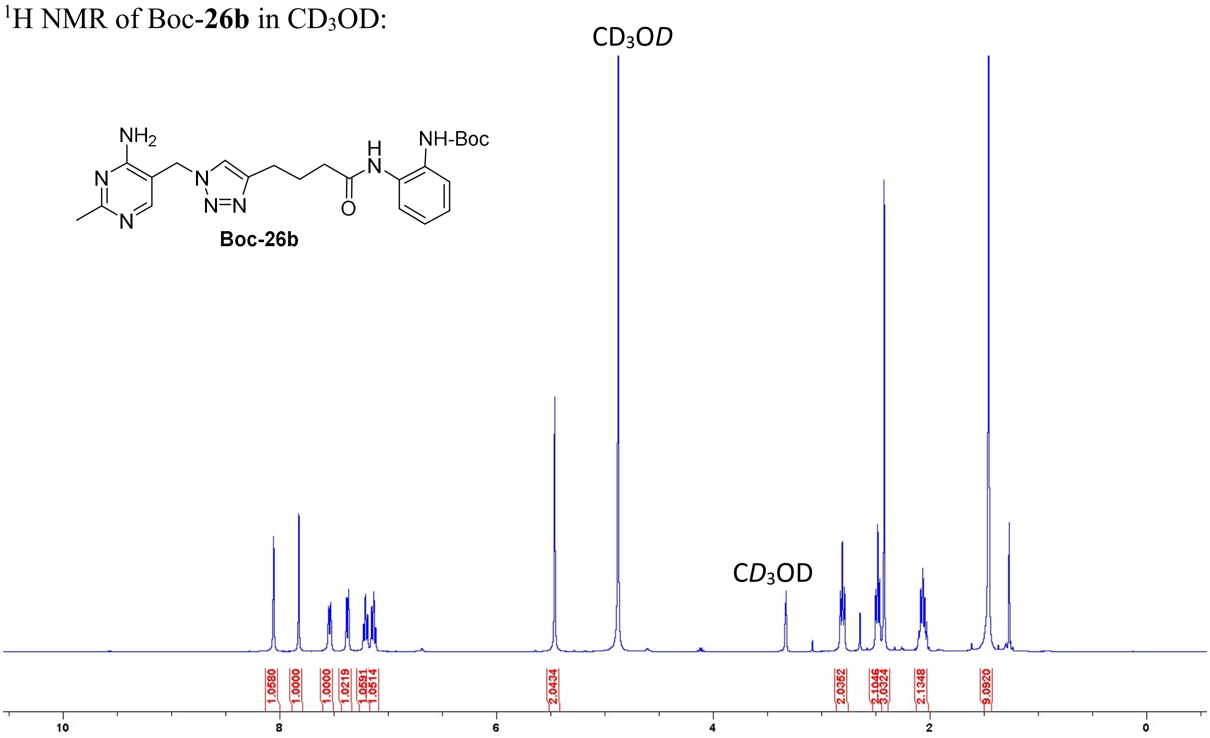

^{13}C NMR of Boc-**26b** in CD_3OD:

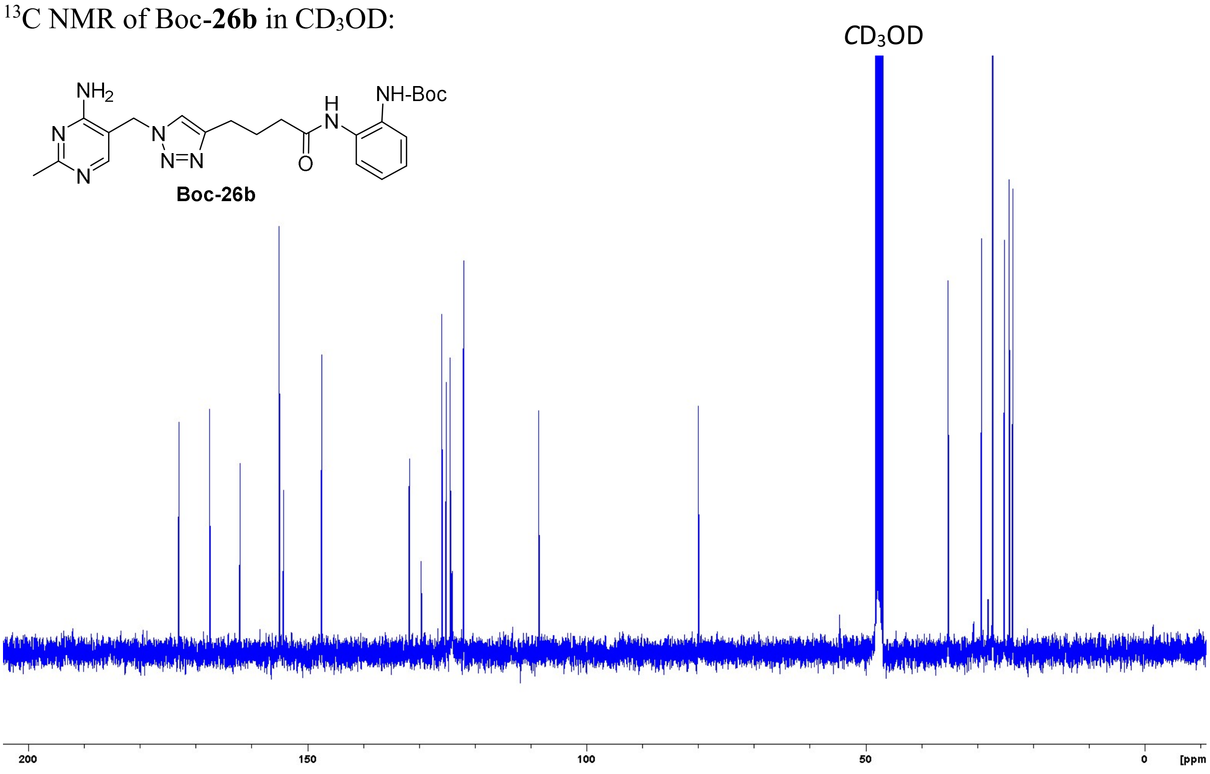

^{1}H NMR of Boc-**26c** in CD_3OD:

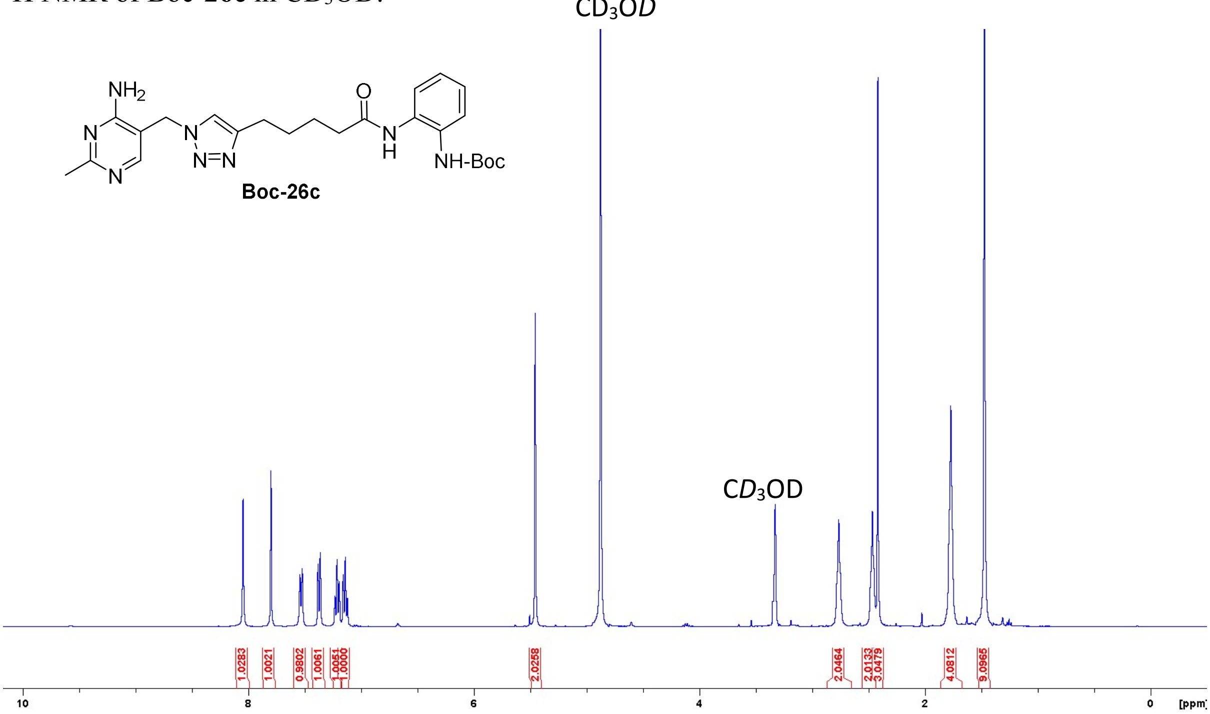

^{13}C NMR of Boc-**26c** in CD_3OD:

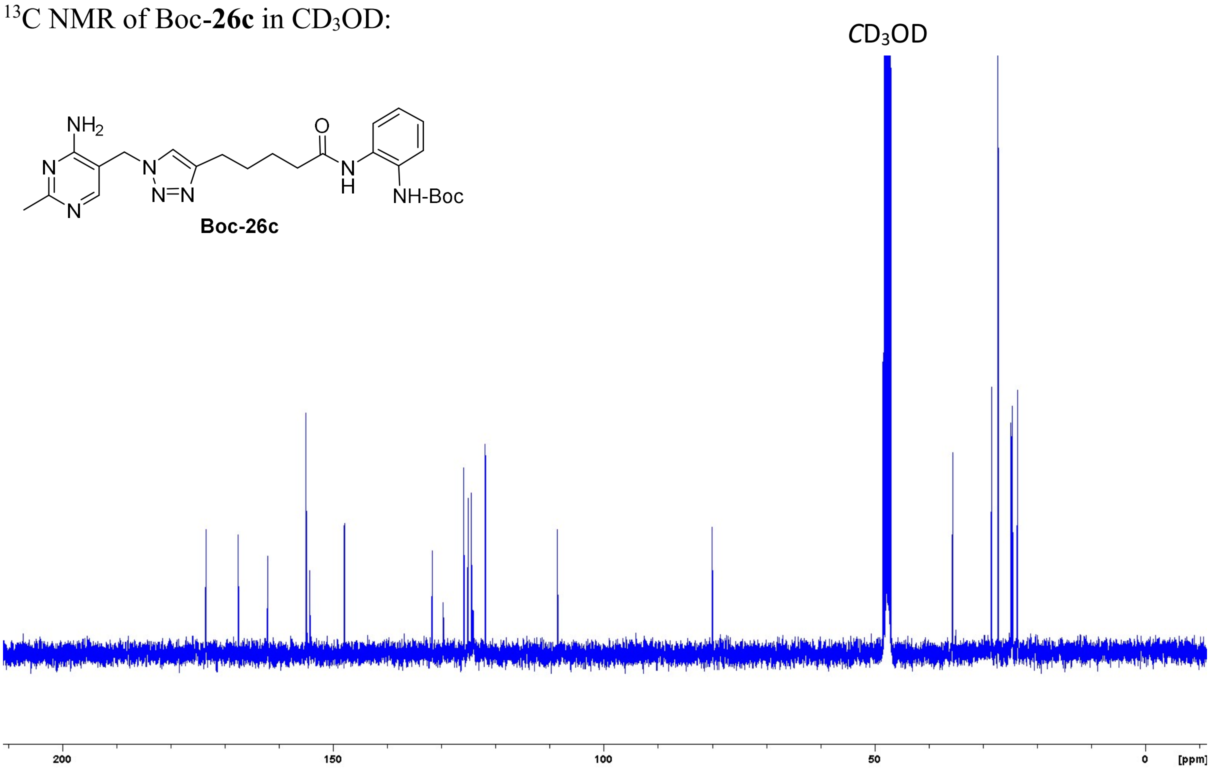

^{1}H NMR of **26a** in CD_3OD:

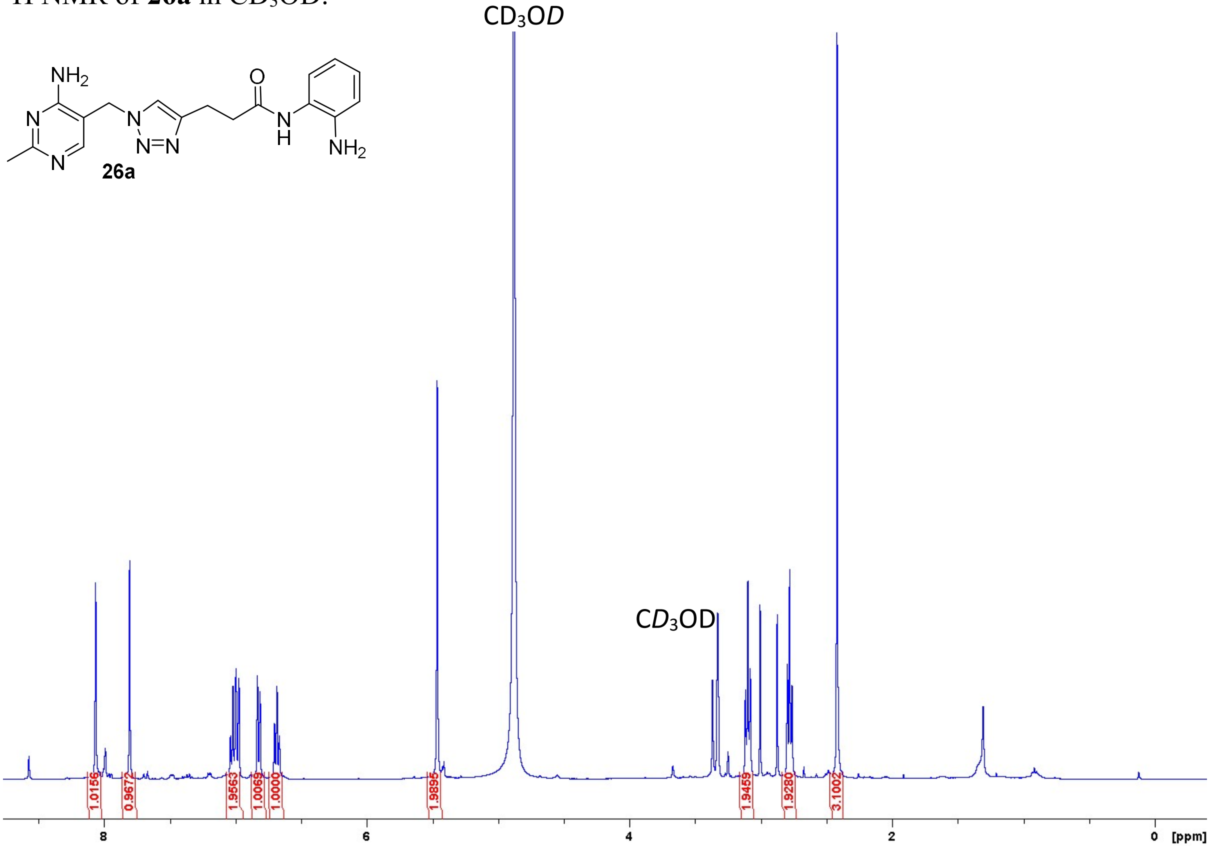

^{13}C NMR of **26a** in CD_3OD:

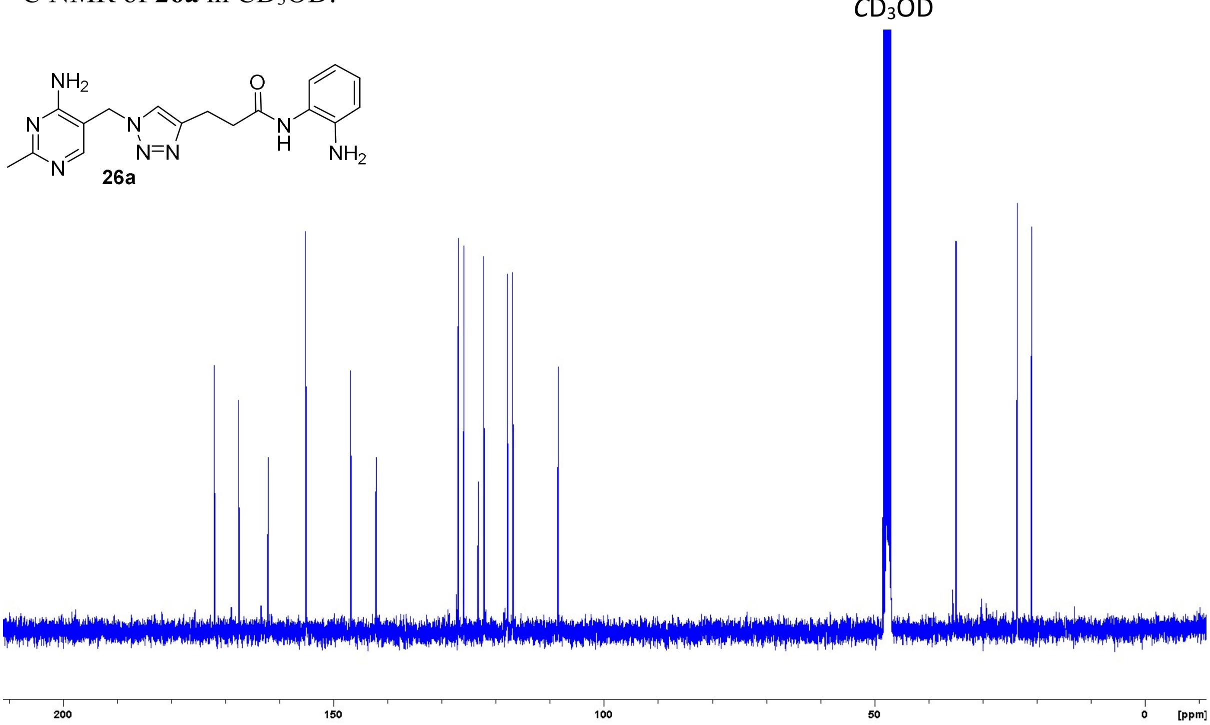

^{1}H NMR of **26b** in CD_3OD:

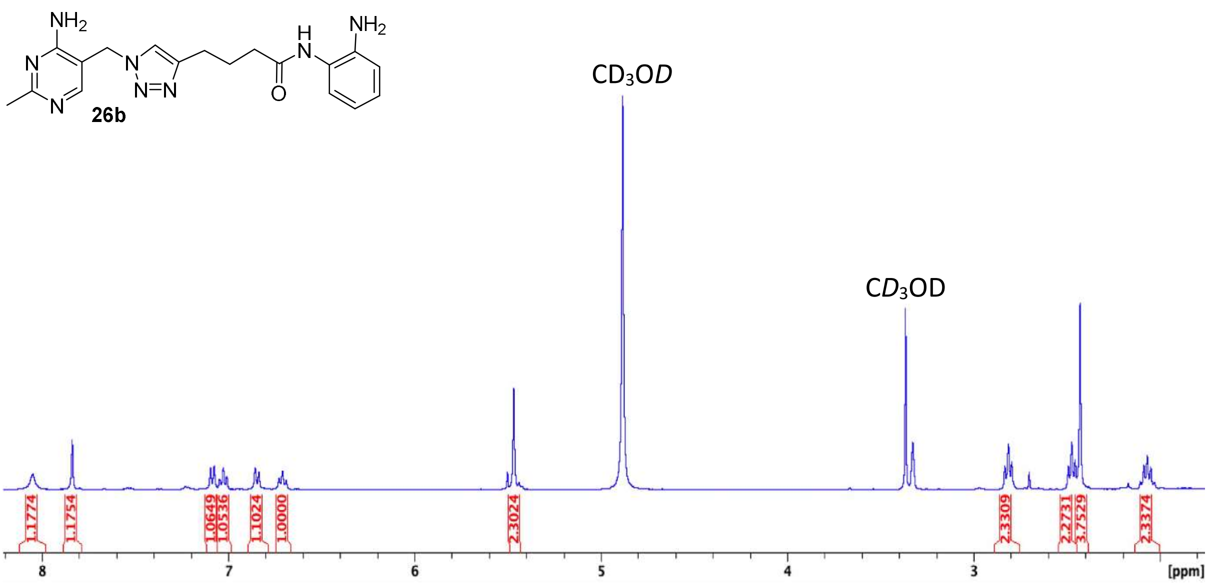

^{13}C NMR of **26b** in CD_3OD:

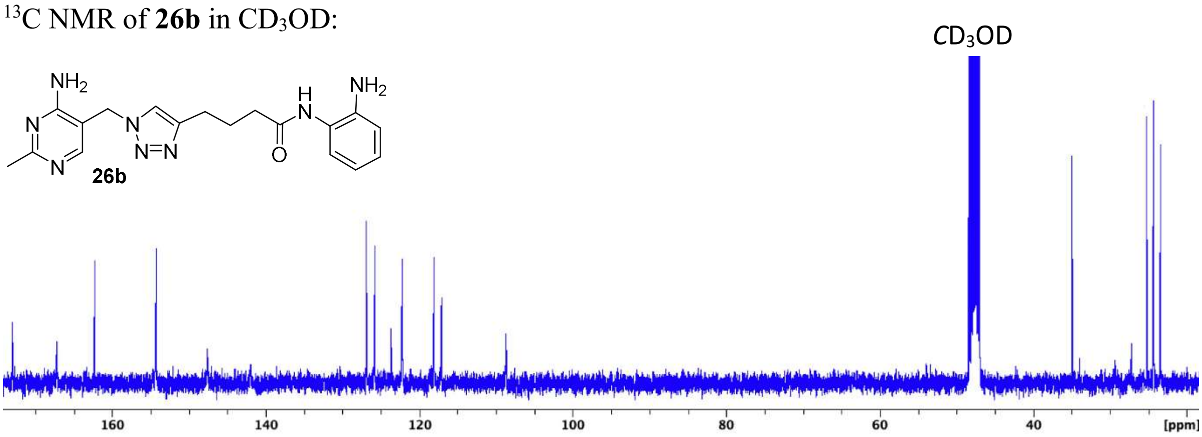

^{1}H NMR of **26c** in CD_3OD:

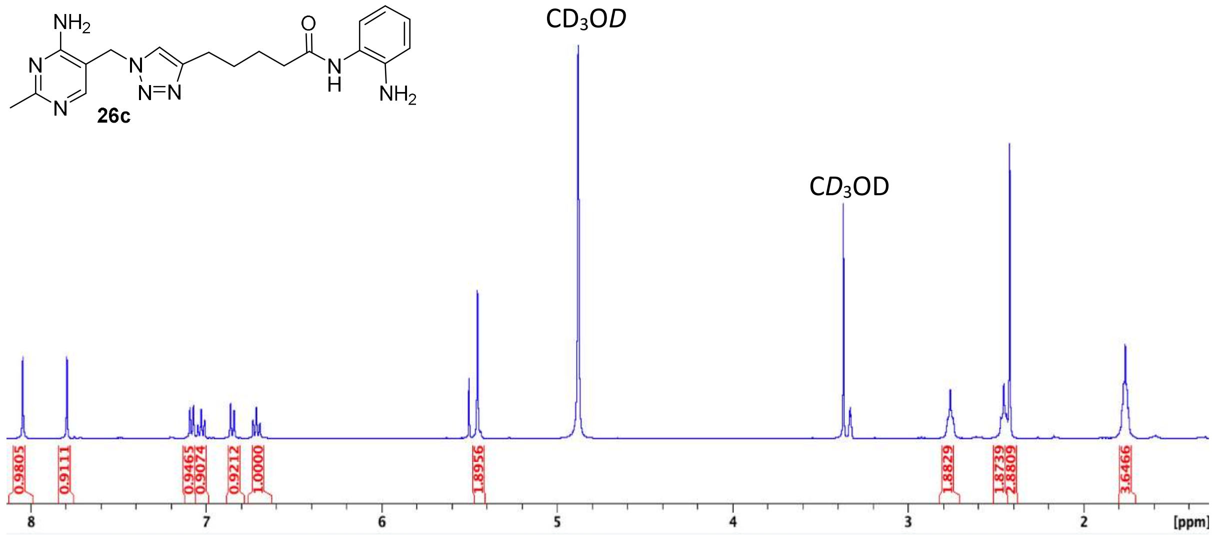

^{13}C NMR of **26c** in CD_3OD:

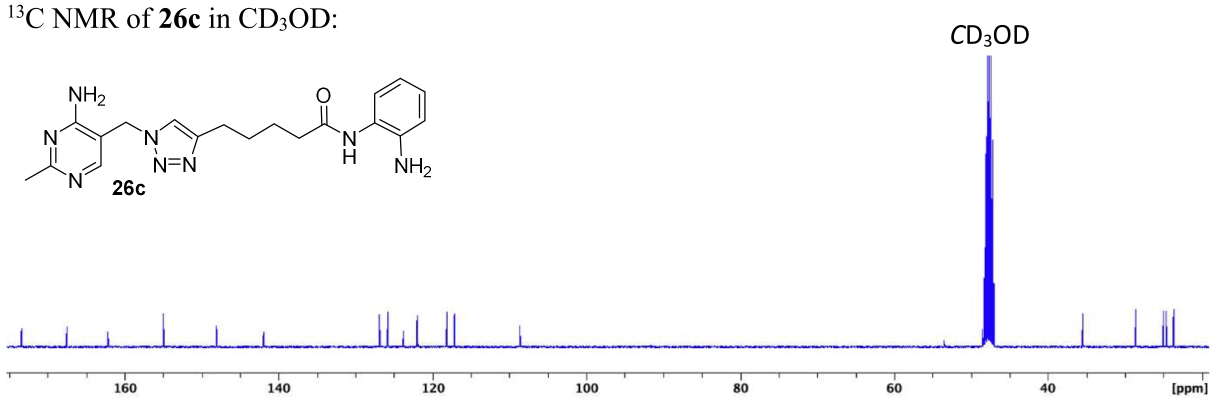

References

(1) Chan, A.H.Y.; Ho, T.C.S; Parle, D.; Leeper, F.J. Furan-based inhibitors of pyruvate dehydrogenase: SAR study, biochemical evaluation and computational analysis. *Org. Biomol. Chem.* **2023**, 21 (8), 1755-1763 https://doi.org/10.1039/D2OB02272A.

(2) Chan, A.H.Y.; Ho, T.C.S.; Agyei-Owusu, K.; Leeper, F.J. Synthesis of Pyrrothiamine, a Novel Thiamine Analogue, and Evaluation of Derivatives as Potent and Selective Inhibitors of Pyruvate Dehydrogenase. *Org. Biomol. Chem.* **2022**, *20* (45), 8855-8858. https://doi.org/10.1039/D2OB01819E.

(3) Jahn, B.; Jonasson, N. S. W.; Hu, H.; Singer, H.; Pol, A.; Good, N. M.; den Camp, H. J. M. O.; Martinez-Gomez, N. C.; Daumann, L. J. Understanding the Chemistry of the Artificial Electron Acceptors PES, PMS, DCPIP and Wurster's Blue in Methanol Dehydrogenase Assays. *J. Biol. Inorg. Chem.* **2020**, *25* (2), 199–212. https://doi.org/10.1007/s00775-020-01752-9.

(4) Walsh, D. A.; Cooper, R. H.; Denton, R. M.; Bridges, B. J.; Randle, P. J. The Elementary Reactions of the Pig Heart Pyruvate Dehydrogenase Complex. A Study of the Inhibition by Phosphorylation. *Biochem. J.* **1976**, *157* (1), 41–67. https://doi.org/10.1042/bj1570041.

(5) Mann, S.; Perez Melero, C.; Hawksley, D.; Leeper, F. J. Inhibition of Thiamin Diphosphate Dependent Enzymes by 3-Deazathiamin Diphosphate. *Org. Biomol. Chem.* 2004, *2* (12), 1732. https://doi.org/10.1039/b403619k.

(6) Iqbal, A.; Sahraoui, E.-H.; Leeper, F. J. Gold(I)-Catalysed Synthesis of a Furan Analogue of Thiamine Pyrophosphate. *Beilstein J. Org. Chem.* **2014**, *10*, 2580–2585. https://doi.org/10.3762/bjoc.10.270.

(7) Meanwell, N. A. Improving Drug Candidates by Design: A Focus on Physicochemical Properties As a Means of Improving Compound Disposition and Safety. *Chem. Res. Toxicol.* **2011**, *24* (9), 1420–1456. https://doi.org/10.1021/tx200211v.

(8) Merk, D.; Grisoni, F.; Friedrich, L.; Gelzinyte, E.; Schneider, G. Computer-Assisted Discovery of Retinoid X Receptor Modulating Natural Products and Isofunctional Mimetics. *J. Med. Chem.* **2018**, *61* (12), 5442–5447. https://doi.org/10.1021/acs.jmedchem.8b00494.

(9) Chan, A. H. Y.; Fathoni, I.; Ho, T.; Saliba, K. J.; Leeper, F. J. Thiamine Analogues as Inhibitors of Pyruvate Dehydrogenase and Discovery of a Thiamine Analogue with Non-Thiamine Related Anti-plasmodial Activity. *RSC Med. Chem.* **2022**, *13*, 817-821. https://doi.org/10.1039/D2MD00085G.

(10) Allen, R. J. W.; Kirk, K. Plasmodium Falciparum Culture: The Benefits of Shaking. *Molec. Biochem. Parasitol.* **2010**, *169* (1), 63–65. https://doi.org/10.1016/j.molbiopara.2009.09.005.

(11) Tjhin, E. T.; Spry, C.; Sewell, A. L.; Hoegl, A.; Barnard, L.; Sexton, A. E.; Siddiqui, G.; Howieson, V. M.; Maier, A. G.; Creek, D. J.; Strauss, E.; Marquez, R.; Auclair, K.; Saliba, K. J. Mutations in the Pantothenate Kinase of Plasmodium Falciparum Confer Diverse Sensitivity Profiles to Antiplasmodial Pantothenate Analogues. *PLoS Pathog.* **2018**, *14* (4), e1006918. https://doi.org/10.1371/journal.ppat.1006918.

(12) Johnson, J. D.; Dennull, R. A.; Gerena, L.; Lopez-Sanchez, M.; Roncal, N. E.; Waters, N. C. Assessment and Continued Validation of the Malaria SYBR Green I-Based Fluorescence Assay for Use in Malaria Drug Screening. *Antimicrob. Agents Chemother.* **2007**, *51* (6), 1926–1933. https://doi.org/10.1128/AAC.01607-06.

(13) Howieson, V. M.; Tran, E.; Hoegl, A.; Fam, H. L.; Fu, J.; Sivonen, K.; Li, X. X.; Auclair, K.; Saliba, K. J. Triazole Substitution of a Labile Amide Bond Stabilizes Pantothenamides and Improves Their Anti-plasmodial Potency. *Antimicrob. Agents Chemother.* **2016**, *60* (12), 7146–7152. https://doi.org/10.1128/AAC.01436-16.

(14) Erixon, K. M.; Dabalos, C. L.; Leeper, F. J. Synthesis and Biological Evaluation of Pyrophosphate Mimics of Thiamine Pyrophosphate Based on a Triazole Scaffold. *Org. Biomol. Chem.* **2008**, *6* (19), 3561. https://doi.org/10.1039/b806580b.

(15) Lobera, M.; Madauss, K. P.; Pohlhaus, D. T.; Wright, Q. G.; Trocha, M.; Schmidt, D. R.; Baloglu, E.; Trump, R. P.; Head, M. S.; Hofmann, G. A.; Murray-Thompson, M.; Schwartz, B.; Chakravorty, S.; Wu, Z.; Mander, P. K.; Kruidenier, L.; Reid, R. A.; Burkhart, W.; Turunen, B. J.; Rong, J. X.; Wagner, C.; Moyer, M. B.; Wells, C.; Hong, X.; Moore, J. T.; Williams, J. D.; Soler, D.; Ghosh, S.; Nolan, M. A. Selective Class IIa Histone Deacetylase Inhibition via a Nonchelating Zinc-Binding Group. *Nat. Chem. Biol.* **2013**, *9* (5), 319–325. https://doi.org/10.1038/nchembio.1223.
